# Nanoparticles as *Heterogeneous* Catalysts for ppm Pd-Catalyzed Aminations *in Water*

**DOI:** 10.1021/acssuschemeng.3c06527

**Published:** 2024-01-22

**Authors:** Karthik Iyer, Rahul Kavthe, Yuting Hu, Bruce H. Lipshutz

**Affiliations:** Department of Chemistry and Biochemistry, University of California, Santa Barbara, California 93106, United States

**Keywords:** aminations, nanoparticles, C–N bond formation, micellar catalysis, late-stage functionalization

## Abstract

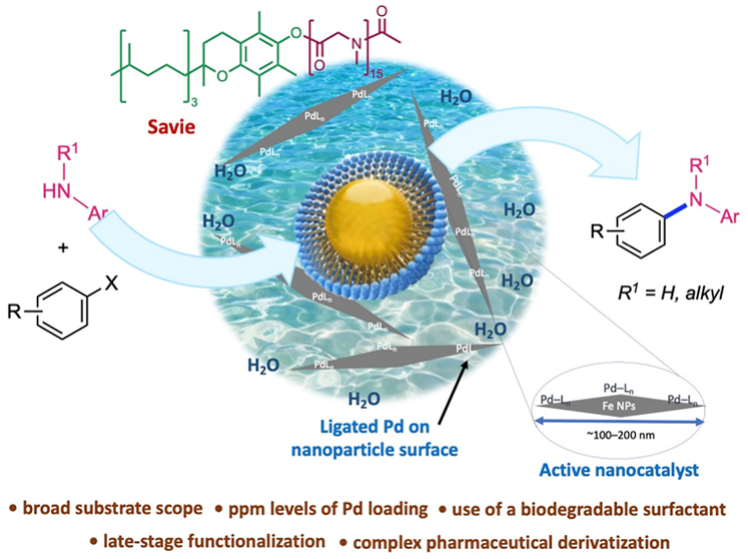

A general protocol employing heterogeneous catalysis has been developed that enables ppm of Pd-catalyzed C–N cross-coupling reactions under aqueous micellar catalysis. A new nanoparticle catalyst containing specifically ligated Pd, in combination with nanoreactors composed of the designer surfactant Savie, a biodegradable amphiphile, catalyzes C–N bond formations in recyclable water. A variety of coupling partners, ranging from highly functionalized pharmaceutically relevant APIs to educts from the Merck Informer Library, readily participate under these environmentally responsible, sustainable reaction conditions. Other key features associated with this report include the low levels of residual Pd found in the products, the recyclability of the aqueous reaction medium, the use of ocean water as an alternative source of reaction medium, options for the use of pseudohalides as alternative reaction partners, and associated low *E* factors. In addition, an unprecedented 5-step, one-pot sequence is presented, featuring several of the most widely used transformations in the pharmaceutical industry, suggesting potential industrial applications.

## Abstract 2

New nanoparticles containing parts per million levels of a palladium catalyst are useful for making carbon–nitrogen bonds found in pharmaceuticals.

## Introduction

Palladium-catalyzed aminations of aryl and heteroaryl halides have become a fundamental tool in organic synthesis for generating C–N bonds characteristic of natural products,^1−4^ pharmaceuticals,^1,5−7^ agrochemicals,^8,9^ and organic materials.^10−12^ Their occurrence, together with the changing times, where sustainability has become an increasingly important consideration, has led to a growing need for the development of methods that are not only general, mild, and selective for C(sp^2^)–N bond formation but also incorporate consideration of important principles of green chemistry, including safety, planetary resources, and environmental concerns. Notwithstanding the plethora of existing approaches that have been developed, group 10 transition metal-catalyzed aryl- and hetero-aryl aminations,^13,14^ along with Cu-catalyzed Ullmann couplings,^8,15−18^ have emerged as among the most widely used methods due to their versatility and functional group tolerance.^19,20^ A wide variety of dialkylbiarylphosphine ligands^21,22^ and various palladacycles incorporating these ligands^23−31^ have been found to catalyze couplings between aryl/heteroaryl halides and pseudo-halides with a broad range of amine nucleophiles.^32,33^ Notable examples of effective ligands include *N*-heterocyclic carbene (NHC)-based pre-catalysts,^24,27,34^ the use of which has led to couplings of even secondary amines with unfunctionalized aryl halides. While these are amenable to relatively low catalyst loadings, they tend to involve relatively simple amines and take place in waste-generating organic solvents. While a new oxidative addition complex has been developed that demonstrates good reactivity with a wide range of aryl and pseudohalide electrophiles, including base-sensitive 5-membered heterocycles,^35,36^ there are several aspects that must be considered from a life cycle analysis perspective. That is, the synthesis of the ligand (GPhos) as well as the oxidative addition complex involves multiple steps. Moreover, it is especially costly at the research level of usage since neither species is commercially available on scale.^35^ Hence, there are opportunities for the development of new tools in a growing toolbox that can eventually replace traditional approaches that rely on dangerous organic solvent-based methodologies at (usually) elevated temperatures (e.g., in toluene, 1,4-dioxane, and DME).^13,23−33^ Extended reaction times are also commonplace, leading to both substantial investments of energy, which can be costly, as well as potential issues with resulting impurity profiles of the aminated products that require additional time and effort to arrive at the purified material. Particularly noteworthy, as practiced over the past 25 years,^37^ is that aminations typically require 1–10 mol % palladium, thereby consuming platinum group metals.^38^

**Scheme 1:**
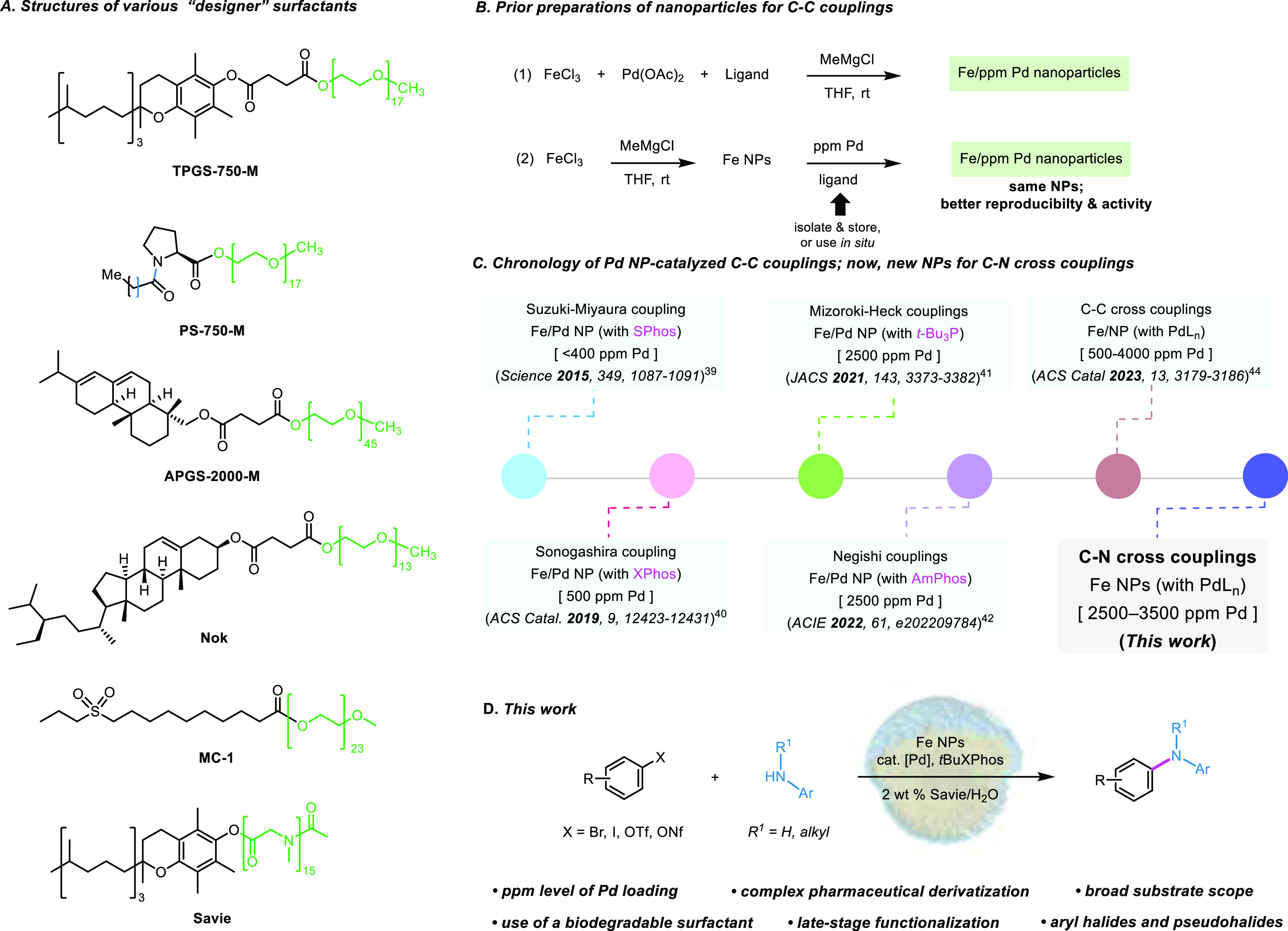
Overall Picture Illustrating Various Aspects Associated with Low Loadings of Pd NP-Catalyzed Aminations in Water

Several technologies based on water as a recyclable reaction medium have been continuously developed over the past 15 years. Given the water-insoluble status of most organic substrates and catalysts, inclusion of small amounts (typically 2–3 wt %) of a nonionic surfactant that self-assembles into nanometer micelles that act as nanoreactors by virtue of their lipophilic cores enables the desired couplings to take place, although exactly where in the micelles the species present in the medium is not known with certainty. Both water-insoluble substrates and catalysts localize therein, insulated from interactions with the surrounding aqueous medium. The close proximity of substrates and catalysts within the micellar cores leads to characteristically high concentrations (usually ≥2 M), leading to mild reaction conditions while enhancing reaction rates.^39−44^ Several surfactants, including the vitamin E-based amphiphile TPGS-750-M,^45^ the sulfone-based MC-1,^46^ the β-sitosterol-derived Nok,^47^ the fatty acid/proline-based PS-750-M from Handa and co-workers,^48^ and the rosin-based APGS-2000-M from the Huang group,^59^ use polyethylene glycol (PEG, or its monomethylated version, MPEG) as the hydrophilic subsection (see Scheme 1A). Our most recent addition to this select group is Savie, replacing MPEG with a polypeptoid derived from polysarcosine, which serves as a “drop-in” replacement to TPGS-750-M.^50^ The derived micellar array derived from Savie not only enables a variety of couplings, including aminations run in water under *homogeneous* conditions at the ppm level of Pd, but also provides a biodegradable solution for the downstream processing of the aqueous reaction medium.^49^

In appreciation of the preference by industry for heterogeneous catalysis,^51^ we disclosed a protocol back in 2015 involving the preparation of Fe/ppm Pd nanoparticles (NPs) derived from inexpensive FeCl_3_ containing ppm levels of Pd (either naturally occurring by via the addition of Pd(OAc)_2_)^52^ with MeMgCl, mixed in (recoverable at scale) tetrahydrofuran (THF) in the presence of SPhos as ligand.^53^ (Scheme 1B). This resulting heterogeneous reaction mixture is very effective at mediating complex Suzuki–Miyaura couplings in water at low loadings of palladium (i.e., 0.04 mol %), leading to C(sp^2^)–C(sp^2^) bonds under mild aqueous conditions (Scheme 1C). Since this report, we have shown that by simply changing the amount of Pd and the type of ligand, other Pd-catalyzed *C–C bond-forming reactions* can be facilitated using these NPs (i.e., Sonogashira,^54^ Mizoroki–Heck,^55^ and Negishi^56^ couplings; Scheme 1C).^57−60^ To obviate the issues of metal and/or ligand oxidation, which could compromise NP activity, a new protocol was developed for initially preparing NPs from the same inexpensive FeCl_3_ and MeMgCl, *albeit in the absence of the Pd and ligand*.^61^ We have already shown that the introduction of both [i.e., Pd(OAc)_2_ and SPhos] *after* initial NP formation leads to the same catalyst makeup. Hence, by simply allowing the NPs to equilibrate in the aqueous micellar medium containing ppm Pd and ligand, the “sculpting” process occurs, altering the ca. 5 nm spherical NPs into >100 nm rods.^55,61^ These newly fashioned, freshly prepared NPs are even more effective, catalyzing parts per million level Pd cross coupling reactions resulting in several types of key C–C bonds. In synthetic applications toward complex products, usually both the electrophilic and nucleophilic components are functionalized. Therefore, it is an important goal to address challenging substrate combinations that use relatively low catalyst loadings, especially for process development and manufacturing at scale.

Another important factor beyond catalyst cost is the removal of residual Pd from the product, which the FDA limits to 10 ppm/dose/day.^62^ Since at scale the cost of materials (reagents, catalysts, solvent, and starting materials) usually contributes 20–45%,^63^ time may also become a substantial determinant of economic viability. For these reasons, our goal was to develop not only a green technology for aminations that is generally applicable to functionalized aryl halides that requires only parts per million levels of Pd, but also one that takes place efficiently in a recyclable aqueous medium under relatively mild conditions. Thus, we now report on a new process for aminations featuring a previously unknown *heterogeneous protocol* involving ppm levels of a relatively inexpensive and commercially available catalyst, all made possible by a biodegradable nonionic surfactant Savie (which has not been used previously for heterogeneous catalysis; Scheme 1D).

## Results and Discussion

### Optimization of Aminations between Aryl Bromides and Aromatic Amines

Model studies were initiated using 5-bromo-2,2-difluorobenzo[*d*][1,3]dioxole (**1a**) and 3-aminoacetophenone (**1b**) in 2 wt % Savie as the reaction medium. Since many known aryl aminations utilize alkoxide bases,^64−68^ potassium *t*-butoxide (KO*t*Bu) was selected for this purpose. Previous efforts using Fe/ppm Pd NP-catalyzed cross couplings leading to C–C bond formation^53−56,61^ suggested an initial investment of 0.25 mol % Pd(OAc)_2_ (2500 ppm) might suffice. The key to success, however, was the eventual determination of the optimum ligand for Pd that efficiently mediated C–N bond construction under aqueous micellar conditions. As shown in Table 1, entry 15, *t*BuXPhos (2 mol %) was identified as the most effective ligand, affording arylamine **1** in 99% yield (as determined by ^1^H NMR; see Supporting Information, Section 3.1.1). Surprisingly, Takasago’s ligand *c*BRIDP (entry 11) was ineffective, notwithstanding its role in similar, albeit homogeneous, reactions reported by both Handa and co-workers^69^ and us.^70^ Likewise, MorDalPhos (entry 10)^71^ did not lead to aminated product **1**. Moreover, the recently developed ferrocene-based, air-stable ligands by Colacot and co-workers^72^ (entries 6 and 7), which when chelated with Pd display excellent activity in Fe/ppm Pd NP-catalyzed Suzuki–Miyaura homogeneous reactions,^61^ appear to be unsuited for these aminations.^73^

**Table 1:**
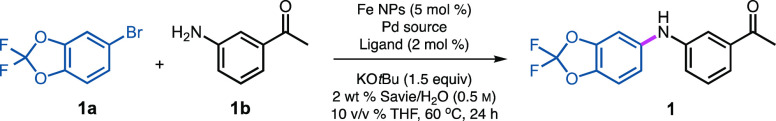
Optimization of Aminations for Aromatic Amines

entry^a^	source of Pd	ligand (2 mol %)	base	yield (%)^b^
1	Pd(OAc)_2_ (0.25 mol %)	XPhos	KO*t*Bu	23
2	Pd(OAc)_2_ (0.25 mol %)	*rac*-BINAP	KO*t*Bu	20
3	Pd(OAc)_2_ (0.25 mol %)	XantPhos	KO*t*Bu	10
4	Pd(OAc)_2_ (0.25 mol %)	*t*-BuXPhos	KO*t*Bu	90
5	Pd(OAc)_2_ (0.25 mol %)	P*t*-Bu_3_	KO*t*Bu	17
6	Pd(OAc)_2_ (0.25 mol %)	Fc(PAd_2_)	KO*t*Bu	c
7	Pd(OAc)_2_ (0.25 mol %)	Fc(P*t*Bu_2_)(PAd_2_)	KO*t*Bu	c
8	Pd(OAc)_2_ (0.25 mol %)	PAd_3_	KO*t*Bu	13
9	Pd(OAc)_2_ (0.25 mol %)	AdBrettPhos	KO*t*Bu	38
10	Pd(OAc)_2_ (0.25 mol %)	Mor-DalPhos	KO*t*Bu	c
11	Pd(OAc)_2_ (0.25 mol %)	*c-B*RIDP	KO*t*Bu	trace
12	[Pd(allyl)Cl]_2_ (0.125 mol %)	*t-*BuXPhos	KO*t*Bu	55%
13	[Pd(cinnamyl)Cl]_2_ (0.125 mol %)	*t-*BuXPhos	KO*t*Bu	35%
14	Pd(dba)_2_ (0.25 mol %)	*t-*BuXPhos	KO*t*Bu	99%
**15**	**[Pd(crotyl)Cl]**_**2**_**(0.125 mol %)**	***t-*BuXPhos**	**KO*t*Bu**	**99%**
16	[Pd(crotyl)Cl]_2_ (0.125 mol %)	*t-*BuXPhos	NaO*t*Bu	90
17	[Pd(crotyl)Cl]_2_ (0.125 mol %)	*t-*BuXPhos	Cs_2_CO_3_	62
18	[Pd(crotyl)Cl]_2_ (0.125 mol %)	*t-*BuXPhos	Et_3_N	84
19	[Pd(crotyl)Cl]_2_ (0.125 mol %)	*t-*BuXPhos	KOTMS	84

a Reactions were carried out at the 0.25 mmol scale.

b NMR yields were determined using 1,3,5-trimethoxybenzene as an internal standard.

c No product was observed.

Along with Pd(OAc)_2_, other sources of palladium, such as Pd(dba)_2_, were also screened. Included in this evaluation were Colacot’s π–allyl complexes,^74^ given their commercial availability and the bench stability of these Pd dimers (see Supporting Information, Table S2). Noteworthy was the observation that only 1250 ppm of Pd (0.125 mol %, or 2500 ppm total Pd) from dimeric [Pd(crotyl)Cl]_2_ was found to be the most efficient catalyst precursor, affording the coupled product a close to quantitative yield (entry 15; also see Supporting Information, Table S2). Surprisingly, under aqueous micellar conditions, the nature of the π–allyl species plays an important role with respect to the activity of the catalyst being formed in situ, as changing from the crotyl to either allyl (entry 12) or cinnamyl (entry 13) groups, respectively, significantly reduced the yields of **1**.

Furthermore, while Pd(dba)_2_ also afforded excellent yields (entry 14) of model product **1**, its air sensitivity led to issues of reproducibility and further studies. The selection of base was determined to also be yet another crucial parameter for arriving at high levels of conversion to coupled product **1** (Table 1). KO*t*Bu was found to be optimal (see Supporting Information, Table S3), perhaps due to its lipophilicity and hence, ability to gain access to the micellar inner cores. Thus, although KO*t*Bu in water leads to mainly KOH, an equilibrium (small but effective) percentage is localized inside the micelle, where substrates and catalysts are positioned at high concentrations.^69^ Alternatively, a combination of KOH and *t*BuOH, resulting in KO*t*Bu being formed in situ, was also studied, leading to **1** in 90% isolated yield (see Supporting Information, Table S3, entry 4).

As part of the screening process, the surfactant forming the basis of the micellar aqueous reaction medium was explored. A series of amphiphiles was evaluated in terms of the nanoreactors formed in situ for these aminations (Table 2). Under otherwise identical conditions using the same 2 wt % of each in water, a broad range of yields of **1** was obtained. The recently introduced, more polar, and biodegradable Savie gave the best result (entry 7; 99%) as compared to other nonionic surfactants (entries 2–5). The corresponding background reaction “on water”^75^ (entry 1) gave rise to the desired product in a modest 63% yield. While other nonionic surfactants were lower yielding, Kolliphor ES (entry 4) was competitive, perhaps due to its known avoidance of solubilization of oxygen, as shown previously by Beverina.^76^ Attempts to reduce catalyst loading (e.g., to 750 ppm or 0.0750 mol % dimer, which is 0.15 mol % total Pd) of [Pd(crotyl)Cl]_2_ led to a marked decrease in the yields of **1** (see Supporting Information, Table S6). Furthermore, reducing the ligand loading to 1 mol % resulted in a significant reduction in the yield of **1** from 99 to 70% (see Supporting Information, Table S7).

**Table 2:**
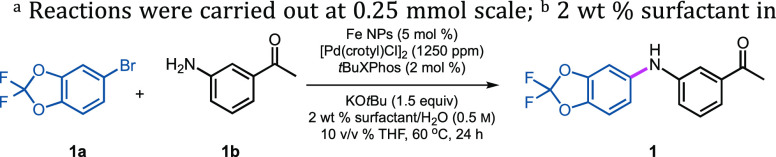
Surfactant Screening

entry^a^	surfactant^b^	yield (%)^c^
1	pure water	63
2	PTS-600	66
3	TPGS-750-M	75
4	Kolliphor	91
5	Triton-X	31
6	MC-1	85
7	**Savie**	**99**

a Reactions were carried out at 0.25 mmol scale.

b 2 wt % surfactant in water, 0.5 M global concentration.

c NMR yields using 1,3,5-trimethoxybenzene as the internal standard.

Additional control experiments between **1a** and **1b** leading to arylamine **1** document the essential roles played by all components involved in these couplings: the Pd, the ligand, and the Fe NPs (Table 3). Optimized conditions for NP-catalyzed aminations of aryl bromides, therefore, were thus determined to be Fe NPs (5 mol %), [Pd(crotyl)Cl]_2_ (1250 ppm, 0.125 mol % of this dimeric species) as the Pd precursor, and *t*BuXPhos (2 mol %) as ligand in 2 wt % Savie/H_2_O, containing KO*t*Bu (1.5 equiv) as base at 60 °C.

**Table 3:**
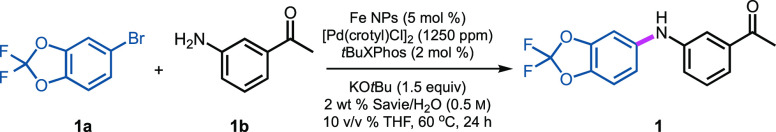
Variations from Standard Conditions

entry^a^	deviations from standard conditions	yield (%)^b^
**1**	**none**	**99 (91)**^c^
2	45 °C instead of 60 °C	81
3	1 mol % ligand	70
4	[Pd(crotyl)Cl]_2_ (750 ppm)	45
5	no Fe NPs	60
6	Fe NPs, no [Pd]	d
7	Fe NPs, no ligand	trace

a Reactions were carried out at 0.25 mmol scale.

b NMR yields using 1,3,5-trimethoxybenzene as the internal standard.

c Isolated yield in parentheses.

d No product observed.

### NP Characterization

**Figure 1:**
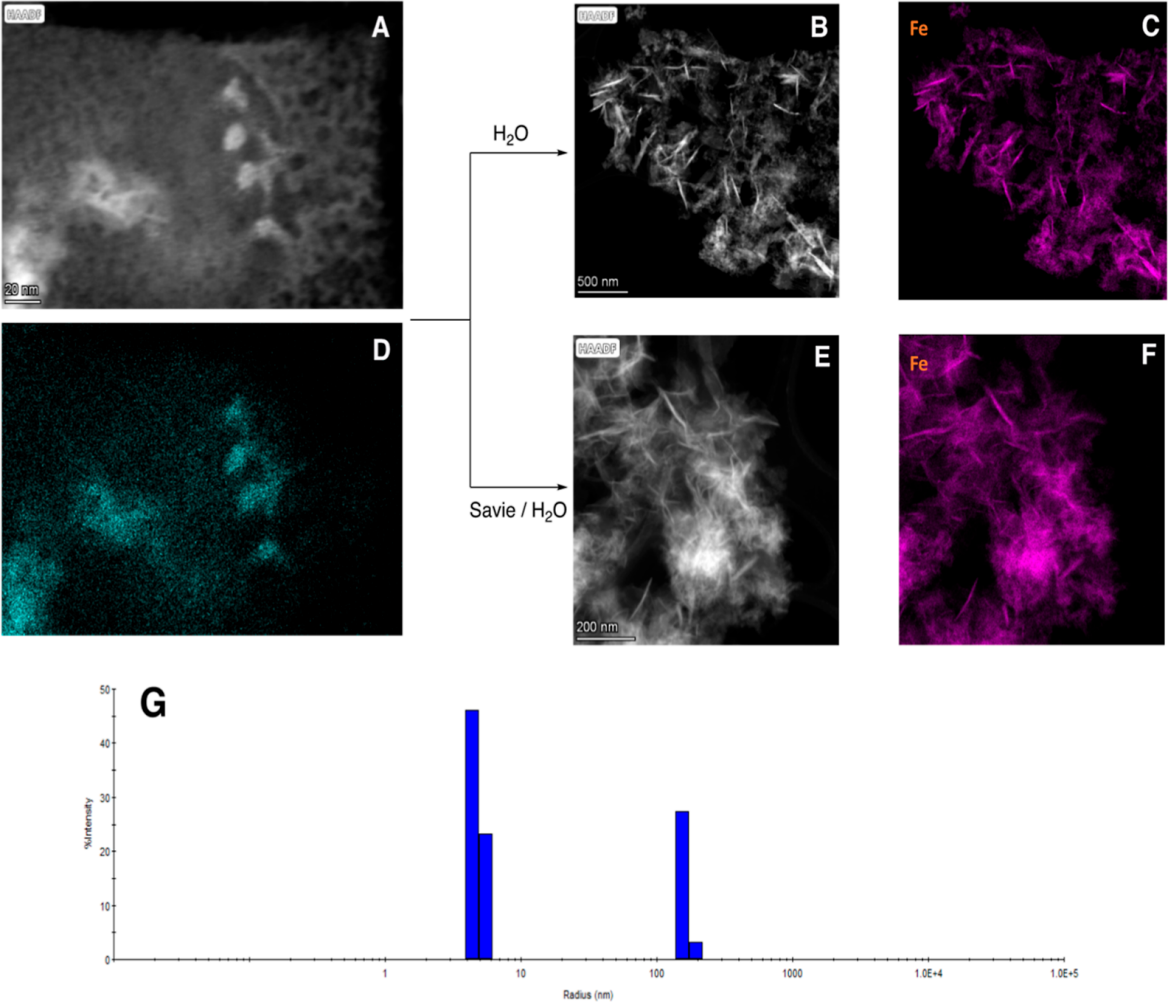
(A) High-angle annular dark-field imaging STEM image of Fe NPs dry powder; (B) high-angle annular dark-field imaging STEM image of Fe NPs dry powder in degassed water; (C) Fe elementary mapping of NPs dry powder in degassed water; (D) Fe elementary mapping of NPs dry powder; (E) high-angle annular dark-field imaging STEM image of Fe NPs dry powder in 2 wt % Savie/H_2_O; (F) Fe elementary mapping of NPs dry powder in 2 wt % Savie/H_2_O; and (G) DLS analysis of Fe NPs in 2 wt % Savie/H_2_O solution.

The nature of these NPs was next investigated. With scanning transmission electron microscopy–energy dispersive X-ray spectroscopy (STEM–EDX) imaging, the spherical shape of the initially formed Fe NP dry powder could be seen, measuring 5–10 nm (Figure 1A).

In aqueous solution, these spheres undergo the “sculpting process”, i.e., a morphology change to rod-like structures (ca. 100–400 nm; Figure 1B).^55^ When the same Fe NPs were subjected to 2 wt % Savie in water, more evenly distributed, smaller needle-like nanorods (ca. 100–200 nm) were observed (Figure 1E). Elemental mapping of Fe (Figure 1C,D,F) confirmed the composition of the nanoparticles. Similar mapping and EDX analysis (see Supporting Information, Section S4) also revealed the presence of Mg, Fe, and Cl within these nanorod catalysts, as expected. Dynamic light scattering (DLS) indicated the size of the nanomicelles and nanoparticles (Figure 1G; see also Supporting Information, Section S4). The NPs in an aqueous solution containing Savie show the expected two peaks, one at 5–6 nm associated with the nanomicelles, while the other at 158 nm is indicative of nanoparticle aggregates. These data match those from previous observations.^55,56^ Moreover, studies by Hou and co-workers have shown that polyethers can stabilize metal-containing nanoparticles and prevent Ostwald ripening via favorable interactions between ethereal oxygen atoms and the metal, in essence, functioning as a ligand.^77^ This may explain the observation that nanomicelles consisting of oxygen-rich PEG deliver their “payload” (i.e., the substrates contained therein) to the nanoparticle catalyst (hence, “nano to nano”), thereby necessitating only mild conditions for the intended catalysis.^53^ This association is apparent from the agglomeration of TPGS-750-M-derived nanomicelles localized around the catalyst nanorods.^78^ Identical findings were also noted for nanomicelles associated with the recently reported surfactant Savie, composed of polysarcosine (PSar) in place of PEG.^50^ Thus, for these newly ligated NPs that affect aminations, TEM imaging confirms that Savie-derived nanomicelles participate in this effect, which is solely observed in aqueous reaction media (Figure 1).

### Scope of C–N Cross-Couplings

**Scheme 2:**
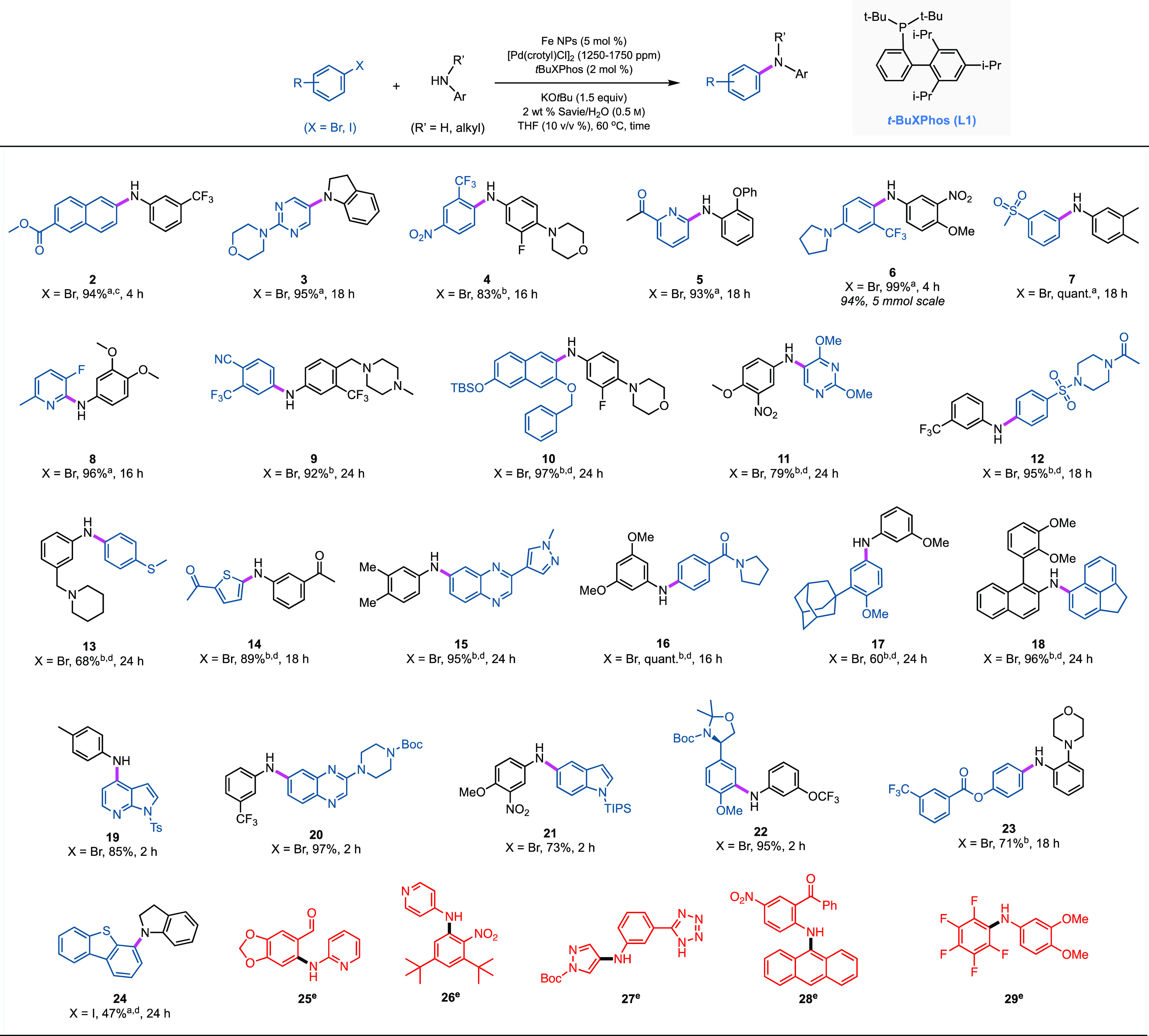
Scope of Aromatic Amines Used in Aminations Unless otherwise mentioned: aryl bromide (0.25 mmol), ArNHR’ (0.375 mmol), [Pd(crotyl)Cl]_2_ (0.125 mol %, 0.25 mol % Pd), *t*BuXPhos (2 mol %), KO*t*Bu (0.375 mmol), 2 wt % Savie/H_2_O (0.45 mL), THF (0.05 mL), 60 °C; ^b^[Pd(crotyl)Cl]_2_ (0.175 mol %, 0.35 mol % Pd); ^c^45 °C; ^d^70 °C; ^e^attempted couplings that were unsuccessful.

The use of Fe NPs containing *t*BuXPhos as ligands catalyzed a wide variety of heterogeneous couplings between functionalized aryl bromides and anilines. A low catalyst loading (2500–3500 ppm, or 0.25–0.35 mol % [Pd]; 0.125–0.175 mol % dimer), along with modest temperatures and reaction times (60 °C for typically 16–24 h), led to high isolated yields of aminated products (Scheme 2). Substrates with base-sensitive functionality (e.g., ester) were well tolerated, although these reactions were best run at lower temperatures (45 °C) and for shorter reaction times (4 h), affording the desired coupled amine in high yield (e.g., product **2**). Aryl bromides or anilines containing acidic protons (e.g., the precursors to product **5**) demonstrated excellent selectivity toward amination rather than competitive α-arylation, which also employs KO*t*Bu as the base.^79^ Highly functionalized educts containing electrophilic groups such as nitrile (e.g., see product **9**) underwent coupling likewise very efficiently. As the extent of functionality in each partner increased, the loading of catalyst also required a slight increase to 1750 ppm (0.175 mol % of the dimeric species [Pd(crotyl)Cl]_2_), in addition to an increase in reaction temperature (70 °C). Notwithstanding these somewhat more aggressive conditions, the desired C–N bond constructions could be carried out smoothly, conditions that would be made all the more attractive by subsequent recycling, which is yet another unprecedented aspect of this chemistry (vide infra).

Several *N*-heterocycle-containing aryl bromides and anilines, which often present added challenges in transition metal-catalyzed coupling reactions due to their propensity to coordinate with the catalyst,^80^ can also be found among the coupled products formed in good-to-excellent yields (see products **3, 8, 11, 12, 15, 19**, and **20** in Scheme 2). Structurally diverse aryl bromides, halogenated at various positions, were coupled effectively irrespective of the nature of the functional groups on the ring (i.e., being electron neutral, donating, or withdrawing). It is worthy of note that *ortho*-substituted aromatic bromides and amines couple without incident, despite steric hindrance (e.g., see products **10**, **18**, and **22**). Amination leading to product **13** in only modest yield was noticeably slow, probably due to the coordination of Pd with sulfur. A number of substrate combinations were found to be low-yielding or incompatible with these C–N bond formations in water. One type of coupling along these lines includes aryl iodides, known to be relatively poor participants resulting from catalyst-altering effects of the iodide ion,^81,82^ which gave in the case of product **24** only 47% yield. When highly electron-deficient anilines, such as 2-aminopyridine, 4-aminopyridine, and 2-amino-5-nitrobenzophenone were selected as reaction partners, only trace amounts of coupled products **25**, **26**, and **28** were observed. Additionally, attempts to couple aryl bromides with 5-membered heterocyclic amines (e.g., a pyrazole, as in **27**) led to substrate decomposition, as is typically observed in the presence of strong alkoxide bases.^36^ Finally, the use of a polyfluorinated aryl bromide led to a mixture of products rather than amine **29**, reflecting likely competing S_N_Ar reactions at various sites.

### C–N Bond Formation in Ocean Water

**Scheme 3:**
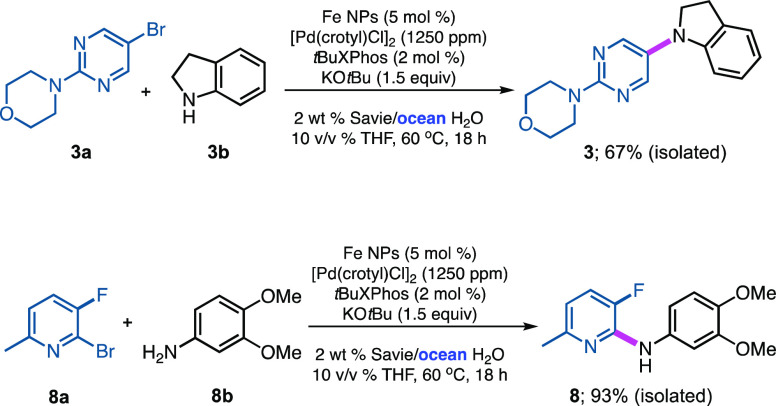
Comparisons of Reactions Run in Ocean Water

The presence and impact of salts in aqueous micellar media have previously been evaluated in terms of their effects on various Pd-catalyzed C–C bond-forming reactions.^83^ In addition to prior art, the potential use of ocean water as an alternative medium minimizes the cost associated with this reaction variable. Moreover, the use of seawater for Pd-catalyzed aminations, while done heterogeneously, had never been examined. Hence, two reactions of this type were conducted as part of this overall study (Scheme 3; see Supporting Information Section 3.2). With aromatic amine **3b**, the switch to seawater was found to have a noticeably deleterious effect on the reaction rate, as the extent of conversion and eventual isolated yield dropped from 95% (see Scheme 2) to 67% under otherwise identical conditions, although optimization was not carried out. However, with substituted aniline **8b**, conversion to product **8** was roughly comparable (93%) to the result obtained using HPLC-grade water (96%), with the coupling being given the same 16 h reaction time. Thus, from this brief investigation, it can be concluded that prospects for aminations in seawater, in general, look encouraging in place of purified water.

### Recycling Studies

**Scheme 4:**
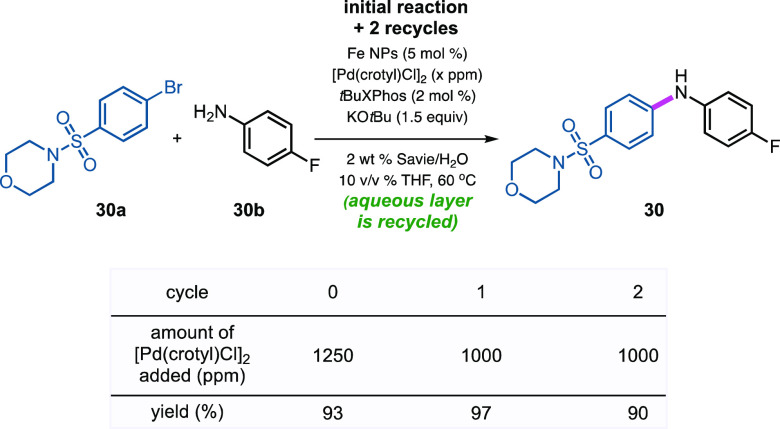
Recycling Studies

An often-used metric as a quick indication of the level of “greenness” associated with a reaction is Sheldon’s *E* Factor.^84−86^ Recycling of the aqueous reaction mixture can dramatically alter the *E* Factor,^85,86^ which is a major benefit associated with chemistry in water. Thus, following an initial reaction between 4-((4-bromophenyl)sulfonyl)morpholine **30a** and 4-fluoroaniline **30b** (Scheme 4), recovery of product **30** can be accomplished using an in-flask extraction with recyclable amounts of EtOAc (see Supporting Information, Section S5). Likewise, reuse of the aqueous phase remaining in the original reaction vessel for two additional cycles led to excellent yields of aminated product **30**. Overall, these three reactions required a total investment of only 0.65 mol % Pd, or 0.21 mol % per amination. After the third reaction (second recycle), salt buildup increased viscosity to the point where additional use of the aqueous reaction mixture was precluded. *E* Factors associated with this recycling were 2.1 (when recyclable EtOAc is not considered waste; see Supporting Information Section S5) and 14.5 (when EtOAc is considered waste; see Supporting Information Section S5). These compare very favorably with typical values associated with the pharmaceutical industry that vary, according to Sheldon,^84−86^ between 25 and 100, *without the inclusion of water in the calculation.* Perhaps equally importantly, ICP–MS analyses of product **30**, *after standard workup and purification*, showed residual metal levels of 1.56 ppm Pd, unlike levels to be expected for aminations run with far higher loadings of Pd in organic solvents.^37^ Since these values are well below those allowed by the FDA of 10 ppm Pd per dose per day,^62^ no additional processing to remove residual Pd is needed, which can otherwise be costly and time-consuming.

### Late-stage C–N Cross Couplings with Complex, Pharmaceutically Relevant Substrates

**Scheme 5:**
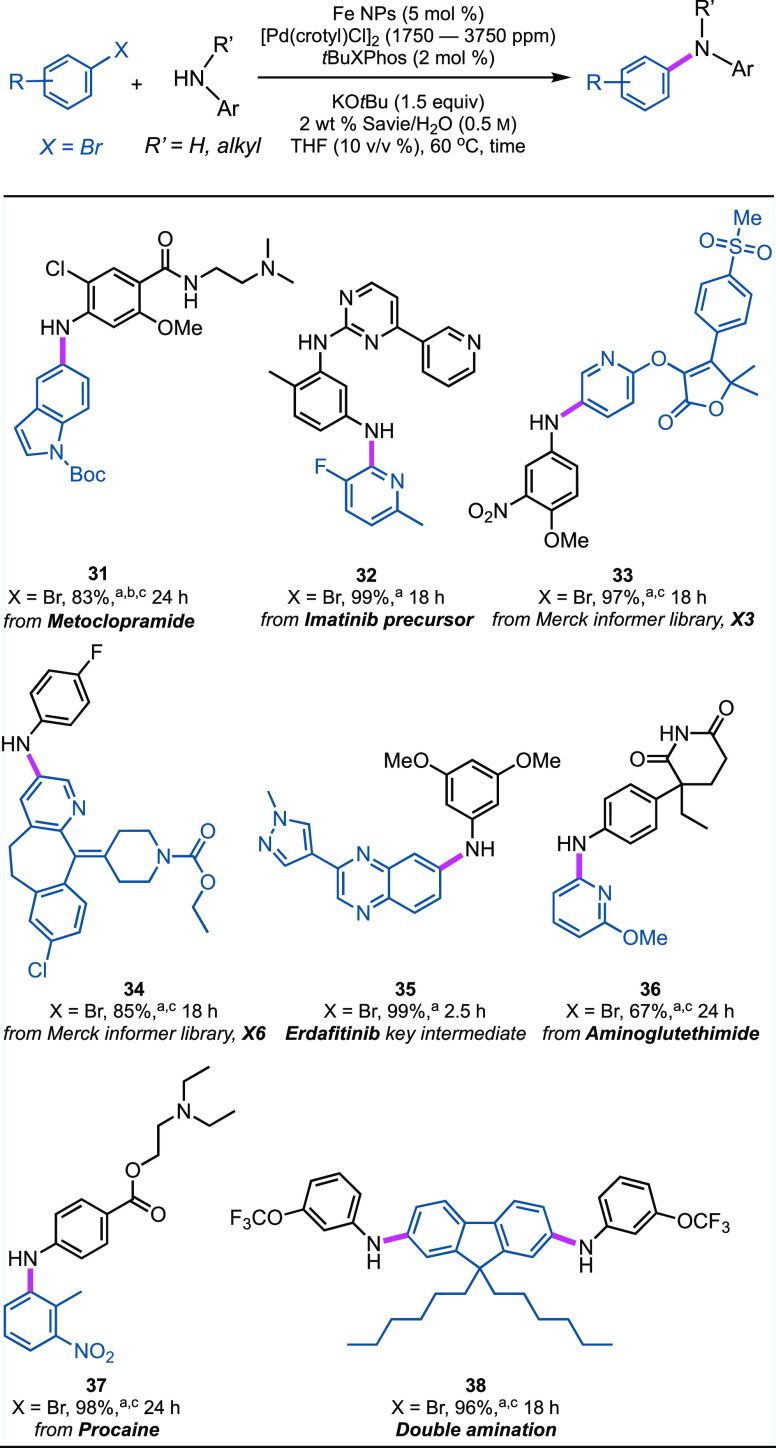
Representative Late-Stage Aminations Aryl bromide (0.25 mmol), ArNHR’ (0.375 mmol), Fe NPs (5 mol %), [Pd(crotyl)Cl]_2_ (0.125 mol %, 0.25 mol % Pd), *t*BuXPhos (2 mol %), KO*t*Bu (0.375 mmol), 2 wt % Savie/H_2_O (0.45 mL), THF (0.05 mL), 60 °C; ^b^HCl salt of the amine was used; ^c^[Pd(crotyl)Cl]_2_ (0.175 mol %, 0.35 mol % Pd).

C–N Bond formations involving late-stage pharmaceutical derivatives bearing multiple functional groups are known to exhibit a high rate of failure,^87,88^ occasionally requiring the use of *stoichiometric levels of palladium*, as reported by Buchwald.^89^ Several structurally complex pharmaceuticals bearing amines were obtained following coupling of aryl or heteroaryl bromides, including examples from the Merck Informer Library (Scheme 5).^87^ Notwithstanding the use of highly functionalized educts, excellent results were obtained for several noteworthy cases, including (a) the antiemetic and gut motility stimulator metoclopramide with *N*-Boc-5-bromoindole affording **31**; and (b) the arylation of a pyridine- and pyrimidine-containing polycyclic aniline, a reaction partner en route to the antileukemia agent Imatinib (Gleevec) affording product **32**. Only 3500 ppm (0.35 mol %) of Pd catalyst (0.175 mol % of the dimer) was needed. The coupling of densely functionalized aryl bromides from the Merck Informer Library^87^ also proceeded smoothly for products **33** and **34**. Key intermediates en route to the anticancer drug Erdafitinib (**35**; Balversa) were realized in excellent yield. Likewise, arylation of (i) aminoglutethimide (Elipten), leading to product **36**, which is used in the treatment of seizures, Cushing’s syndrome, and breast and prostate cancer; (ii) procaine (**37**; Novocain), a local anesthetic; and (iii) double amination of a dibromo-fluorene (giving **38**), which acts as a photoluminescent probe that can be incorporated into LEDs,^90^ all proceeded efficiently. It is noteworthy that the glutarimide moiety in aminoglutethimide (product **36**) does not fragment under these strongly basic conditions, thereby selectively affording the targeted product of amination. Collectively, C–N couplings of this nature involving complex pharmaceuticals and materials used under environmentally responsible heterogeneous conditions are particularly timely and further establish the generality of these technologies as important tools in the growing toolbox that are based on chemistry in water.

### Direct Comparisons with Recent Literature

**Scheme 6:**
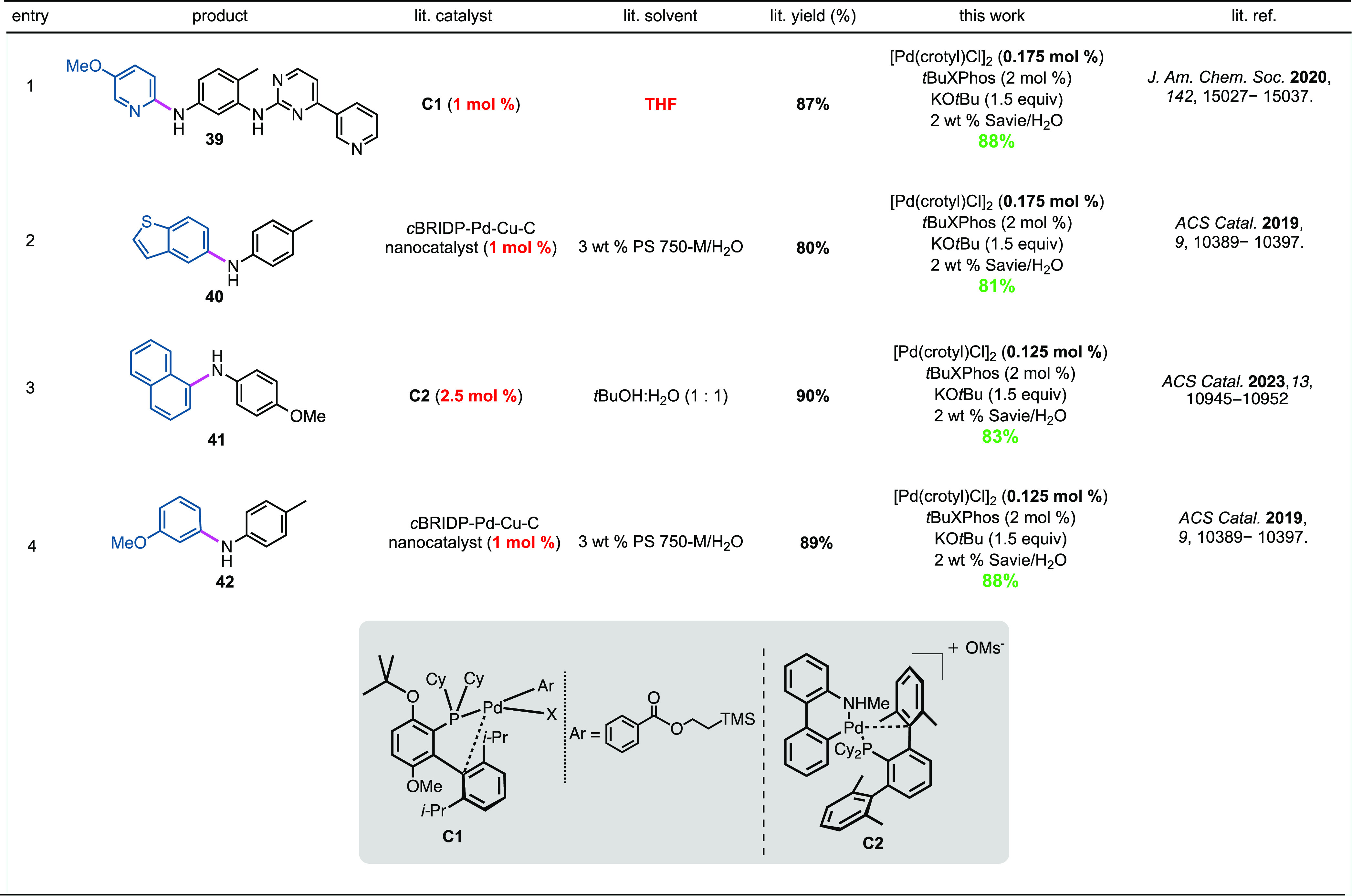
Comparison between Fe/ppm Pd NP s and Representative Recent Examples of C–N Bond Constructions

Illustrated in Scheme 6 are several comparison cases focused on existing literature approaches to C–N bond formation.^35,69,91^ Aminations arriving at products **39**–**42** indicate that the catalytic system described here based on [Pd(crotyl)Cl]_2_–*t*BuXPhos, in general, appears to be the most effective known. It allows for aminations at lower catalyst loadings of metal, takes place in predominantly aqueous micellar media, and leads to typically faster couplings than the corresponding reactions in organic solvents. Moreover, yields tend to be comparable to, if not higher than, those reported previously. From the perspective of sheer convenience, leaving aside the obvious environmental differences, the commercial availability of the Pd dimer and associated ligands that avoid pre-catalyst formation^92^ suggests that this new heterogeneous catalytic system based on NPs offers several advantages to practitioners previously unavailable.

### Aminations Using Pseudohalides

**Scheme 7:**
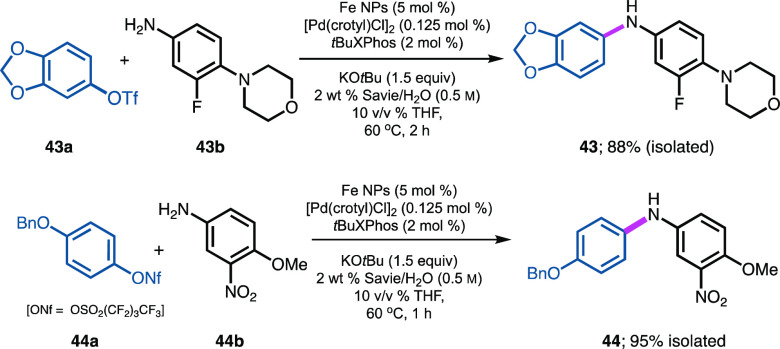
Representative Examples of Pseudohalides Used for Pd-catalyzed C–N Bond Formation

Pseudohalides have been extensively investigated over time as an alternative to halide-leaving groups in Pd-catalyzed C–N coupling reactions.^93−95^ As shown in Scheme 7, both triflate and nonaflate derivatives of the corresponding phenols are amenable. Thus, from triflate **43a** and amine **43b**, product **43** could be obtained in 88% isolated yield. Product **44** was isolated in 95% yield using coupling partners **44a**, a nonaflate, together with aniline **44b**. Base-promoted cleavage of either educt^9^ was not observed under these aqueous conditions.

### Representative 1-pot, 5-step Sequence

**Scheme 8:**
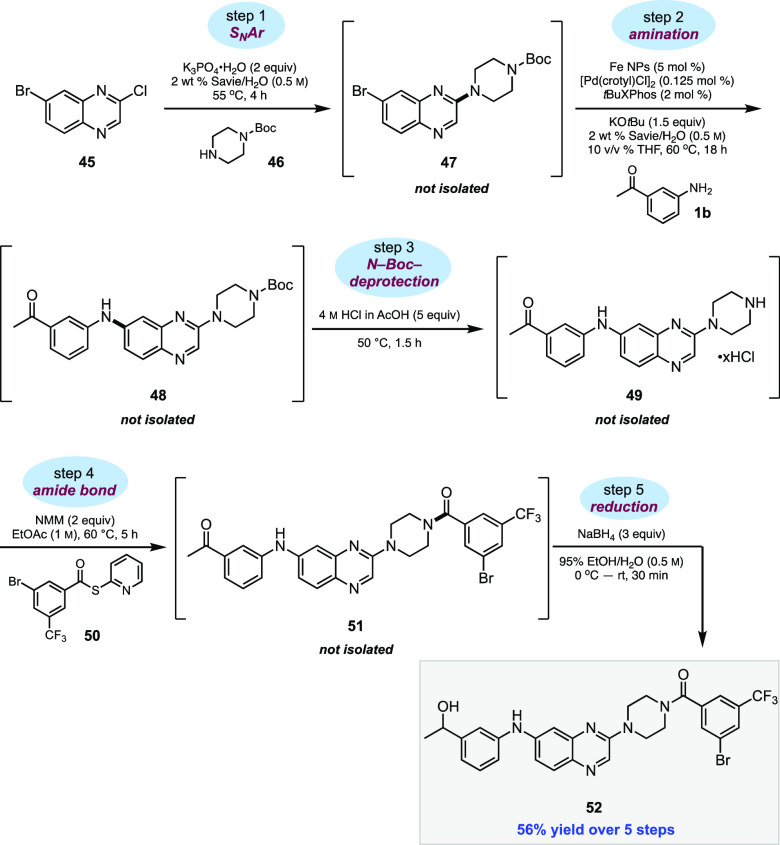
1-pot, 5-step Sequence in Water Demonstrating Pharmaceutically Important Reactions

While the number of reactions that can be run under micellar conditions continues to expand,^96^ so have the advantages of using sequences in water, leading to both “pot”^97,98^ and “time”^99,100^ economies. These benefits, among others (e.g., minimizing waste creation), are the subject of both recent reports and reviews.^97−100^ In Scheme 8, a 1-pot, 5-step sequence is not only illustrative of chemistry in water but, by contrast, is otherwise unknown involving traditional aminations given the requirements for each reaction type to be carried out in a different organic solvent.^37^ The series shown includes some of the most widely used transformations in the pharmaceutical industry.^19^

Hence, an initial S_N_Ar reaction in water between quinoxaline **45** and *N*-Boc-protected piperazine **46** takes place exclusively at the carbon bearing the chloride to afford intermediate **47**. The resulting crude aqueous mixture is then subjected to amination in the same pot using 3-aminoacetophenone (**1b**) to afford intermediate **48**. Moreover, while excess (1.5 equiv) *N*-Boc piperazine was used in the previous step, nonetheless, complete selectivity in this Pd-catalyzed amination involving aniline **1b** was observed. Again, and without isolation, *N*-Boc deprotection (see Supporting Information, Section S6 for optimization) of **48** leads to HCl salt **49**, which readily participates in amide bond formation *in the same reaction vessel* with in situ-formed thioester **50**(102) to afford amide **51**. This densely functionalized material (**51**) can then be reduced in step 5 to the corresponding alcohol (NaBH_4_) in green and inexpensive 95% EtOH to afford benzylic alcohol **52** in a 56% overall yield. ICP–MS analysis for residual palladium in **52** indicated that only 0.6 ppm was present, which, as noted above, is well below the FDA-approved limit of 10 ppm Pd/dose/day.^62^

## Conclusions

In summary, a newly developed, environmentally responsible, and previously unknown technology for *heterogeneous* Pd-catalyzed aminations of aryl halides and pseudohalides in recyclable water is reported that relies on sustainable loadings of precious metal. The advances disclosed herein include:

- the application of iron nanoparticles (Fe NPs) for C–N bond constructions;
- commercially available catalyst precursors;
- the first application of the amphiphile Savie as a biodegradable surfactant used in recyclable aqueous media;
- use of recyclable Pd at low levels, thereby providing rare examples of “metal economy”;
- use of ocean water rather than fresh water as reaction medium;
- the successful application to highly functionalized, complex targets, including pharmaceuticals and related species;
- use in a multistep sequence not possible in a single organic solvent, leading to both time and pot economies.

## Supporting Information Available

Experimental procedures, optimization details, and analytical data of isolated materials (NMR and HRMS) (PDF)
