# Supplementary material for: Nanoparticles as Heterogeneous Catalysts for ppm Pd-Catalyzed Aminations in Water
Source: ACS Sustain Chem Eng. 2024 Jan 22;12(5):1997–2008. doi: 10.1021/acssuschemeng.3c06527 (PMC10848299; doi:10.1021/acssuschemeng.3c06527)
Supplement: Supplementary file 1 — sc3c06527_si_001.pdf [file sc3c06527_si_001.pdf]

Supporting Information

Nanoparticles as *heterogeneous* catalysts for ppm Pd–catalyzed aminations *in water*

Karthik Iyer, Rahul Kavthe, Yuting Hu, and Bruce H. Lipshutz*
Department of Chemistry and Biochemistry, University of California, Santa Barbara, CA 93106 USA

Phone: 805–893–2521
Fax: 805–893–8265
Email: lipshutz@chem.ucsb.edu
Website: https://lipshutz.chem.ucsb.edu/

Table of Contents

1. General Information S3
2. General procedure for the preparation of iron nanoparticles (Fe NPs): S5
2.1. Titration of MeMgCl in THF with $LiCl/I_2$: S5
3. Optimization of reaction conditions S5
3.1. Optimization for coupling of aryl halides with aromatic amines S5
3.1.1. Ligand screening for coupling of aryl halides with aromatic amines S6
3.1.2. Screening of palladium sources S9
3.1.3. Screening of bases S10
3.1.4. Surfactant screening S11
3.1.5. Screening of amine equivalents S11
3.1.6. Catalyst loading screen S12
3.1.7. Ligand loading screen S12
3.1.8. Temperature screening S13
3.1.9. Additional control experiments S13
3.1.10. General procedure for coupling of aryl halides with anilines using Fe NPs: Procedure 1 .... S14
3.2. General procedure for aminations using ocean water: Procedure 2 .... S15
3.2.1. Preparation of a 2 wt % solution of Savie in ocean water S15
3.2.2. Procedure for aminations using ocean water S15
4. Analyses of nanoparticles (NPs) S16
4.1. STEM–EDS analysis S16
4.2. Dynamic Light scattering (DLS) analysis S21
5. Recycling studies and E Factor calculations S23
5.1. Recycling studies S23
5.2. E Factor calculations S25
6. General procedure for gram–scale synthesis of compound 6 – Procedure 3 .... S26
7. 5–Step, one–pot sequence to afford compound 52 .... S27

7.1. Optimization of *N*–Boc deprotection on model substrate 47 .... S27
7.2. General procedure for the synthesis of thioester 50: Procedure 4 .... S28
7.3. General procedure for the 5–step, 1–pot sequence to afford 52: Procedure 5 .... S28
8. ICP–MS analysis for residual palladium S30
9. References S31
10. Analytical data S31
10.1. Analytical data for starting materials and intermediates S31
10.2. Analytical data of coupled products S33
11. NMR spectra of intermediates and products S59

number of pages: 120
number of Tables: 12
number of Figures: 16
number of Schemes: 6

1. General Information

Safety Statement:

No unexpected or unusual safety hazards were encountered. However, areas where extra safety precautions were taken have been explicitly noted.

Reagents:

All commercially available reagents were used without further purification with the exception of N–Boc–Sar (Sar = sarcosine) which was purified by hot filtration with EtOAc or *i*–PrOAc followed by recrystallization from hot EtOAc or *i*–PrOAc. Reagents were purchased from Sigma–Aldrich, Combi–Blocks, Alfa Aesar, Acros Organics, A2B chemicals, BLD Pharma, or AK Scientific.

Surfactant Solution Preparation:

A 2 wt % TPGS–750–M / H_2O solution was prepared by dissolving TPGS–750–M in degassed HPLC grade water; likewise, 2 wt % aqueous solutions of Savie, Kolliphor ES, Triton X, MC–1 and PTS 600 were prepared in the same manner. TPGS–750–M was made as described previously[1] and is also commercially available from Sigma–Aldrich (catalog #733857 (solution) or #763896 (wax)). Savie was prepared as described previously[2] and will soon be available from Sigma–Aldrich (catalog #926981) and in larger quantities from PHT International. HPLC–grade water was obtained from Sigma Aldrich and Fischer Scientific and was purged with argon before use. Seawater was obtained from the Pacific Ocean, from the beach associated with UCSB.

Chromatography:

Silica gel TLC plates (UV 254 indicator, thickness 200 mm standard grade, glass backed and 230–400 mesh from Merck, and Silicycle) were used. The developed TLC plate was analyzed with a UV lamp (254 nm). The plates were further analyzed with use of an aqueous ceric ammonium molybdate stain, potassium permanganate stain, or ethanolic vanillin and developed with a heat gun. Flash chromatography was performed using Silicycle Silicaflash® P60 unbonded grade silica.

Nuclear Magnetic Resonance Spectroscopy (NMR):

^{1}H, ^{13}C, and ^{19}F NMR were recorded at 25 ºC on an Agilent Technologies 400 MHz, a Bruker Avance III HD 400 MHz, a Bruker Avance NEO 500 MHz, a Varian Unity Inova 500 MHz, or a Varian Unity Inova 600 MHz spectrometer in $CDCl_3$ or DMSO–d_6 with residual $CHCl_3$ (^{1}H = 7.26 ppm, ^{13}C = 77.16 ppm) or DMSO (^{1}H = 2.54 ppm, ^{13}C = 40.45 ppm) as the internal standard. Deuterated solvents were purchased from Cambridge Isotope Laboratories. Chemical shifts are reported in parts per million (ppm). The data

presented will be reported as follows; chemical shift, multiplicity (s = singlet, bs = broad singlet, d = doublet, dd = doublet of doublet, t = triplet, q = quartet, quin = quintet, m = multiplet), coupling constant (if applicable), and integration.

Mass Spectrometry (MS):

HRMS analyses (ESI–MS, CI–MS, or GC–EI) were performed by the UC Santa Barbara mass spectrometry facility or the UC Irvine mass spectrometry facility. ICP–MS analysis was performed at the California NanoSystems Institute (CNSI), UCLA. ESI–MS analysis was performed on a Waters LCT Premier mass spectrometer equipped with an Alliance 2695 Separations module. EI–MS analysis was performed on a Waters GCT Premier mass spectrometer equipped with an Agilent 7890A GC oven and J&W Scientific DB–5ms+DG narrow bore column using helium carrier gas.

Inductively coupled plasma mass spectrometry (ICP–MS, NexION 2000, PerkinElmer) analysis was performed to detect palladium in powder samples. All samples were used as received without further purification or modification. Each sample transferred to clean Teflon vessel for acid digestion. Digestion was carried out with a mixture of concentrated HNO_3 (65–70%, Trace Metal Grade, Fisher Scientific) and HCl (35–38%, Trace Metal Grade, Fisher Scientific) in a ratio of 1:3 with a supplement of H_2O_2 (30%, Certified ACS, Fisher Scientific) at 200 °C for 50 min in a microwave digestion system (Titan MPS, PerkinElmer). Once the sample was cooled to rt, it was subsequently diluted to make a final volume of 50 mL by adding filtered DI water for analysis. The calibration curve was established using a standard solution while the dwell time was 50 ms with thirty sweeps and three replicates with background correction.

Dynamic Light Scattering (DLS):

DLS data was collected using a DynaPro NanoStar™ from Wyatt Technology equipped with a He–Ne, 4 mW, 658 nm red laser.

Transmission Electron Microscopy (TEM):

TEM experiments were conducted at the Microscopy and Microanalysis Facility, UC Santa Barbara. Briefly, a 3 μL sample was applied to a 400–mesh ultra–thin carbon grid and incubated for 1 min. The grid was subsequently blotted dry by a piece of filter paper. The TEM grid was imaged in a ThermoFisher Talos G2 200X TEM/STEM w/ChemiSTEM EDS.

2. General procedure for the preparation of iron nanoparticles (Fe NPs):

In an oven–dried 25 mL round bottom flask purged with argon, covered with a rubber septum containing a PTFE–coated magnetic stir bar, $FeCl_3$ (250 mg, 1.54 mmol) and anhydrous THF (4 mL) were added under a stream of dry argon. The mixture was stirred for 15 min. While maintaining a dry atmosphere at rt, a 1 M solution of MeMgCl in THF (3.1 mL) was very slowly (1 drop/2 sec) added to the reaction mixture (alternatively, a syringe pump could also be used to facilitate addition). After complete addition of the Grignard reagent, the mixture was stirred for an additional 15 min at rt. THF was removed *in–vacuo*, and the solid–state Fe NPs were transferred and stored in glove box.

2.1. Titration of MeMgCl in THF with LiCl/I_2:

To an oven dried 25 mL round bottom flask, anhydrous LiCl (424 mg, 10 mmol) was added under an argon atmosphere in the glovebox. The flask was sealed with a rubber septum, and 20 mL anhydrous THF was added by syringe and the mixture was stirred at rt until the LiCl was completely dissolved, resulting in the formation of a 0.5 M solution of LiCl in THF. A 10 mL microwave vial equipped with a magnetic stirring bar and a septum was heated with a heat gun under reduced pressure and cooled to rt under an argon atmosphere. In the glovebox, the dry microwave vial was charged with accurately weighed I_2 (127 mg, 0.5 mmol) and capped with a rubber septum. The saturated solution of LiCl in THF (2 mL) was added and stirring was started. After the iodine was completely dissolved, the resulting brown solution was cooled to 0 °C in an ice bath. Another 5 mL round bottom flask equipped with a magnetic stirring bar and a septum was heated with a heat gun under reduced pressure and cooled to rt under an argon atmosphere. To this round bottom flask, MeMgCl solution (from Sigma–Aldrich, catalog No. 189901; 3 mL) and anhydrous THF (6 mL) were added by syringe and then stirred. To the vial with I_2, the MeMgCl solution was added dropwise via a 1.0 mL syringe (0.01 mL graduation) until the brown color disappeared. The amount consumed contains 1 equiv of the MeMgCl relative to iodine. The MeMgCl solution was titrated five times.

3. Optimization of reaction conditions

3.1. Optimization for coupling of aryl halides with aromatic amines

Preparation of a stock solution of $Pd(OAc)_2$ in THF:

To a 1–dram vial with a PTFE coated magnetic stir–bar was added $Pd(OAc)_2$ (2.8 mg, 0.0124 mmol). The vial was sealed with a rubber septum and evacuated and backfilled with argon three times using an

argon/vacuum manifold. This was followed by the addition of anhydrous THF (obtained from a solvent purification system; 1 mL). The vial was stirred gently at rt until the $Pd(OAc)_2$ dissolved completely. For a 0.25 mmol scale reaction, 50 µL of the stock solution would contain 2500 ppm (0.25 mol %) of Pd, which was used directly for further optimization.

3.1.1. Ligand screening for coupling of aryl halides with aromatic amines

Reaction setup: To a 1–dram vial equipped with a PTFE coated magnetic stir bar was added 3–Aminoacetophenone **1b** (1.5 equiv, 0.375 mmol, 51 mg). The vial was sealed with a rubber septum, evacuated, and backfilled with argon three times using an argon/vacuum manifold and then taken into an argon filled glovebox, where Fe NPs (10 mg, 5 mol %), the specified ligand (2 mol %), and KO*t*Bu (1.5 equiv, 42 mg) were added. Subsequently, under an atmosphere of argon, 5–bromo–2,2–difluorobenzo[d][1,3]dioxole **1a** (1 equiv, 0.25 mmol, 59.2 mg, 34 µL) was added followed by the addition of a 2 wt % Savie / H_2O (0.45 mL) solution and the vial was allowed to stir at rt for 1–2 min. Finally, the stock solution of $Pd(OAc)_2$ in THF (50 µL, 2500 ppm, 0.25 mol % Pd) was added and the reaction was allowed to stir at 60 °C for 24 h. Upon completion (as monitored by TLC), the reaction was extracted with EtOAc (4 x 1 mL). The combined extracts were dried over anhydrous Na_2SO_4, filtered, and concentrated in vacuo. Subsequently, 2 mL of $CDCl_3$ was added followed by the addition of 1,3,5–trimethoxybenzene as internal standard and the sample was analyzed by ^1H NMR (10 s relaxation delay).

Table S1. Screening of ligands.

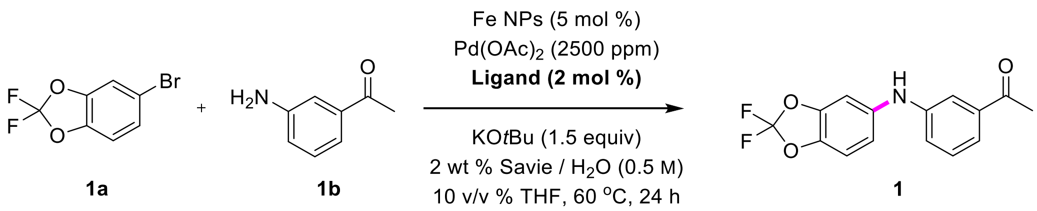

entry[a]	ligand	yield (%)[b]
1	XantPhos	10
2	Dppf	0
3	SPhos	0
4	cBRIDP	trace
5	*t*BuBrettPhos	20
**6**	***t*BuXPhos**	**90**
7	rac–BINAP	20
8	P^tBu_3	17
9	$Fc(PAd)_2$	0
10	BIDIME	0
11	$Fc(P^tBu_2)(PAd_2)$	0
12	QPhos	10
13	N_2Phos	0
14	XPhos	23
15	Cy cBRIDP	0
16	PAd_3	13
17	P(*p*–Tol)$_3$	0
18	AdBrettPhos	38
19	MorDalPhos	0

[a] Reactions were carried out on a 0.25 mmol scale; [b] ^{1}H NMR yields using 1,3,5 –trimethoxybenzene as internal standard.

Structures of Ligands

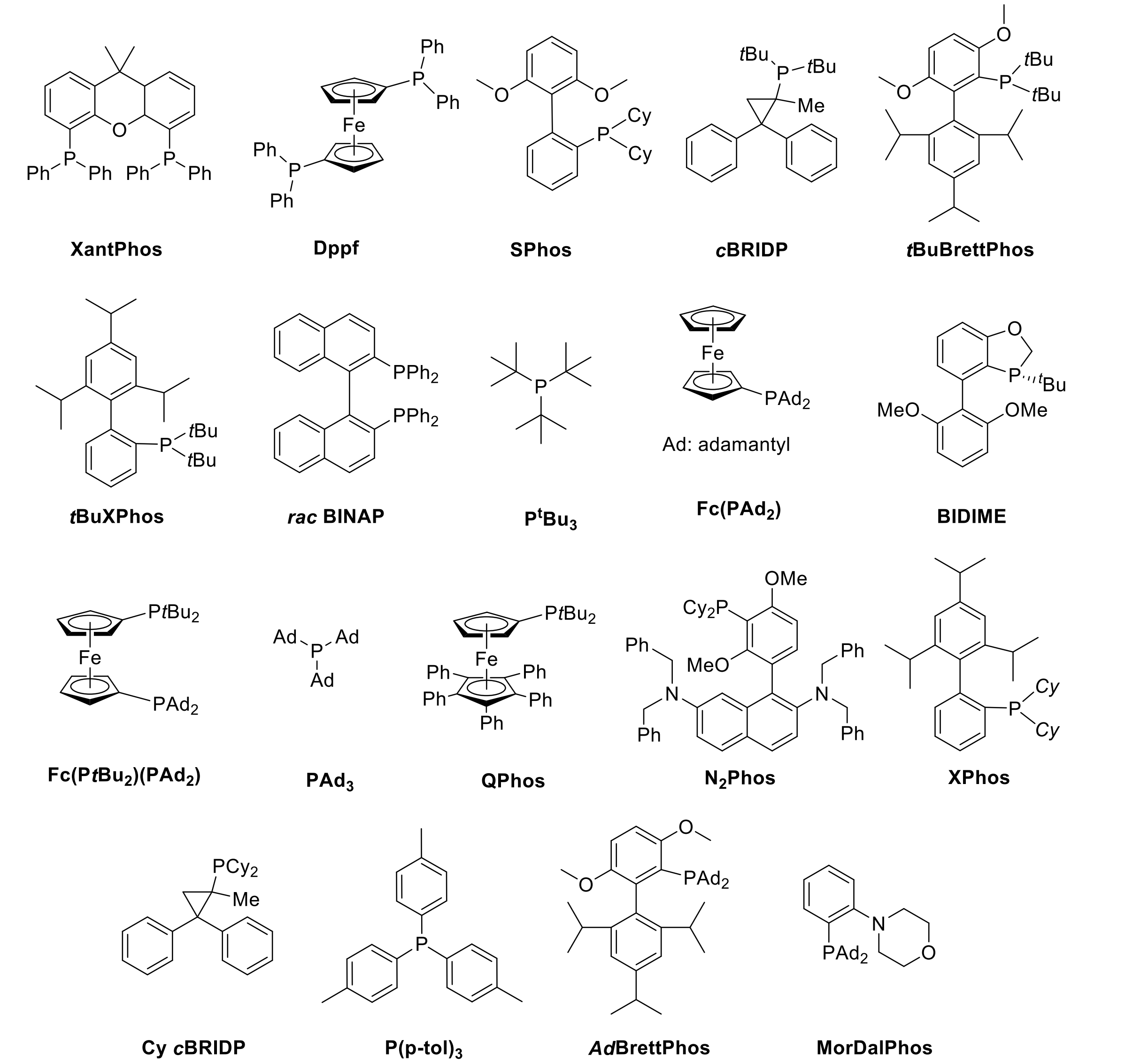

Figure S1. Structures of various ligands used for optimization.

3.1.2. Screening of palladium sources

Table S2. Screening of palladium sources for aryl bromides.

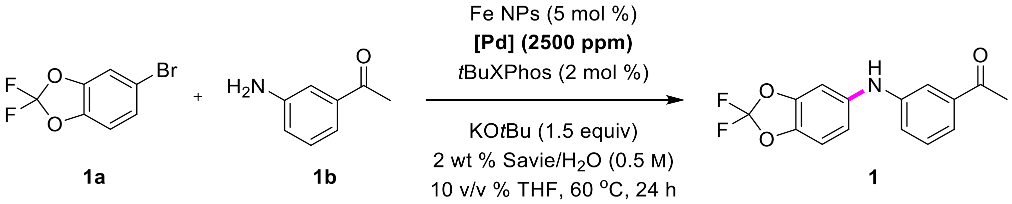

entry[a]	source of Pd	yield (%)[b]
1	$[Pd(allyl)Cl]_2$ (0.125 mol %)	55
2	$[Pd(cinnamyl)Cl]_2$ (0.125 mol %)	35
**3**	**$[Pd(crotyl)Cl]_2$ (0.125 mol %)**	**99**
**4**	**$Pd(dba)_2$ (0.25 mol %)**	**99**
5	$Pd(OAc)_2$ (0.25 mol %)	90

[a] Reaction conditions: 0.25 mmol **1a**, 0.375 mmol **1b**, Fe NPs (5 mol %, 10 mg), [Pd] source (50 µL, 2500 ppm, 0.25 mol %, administered as a stock solution in THF), *t*BuXPhos (2 mol %, 2.2 mg), KO*t*Bu (1.5 equiv, 42 mg), 2 wt % Savie/H_2O (0.45 mL, 0.5 M), 60 °C, 24 h; [b] NMR yields using 1,3,5–trimethoxybenzene as internal standard.

Note: Further optimization was carried out using $[Pd(crotyl)Cl]_2$ (2–butenylchloro palladium dimer, Sigma Aldrich catalog #700045) due to commercial availability and bench stability as opposed to the use of $Pd(dba)_2$, where the use of a glovebox is required.

Preparation of a stock solution of $[Pd(crotyl)Cl]_2$ in THF:

An oven (or flame) dried 1–dram vial, equipped with a PTFE coated magnetic stir–bar was sealed with a rubber septum and taken into an argon filled glovebox, where $[Pd(crotyl)Cl]_2$ (2.5 mg, 0.006 mmol) was added. The vial was taken out of the glovebox and degassed THF (1 mL, obtained from a solvent purification system) was added. The vial was allowed to stir at rt until the catalyst dissolves completely in THF. For a 0.25 mmol scale reaction, 50 µL of this stock solution results in 2500 ppm (0.25 mol %) of Pd (0.125 mol % of the dimer).

3.1.3. Screening of bases

Table S3. Screening of bases for coupling of aryl bromides with anilines.

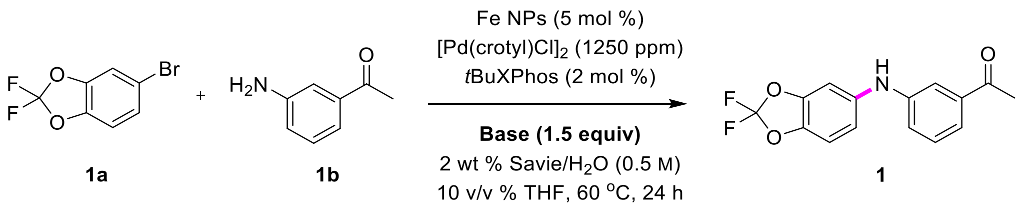

entry[a]	base	yield (%)[b]
1	NaO*t*Bu	90
2	KOH	70
**3**	**KO*t*Bu**	**99 (92)**[e]
**4**	**KOH / *t*BuOH (2 equiv)**	**98 (90)**[e]
5	KOAc	trace
6	$K_3PO_4 \cdot H_2O$	89
7	K_2CO_3	95
8	Cs_2CO_3	62
9	Et_3N	84
10	DBU[c]	26
11	KOTMS[d]	84

[a] Reaction conditions: 0.25 mmol **1a**, 0.375 mmol **1b**, Fe NPs (5 mol %, 10 mg), $[Pd(crotyl)Cl]_2$ (50 µL, 0.25 mol % Pd, 0.125 mol % of the dimer used, administered as a stock solution in THF), *t*BuXPhos (2 mol %, 2.2 mg) , base (1.5 equiv), 2 wt % Savie/H_2O (0.45 mL, 0.5 M), 60 °C, 24 h; [b] NMR yields using 1,3,5–trimethoxybenzene as internal standard; [c] DBU: 1,8–Diazabicyclo[5.4.0]undec–7–ene; [d] KOTMS: Potassium trimethylsilanoate; [e] isolated yield.

3.1.4. Surfactant screening

Table S4. Screening of various surfactants.

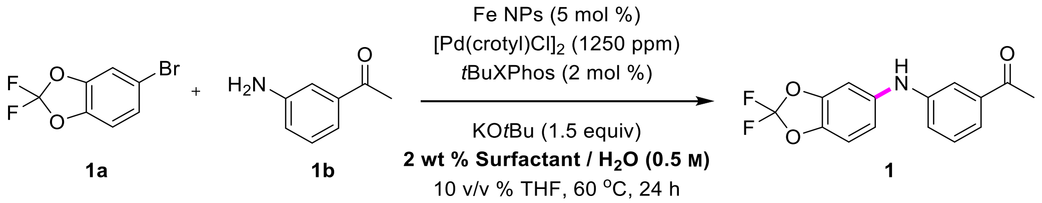

entry[a]	surfactant	yield (%)[b]
1	TPGS–750–M	75
2	Kolliphor ES	91
3	PTS 600	66
4	Triton–X	31
5	MC–1	85
**6**	**Savie**	**99**
7	H_2O only	63

[a] Reaction conditions: 0.25 mmol **1a**, 0.375 mmol **1b**, Fe NPs (5 mol %, 10 mg), $[Pd(crotyl)Cl]_2$ (50 µL, 0.25 mol % Pd, 0.125 mol % of the dimer used, administered as a stock solution in THF), *t*BuXPhos (2 mol %, 2.2 mg), KO*t*Bu (1.5 equiv, 42 mg), 2 wt % surfactant/H_2O (0.45 mL, 0.5 M), 60 °C, 24 h; [b] NMR yields using 1,3,5–trimethoxybenzene as internal standard.

3.1.5. Screening of amine equivalents

Table S5. Variation in amine equivalents with respect to aryl bromide.

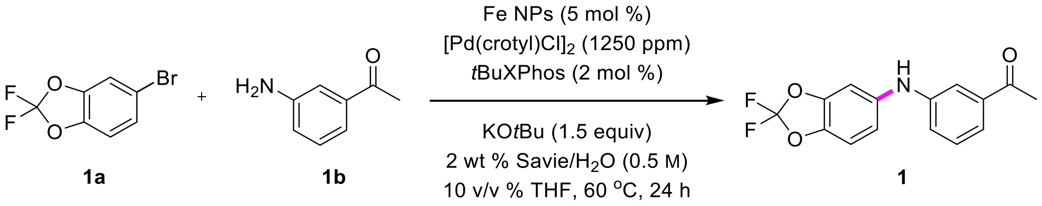

entry[a]	amine equivalents	yield (%)[b]
1	1.1	49
2	1.25	65
**3**	**1.5**	**99**

[a] Reaction conditions: 0.25 mmol **1a**, 0.375 mmol **1b**, FeNPs (5 mol %, 10 mg), $[Pd(crotyl)Cl]_2$ (50 µL, 0.25 mol % Pd, 0.125 mol % of the dimer used, administered as a stock solution in THF), *t*BuXPhos (2 mol %, 2.2 mg), KO*t*Bu (1.5 equiv), 2 wt % Savie/H_2O (0.45 mL, 0.5 M), 60 °C, 24 h; [b] NMR yields using 1,3,5–trimethoxybenzene as internal standard.

3.1.6. Catalyst loading screen

Table S6. Effect of different loadings of catalyst.

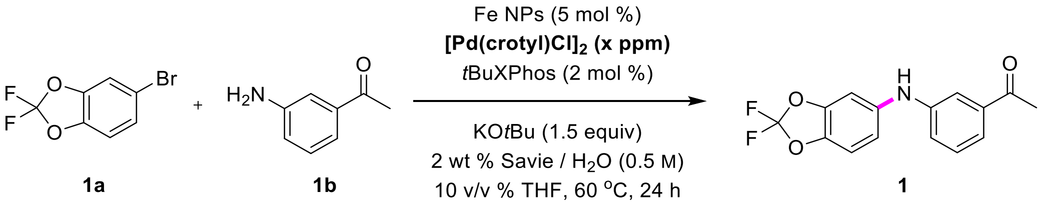

entry[a]	catalyst loading (Pd)	yield (%)[b]
1	1500 (0.075 mol % dimer)	45
2	2000 (0.1 mol % dimer)	78
**3**	**2500 (0.125 mol % dimer)**	**99**

[a] Reaction conditions: 0.25 mmol **1a**, 0.375 mmol **1b**, FeNPs (5 mol %, 10 mg), $[Pd(crotyl)Cl]_2$ (0.25 – 0.15 mol % Pd, 0.125 – 0.075 mol % of the dimer used, administered as a stock solution in THF), *t*BuXPhos (2 mol %, 2.2 mg), KO*t*Bu (1.5 equiv), 2 wt % Savie/H_2O (0.45 mL, 0.5 M), 60 °C, 24 h were carried out on a 0.25 mmol scale; [b] NMR yields using 1,3,5–trimethoxybenzene as internal standard.

3.1.7. Ligand loading screen

Table S7. Variation in the amount of ligand for coupling of aryl bromides and anilines.

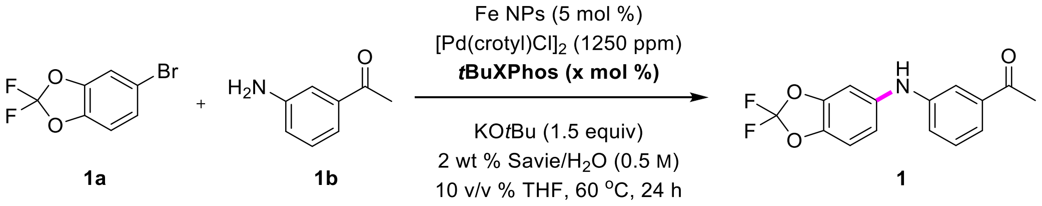

entry[a]	ligand loading	yield (%)[b]
**1**	**2 mol %**	**99**
2	1 mol %	70

[a] Reaction conditions: 0.25 mmol **1a**, 0.375 mmol **1b**, FeNPs (5 mol %, 10 mg), [Pd(crotyl)Cl]2 (0.25 – 0.15 mol % Pd, 0.125 – 0.075 mol % of the dimer used, administered as a stock solution in THF), *t*BuXPhos (2 mol %, 2.2 mg), KO*t*Bu (1.5 equiv), 2 wt % Savie/H_2O (0.45 mL, 0.5 M), 60 °C, 24 h; b NMR yields using 1,3,5–trimethoxybenzene as internal standard.

3.1.8. Temperature screening

Table S8. Variation in temperature for amination of aryl bromides with anilines.

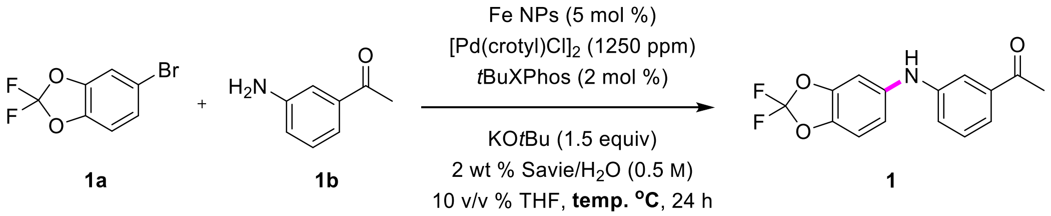

entry[a]	temperature (°C)	yield (%)[b]
**1**	**60**	**99**
2	55	95
3	45	81
4	35	40

[a] Reaction conditions: 0.25 mmol **1a**, 0.375 mmol **1b**, FeNPs (5 mol %, 10 mg), $[Pd(crotyl)Cl]_2$ (0.25 mol % Pd, 0.125 mol % of the dimer used, administered as a stock solution in THF), *t*BuXPhos (2 mol %, 2.2 mg), KO*t*Bu (1.5 equiv), 2 wt % Savie/H_2O (0.45 mL, 0.5 M), T °C, 24 h.; [b] NMR yields using 1,3,5–trimethoxybenzene as internal standard.

3.1.9. Additional control experiments

Table S9. Control experiments.

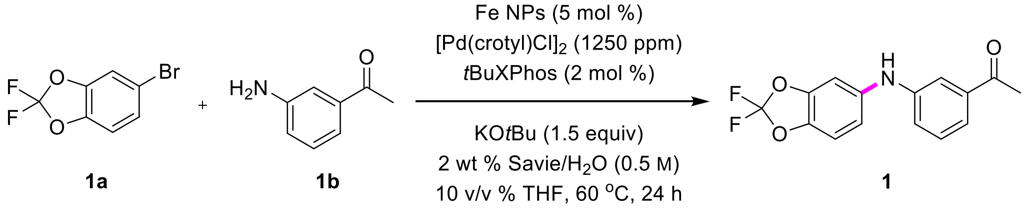

entry[a]	deviation from standard conditions	yield (%)[b]
**1**	**none**	**99 (91)[c]**
2	no Fe NPs	60
3	Fe NPs, no Pd	0
4	Fe NPs, no ligand	trace

[a] Reaction conditions: 0.25 mmol **1a**, 0.375 mmol **1b**, FeNPs (5 mol %, 10 mg), $[Pd(crotyl)Cl]_2$ (50 µL, 0.25 mol % Pd, 0.125 mol % of the dimer used, administered as a stock solution in THF), *t*BuXPhos (2 mol %, 2.2 mg), KO*t*Bu (1.5 equiv, 42 mg), 2 wt % Savie/H_2O (0.45 mL, 0.5 M), 60 °C, 24 h; [b] NMR yields using 1,3,5 –trimethoxybenzene as internal standard; [c] Isolated yield.

3.1.10. General procedure for coupling of aryl halides with anilines using Fe NPs: Procedure 1

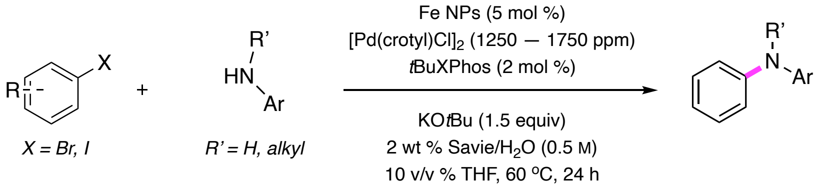

Scheme S1. Coupling of aryl halides with aromatic amines.

Reaction setup: To a 1–dram vial equipped with a PTFE coated magnetic stir–bar was added the aryl halide (1 equiv, 0.25 mmol, if solid) followed by the addition of the amine (0.375 mmol, 1.5 equiv, if solid). The vial was sealed with a rubber septum, evacuated, and backfilled with argon three times using an argon/vacuum manifold and then taken into an argon filled glovebox, where Fe NPs (10 mg, 5 mol %), *t*BuXPhos (2 mol %, 2.2 mg), and KO*t*Bu (1.5 equiv, 42 mg) were added. The vial was taken out of the glovebox and aryl halide was added (if liquid) followed by the addition of the amine (if liquid) under an atmosphere of argon. Subsequently, a solution of 2 wt % Savie/H_2O (0.45 mL) was added followed by the addition of $[Pd(crotyl)Cl]_2$ (0.25 – 0.35 mol % Pd, 0.125 – 0.175 mol % of dimer) as a stock solution in THF (50 µL; see SI, section 3.1.2). The reaction was allowed to stir at 60 °C for the designated amount of time (unless otherwise specified, see analytical section). Upon completion (as monitored by TLC), the reaction was extracted with EtOAc (4 x 1 mL). The combined extracts were dried over anhydrous Na_2SO_4, filtered, concentrated *in vacuo*, and subjected to flash chromatography using the desired eluent (EtOAc/hexanes or MeOH/CH_2Cl_2, see analytical section for respective eluents) to obtain the desired coupled products.

Note: For administering 0.175 mol % of $[Pd(crotyl)Cl]_2$, a new stock solution was prepared containing $[Pd(crotyl)Cl]_2$ (3.4 mg, 0.008 mmol) in anhydrous THF (1 mL), and 50 µL of this solution was used (see SI section 3.1.2).

3.2. General procedure for aminations using ocean water: Procedure 2

3.2.1. Preparation of a 2 wt % solution of Savie in ocean water

Ocean water (100 mL; from the Pacific Ocean) was first filtered through a medium porosity sintered glass funnel to remove any undissolved material and transferred into a 100 mL round bottom flask. Subsequently, the flask was sealed with a rubber septum and the water was degassed overnight with argon, employing a vent needle.

In an oven dried 6–dram vial equipped with a PTFE coated magnetic stir–bar was added Savie (100 mg). The vial was sealed with a rubber septum, evacuated, and backfilled with argon three times using an argon/vacuum manifold. Subsequently, 4.9 mL of degassed ocean water was added via a syringe and the solution was allowed to stir at rt until complete dissolution of Savie in water, resulting in a 2 wt % solution of Savie in ocean water.

3.2.2. Procedure for aminations using ocean water

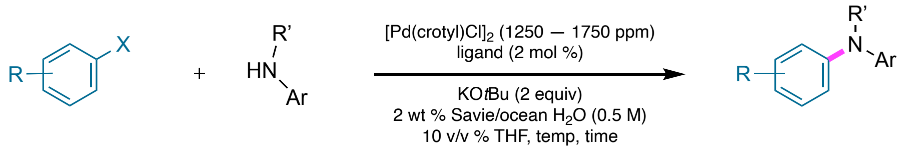

Scheme S2. Aminations using ocean water.

Reaction setup: To a 1–dram vial equipped with a PTFE coated magnetic stir–bar was added the aryl halide (1 equiv, 0.25 mmol, if solid) followed by the addition of the amine (0.375 mmol, 1.5 equiv, if solid). The vial was sealed with a rubber septum, evacuated, and backfilled with argon three times using an argon/vacuum manifold and then taken into an argon filled glovebox, where Fe NPs (5 mol %, 10 mg) (if coupling with an aniline is desired), ligand (*t*BuXPhos or BippyPhos) (2 mol %), and KO*t*Bu (2 equiv, 56 mg) were added. The vial was taken out of the glovebox and aryl halide was added (if liquid) followed by the addition of the amine (if liquid) under an atmosphere of argon. Subsequently, a solution of 2 wt % Savie/ocean H_2O (0.45 mL) was added followed by the addition of $[Pd(crotyl)Cl]_2$ (0.125–0.375 mol %, 0.25–0.75 mol % Pd) as a stock solution in THF (50 µL, see SI section 3.1.2). The reaction was allowed to stir at 60 °C (or 70 °C) for the designated amount of time. Upon completion (as monitored by TLC), the reaction was extracted with EtOAc (4 x 1 mL). The combined extracts were dried over anhydrous Na_2SO_4, filtered, concentrated *in vacuo*, and subjected to flash chromatography using the desired eluent

(EtOAc/hexanes or MeOH/CH_2Cl_2, see analytical section for respective eluents) to obtain the desired coupled products (This experiment was run on two substrates, compounds **3** and **32**; see Scheme 4).

Following the procedure above, product **3** was prepared at 60 °C for 18 h using 4–(5–bromopyrimidin–2–yl)morpholine (61 mg, 0.250 mmol), Indoline (44.6 mg, 42 µL, 0.375 mmol), 1250 ppm (0.125 mol %) $[Pd(crotyl)Cl]_2$ as catalyst, and 2 mol % *t*BuXPhos (2.2 mg) as ligand. Chromatography conditions: 10–20% EtOAc/hexanes. Yield: 67%, 47.3 mg; light yellow solid; R_f = 0.11 (10% EtOAc/hexanes, UV, CAM stain). For analytical data see ESI, section S10.

Following the procedure above, product **8** was prepared at 60 °C for 45 min using 2-bromo-3-fluoro-6-picoline (47.5 mg, 0.250 mmol), 3,4-dimethoxyaniline (59.3 mg, 0.375 mmol), 1250 ppm (0.125 mol %) $[Pd(crotyl)Cl]_2$ as catalyst, and 2 mol % *t*BuXPhos (225 mg) as ligand. Chromatography conditions: 10–20% EtOAc/hexanes. Yield: 93%, 61 mg; off white solid; R_f = 0.25 (20% EtOAc/hexanes, UV, CAM stain).

For analytical data see SI section S10.

4. Analyses of nanoparticles (NPs)

4.1. STEM–EDS analysis

a. Stem–EDS analysis of dry powder

The Fe NPs dry powder was loaded on the copper grid directly and imaged.

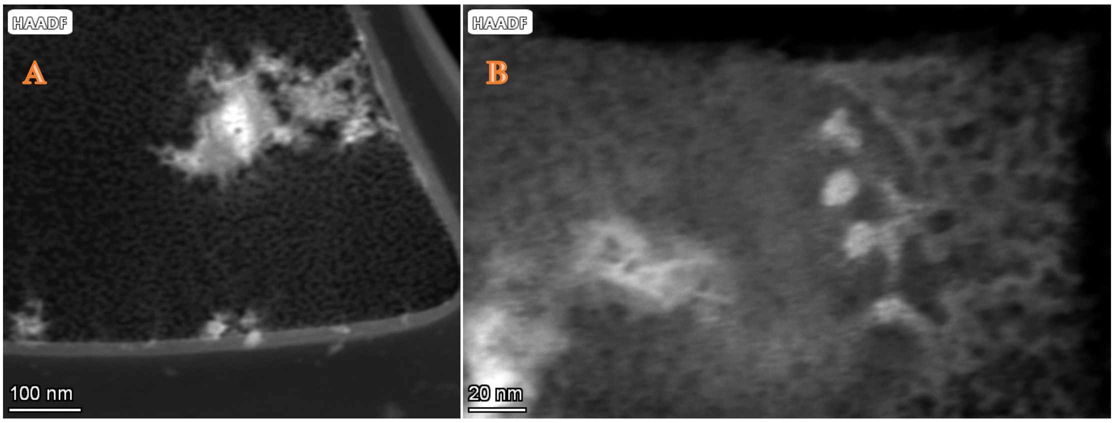

Figure S2. (A) HAADF image showing Fe NPs dry powder on 100 nm scale; (B) HAADF image showing Fe NPs dry powder on 20 nm scale.

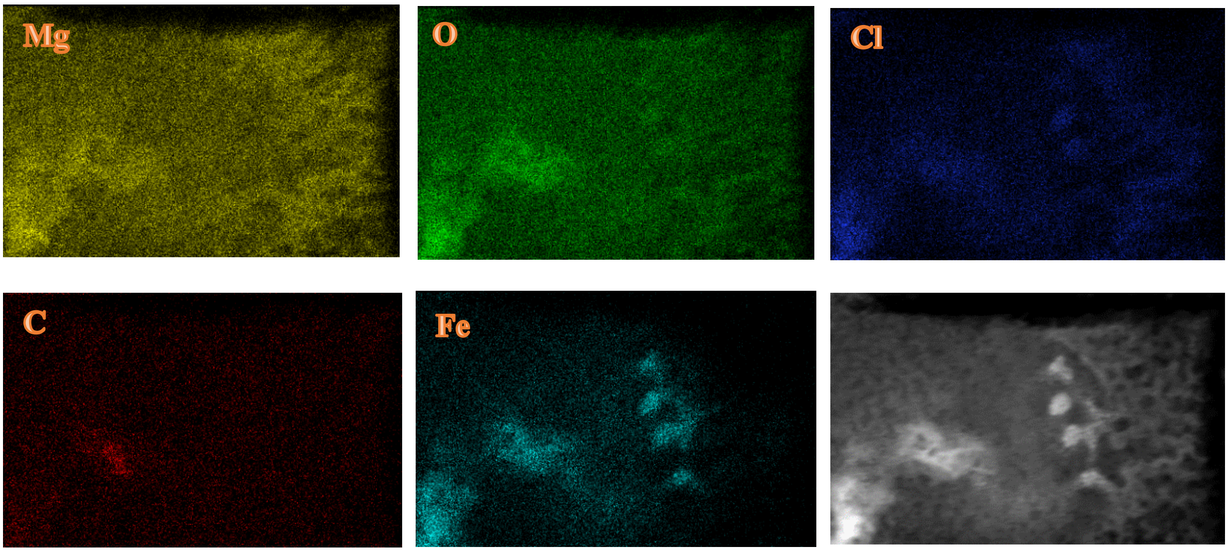

Figure S3. Element mapping of Figure **S1B** on Mg, O, Cl, C, Fe.

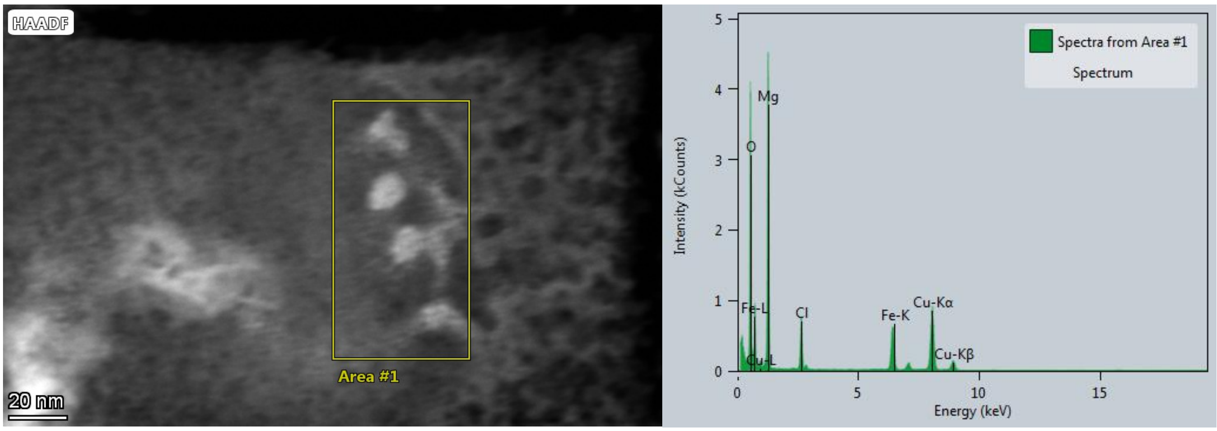

Figure S4. EDS analysis showing new Fe NPs in area 1.

b. **STEM–EDS analysis of NPs in degassed water**

The Fe NPs (2 mg) was added to a vial in the glove box, and the vial was covered with a rubber septum. Degassed water (2 mL) was added into the vial via syringe and the mixture was stirred for 1 h.

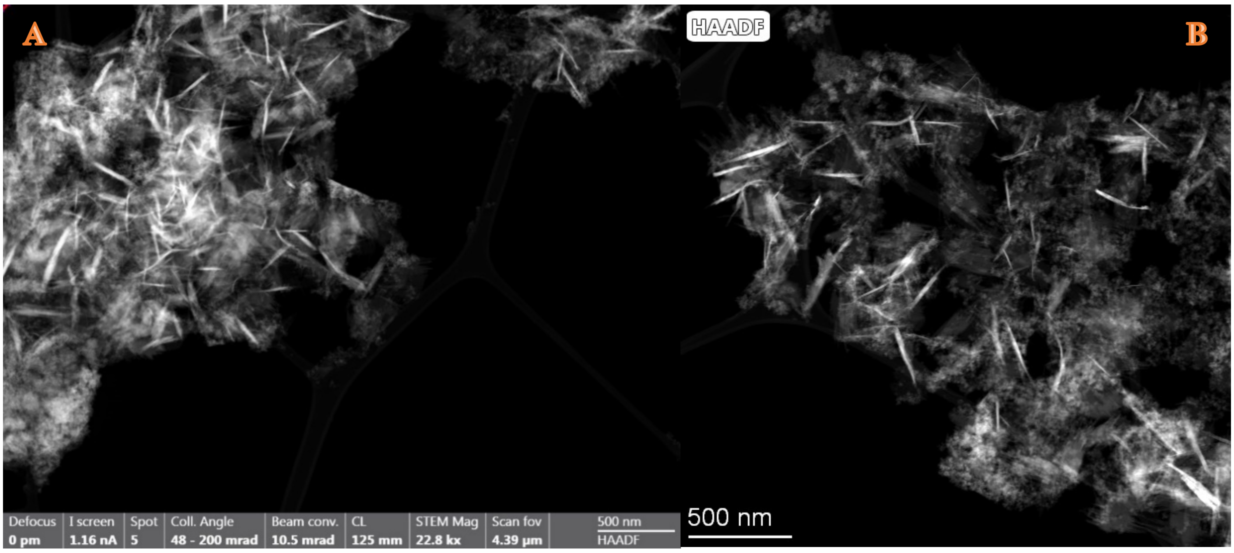

Figure S5. HAADF images showing Fe NPs in degassed water on 500 nm scale.

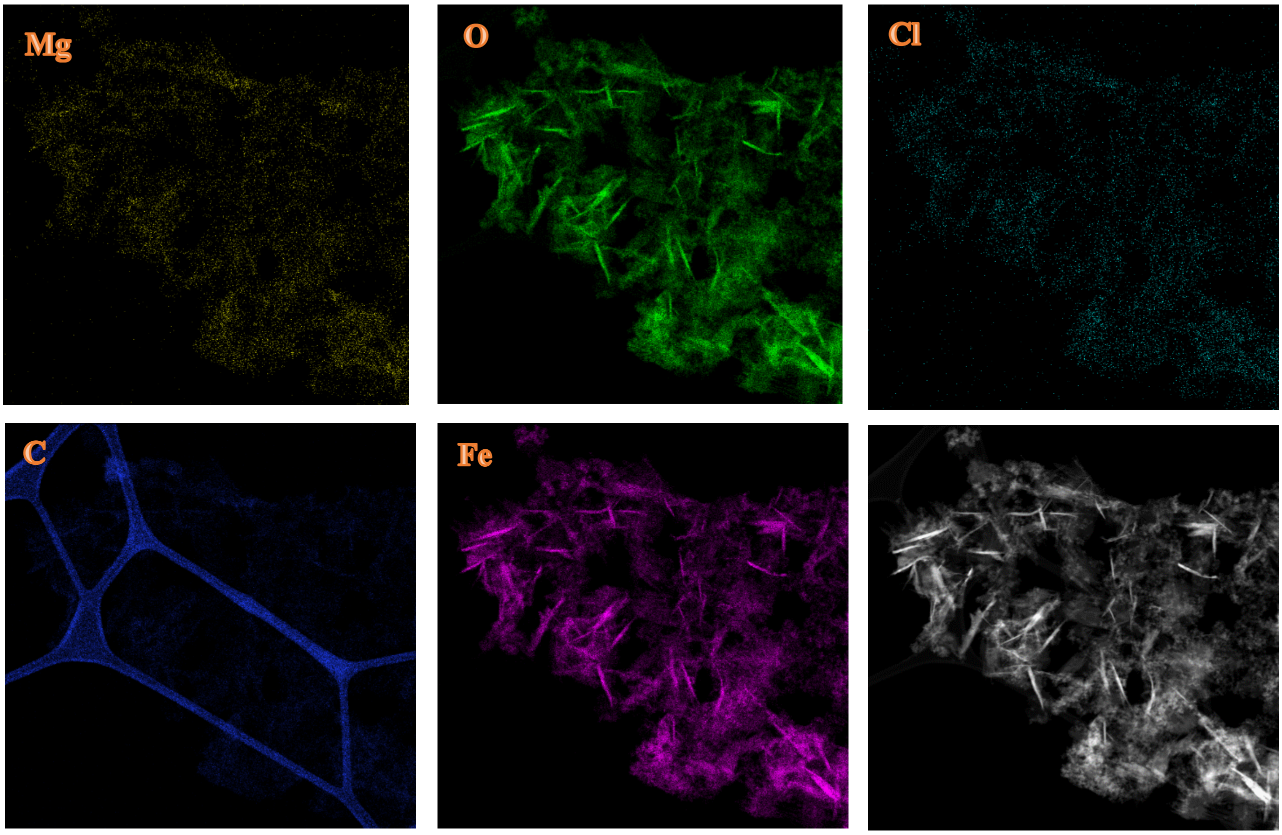

Figure S6. Element mapping of Figure **S4B** on Mg, O, Cl, C, Fe.

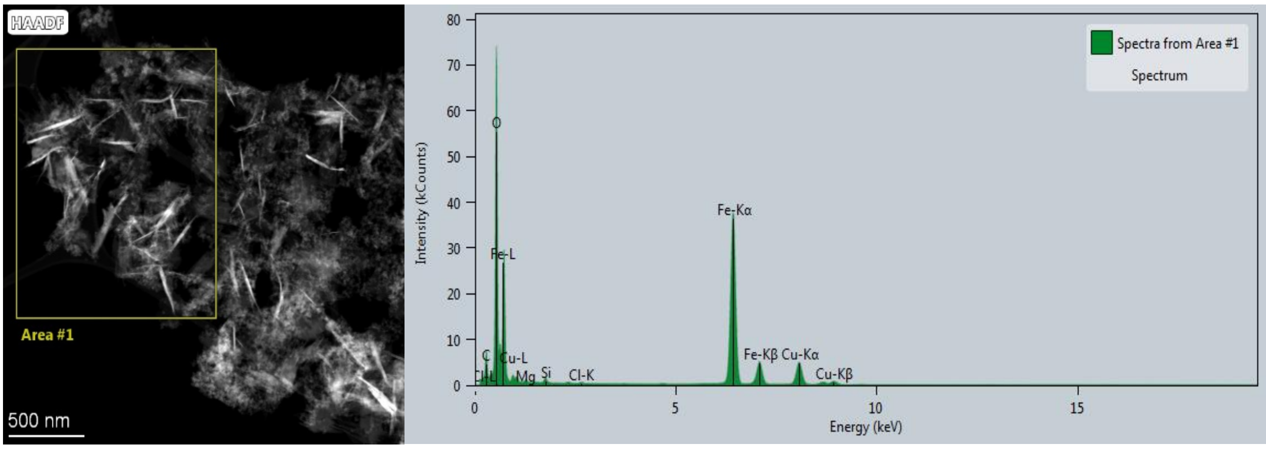

Figure S7. EDS analysis showing Fe NPs in degassed water in area 1.

c. STEM–EDS analysis of NPs in 2 wt % Savie/H_2O solution

The Fe NPs (2 mg) was added to a vial in the glove box, and the vial was covered with a rubber septum. 2 wt % Savie/H_2O (2 mL) was added into the vial via syringe and the mixture was stirred for 1 h.

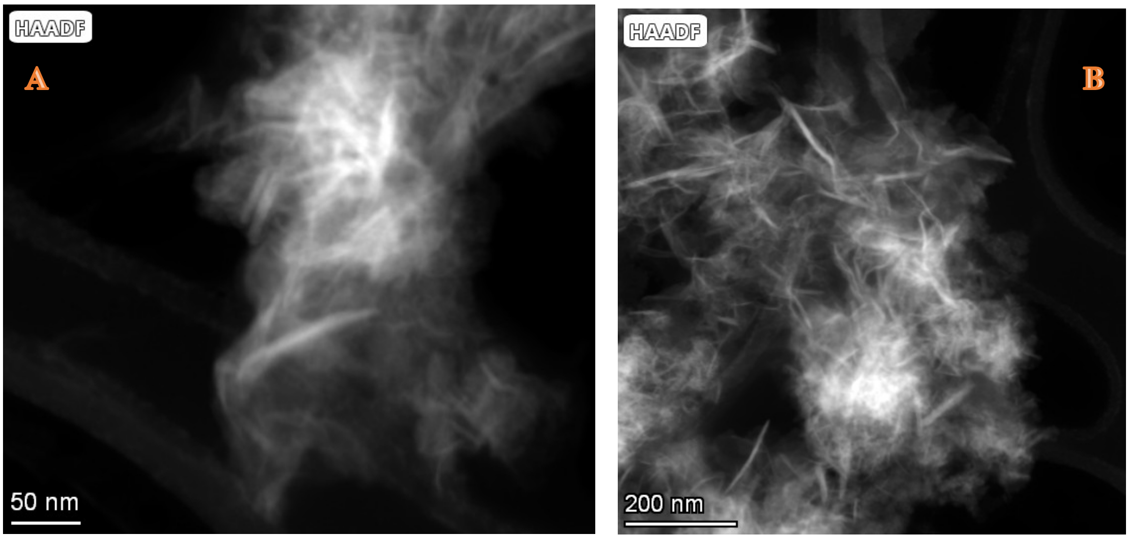

Figure S8. (A) HAADF image showing Fe NPs in 2 wt % Savie/H_2O solution on 50 nm scale; (B) HAADF image showing Fe NPs in 2 wt % Savie/H_2O solution on 200 nm scale.

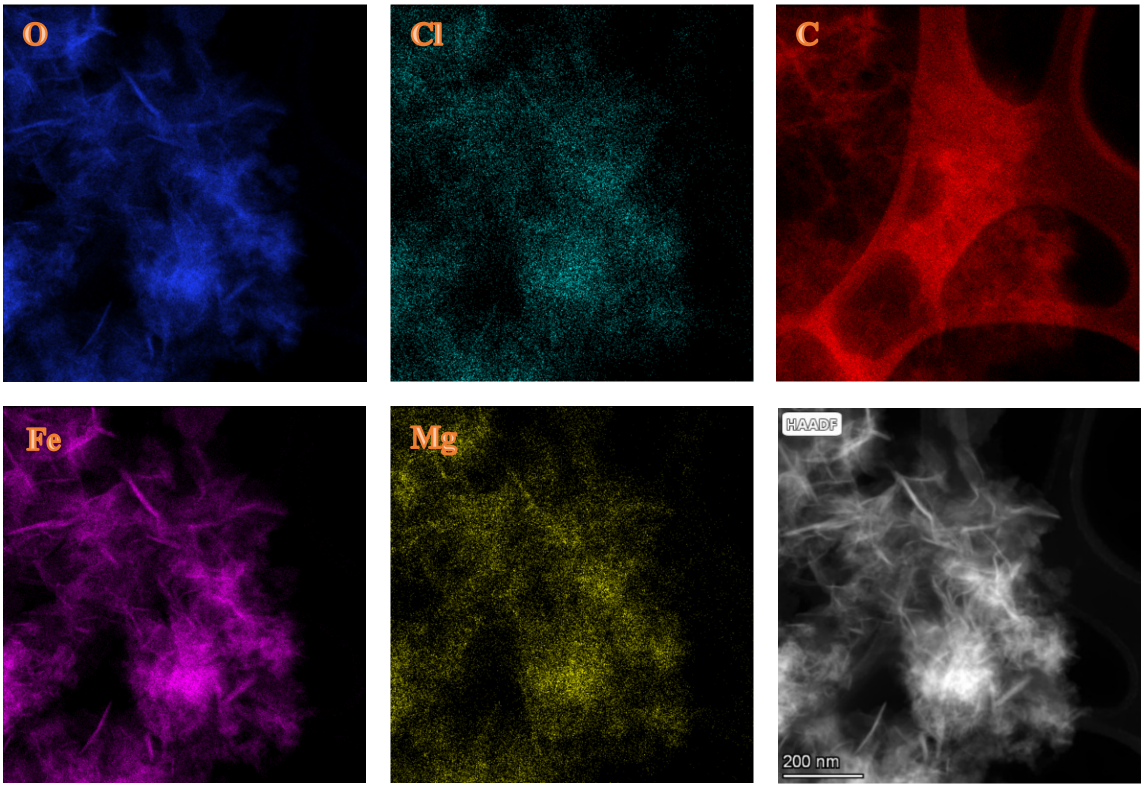

Figure S9. Elementary mapping of Figure **S7B** on Mg, O, Cl, C, Fe.

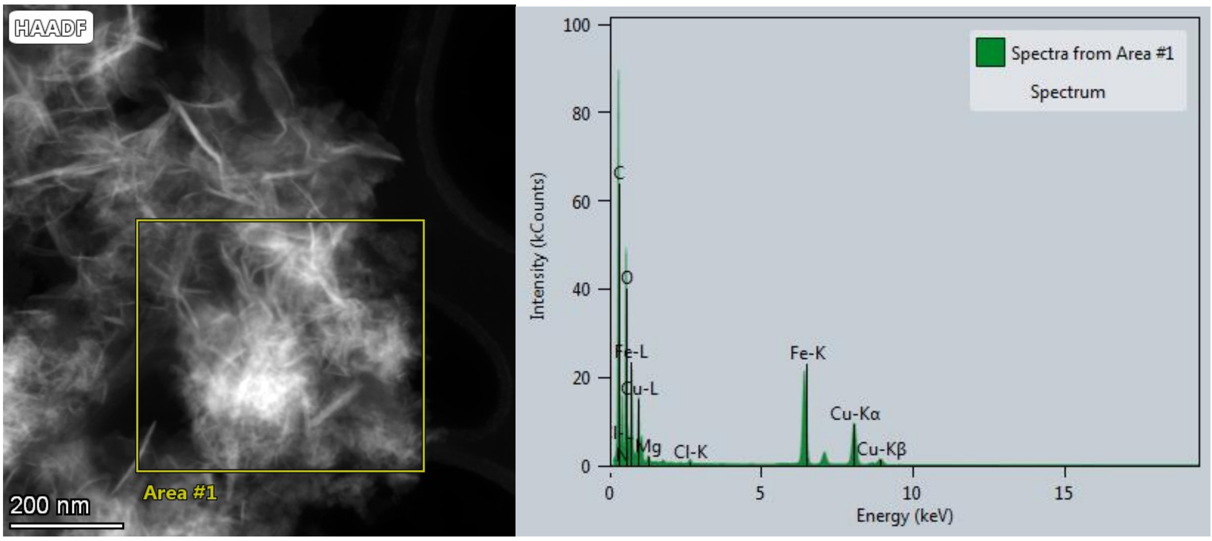

Figure S10. EDS analysis showing Fe NPs in 2 wt % Savie/H_2O solution in area 1.

4.2.Dynamic Light scattering (DLS) analysis

a. Dynamic light scattering (DLS) analysis of Fe NPs in degassed water

The Fe NPs (0.5 mg) was added to a vial in the glove box, and the vial was covered with a septum. Degassed water (2 mL) was added into the vial via syringe and the mixture was stirred for 1 h.

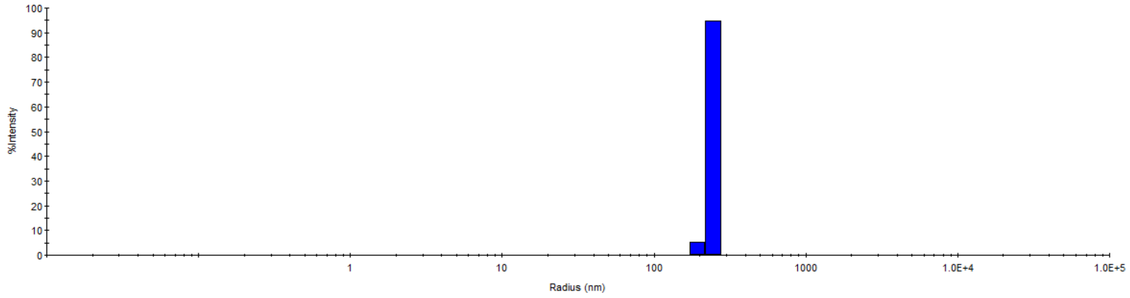

Peak	Radius (nm)	Mw–R (kDa)	PDI[a]	%Intensity	%Mass	%Number
Peak 2 (True)	244.2	1300357	4.7	100	100	100

[a] PDI = % polydispersity.

Figure S11. DLS analysis of intensity (%) versus radius (nm) with peak at 244.2 nm.

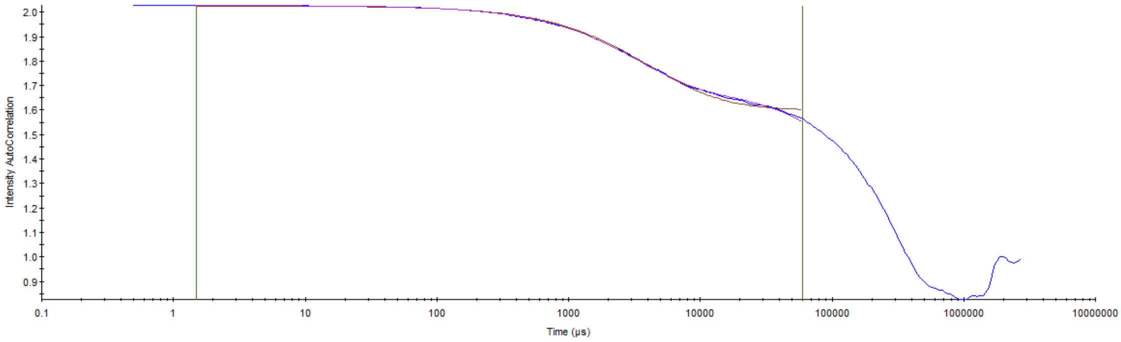

Figure S12. Intensity autocorrelation of Figure **S10**.

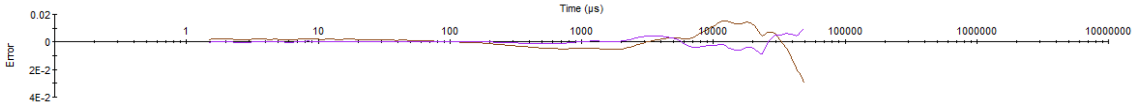

Figure S13. Error analysis of Figure **S10**.

b. Dynamic light scattering analysis (DLS) of Fe NPs in 2 wt % Savie/H_2O solution

The Fe NPs (0.5 mg) was added to a vial in the glove box, and the vial was covered with a septum. 2 wt % Savie/H_2O solution (2 mL) was added into the vial via syringe and the mixture was stirred for 1 h.

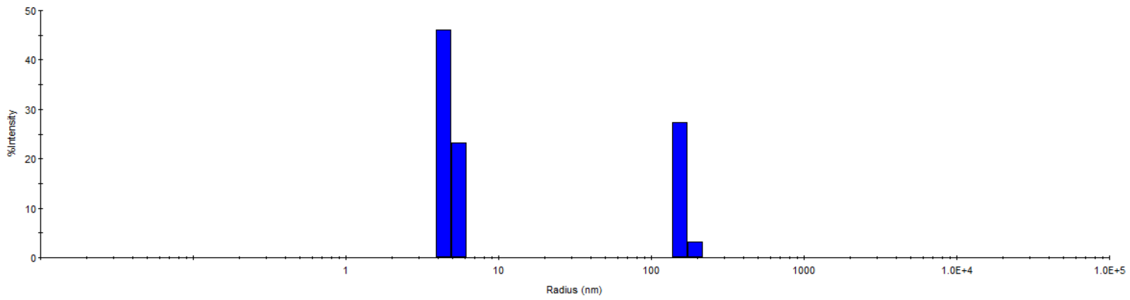

Peak	Radius (nm)	Mw–R (kDa)	PDI[a]	%Intensity	%Mass	%Number
Peak 1 (True)	4.7	128.5	11.6	69.3	99.8	100
Peak 2 (True)	157.9	468772.7	8	30.7	0.2	0

[a] PDI = % polydispersity.

Figure S14. DLS analysis of intensity (%) versus radius (nm) with peak at 4.7 nm and 157.9 nm.

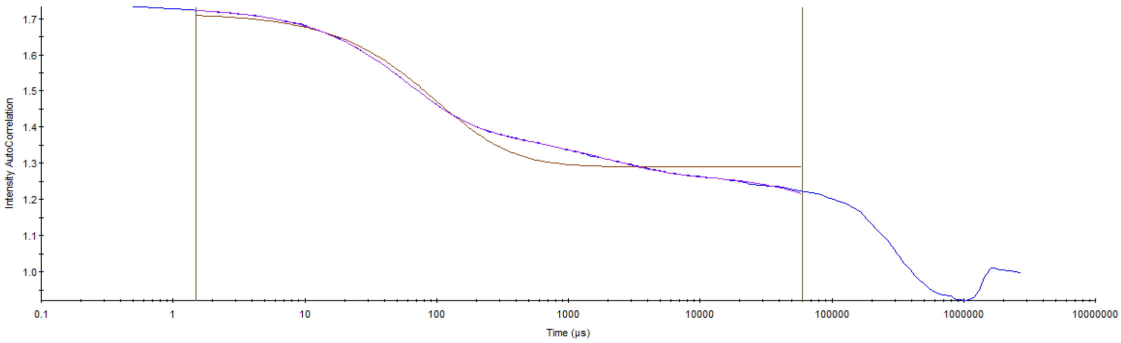

Figure S15. Intensity autocorrelation of Figure **S10**.

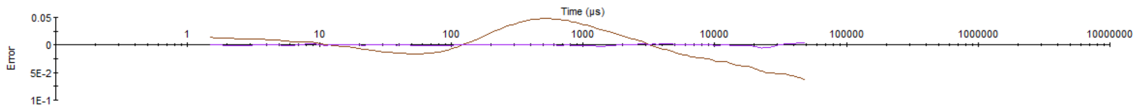

Figure S16. Error analysis of Figure **S10**.

5. Recycling studies and E Factor calculations

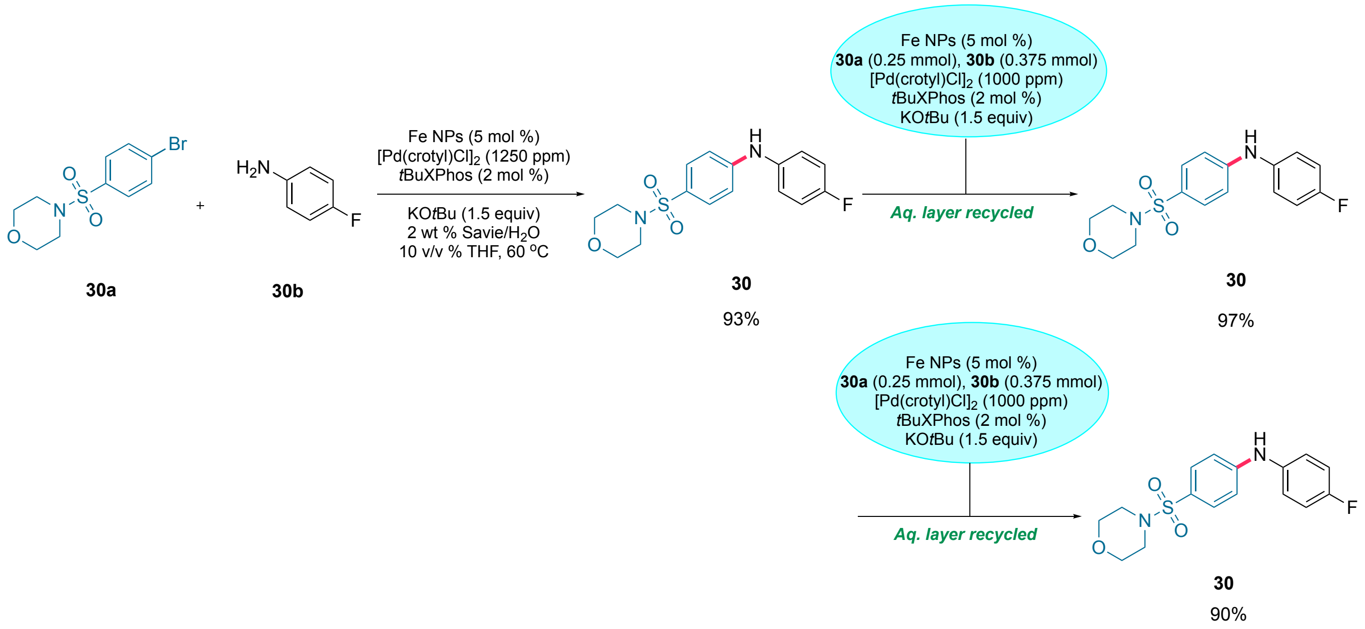

Scheme S3. Representative example demonstrating the recyclability of the aqueous medium.

5.1. Recycling studies

1st Reaction:

To a 1–dram vial equipped with a PTFE coated magnetic stir–bar was added 4–((4–bromophenyl)sulfonyl)morpholine **30a** (1 equiv, 0.25 mmol, 76.5 mg) followed by the addition of 4–fluoroaniline **30b** (0.375 mmol, 1.5 equiv, 42, 36 µL). The vial was sealed with a rubber septum, evacuated, and backfilled with argon three times using an argon/vacuum manifold and then taken into an argon filled glovebox, where Fe NPs (5 mol %, 10 mg), *t*BuXPhos (2 mol %, 2.2 mg), and KO*t*Bu (1.5 equiv, 42 mg) were added. The vial was taken out of the glovebox and under an atmosphere of argon, a solution of 2 wt % Savie/H_2O (0.45 mL) was added followed by the addition of $[Pd(crotyl)Cl]_2$ (1250 ppm, 0.25 mol % Pd, 0.125 mol % of dimer) as a stock solution in THF (50 µL, see SI, section 3.1.2). The reaction was allowed to stir at 60 °C for 2 h. Upon completion (as monitored by TLC), the reaction was extracted with EtOAc (2 x 0.6 mL). The organic layer was carefully decanted out via syringe. The combined organic extracts were concentrated *in vacuo* and purified by flash chromatography over silica gel (20-30% EtOAc/hexanes) to afford 4–fluoro–*N*–(4–(morpholinosulfonyl)phenyl)aniline **30** as a tan solid (78 mg, 93% yield).

2nd reaction (1st recycle):

To the same vial containing the aqueous layer from the previous reaction was added 4–((4–bromophenyl)sulfonyl)morpholine **30a** (1 equiv, 0.25 mmol, 76.5 mg) followed by the addition of 4–fluoroaniline **30b** (0.375 mmol, 1.5 equiv, 42, 36 µL). Subsequently, Fe NPs (5 mol %, 10 mg), *t*BuXPhos (2 mol %, 2.2 mg), and KO*t*Bu (1.5 equiv, 42 mg) were quickly added under an atmosphere of argon and the vial was sealed with a rubber septum and purged with argon for 5 min, employing a vent needle. Then, $[Pd(crotyl)Cl]_2$ (1000 ppm, 0.2 mol % Pd, 0.1 mol % of dimer) was added as a stock solution in THF (40 µL, see SI, section 3.1.2), and the reaction was stirred at 60 °C for 2 h. Upon completion (as monitored by TLC), the reaction was extracted with EtOAc (2 x 0.6 mL). The organic layer was carefully decanted out via syringe. The combined organic extracts were concentrated *in vacuo* and purified by flash chromatography over silica gel (20-30% EtOAc/hexanes) to afford 4–fluoro–*N*–(4–(morpholinosulfonyl)phenyl)aniline **30** as a tan solid (82 mg, 97% yield).

3rd reaction (2nd recycle):

To the same vial containing the aqueous layer from the previous reaction was added 4–((4–bromophenyl)sulfonyl)morpholine **30a** (1 equiv, 0.25 mmol, 76.5 mg) followed by the addition of 4–fluoroaniline **30b** (0.375 mmol, 1.5 equiv, 42, 36 µL). Subsequently, Fe NPs (5 mol %, 10 mg), *t*BuXPhos (2 mol %, 2.2 mg), and KO*t*Bu (1.5 equiv, 42 mg) were quickly added under an atmosphere of argon and the vial was sealed with a rubber septum and purged with argon for 5 min, employing a vent needle. Then, $[Pd(crotyl)Cl]_2$ (1000 ppm, 0.2 mol % Pd, 0.1 mol % of dimer) was added as a stock solution in THF (40 µL, see SI, section 3.1.2), and the reaction was stirred at 60 °C for 2 h. Upon completion (as monitored by TLC), the reaction was extracted with EtOAc (2 x 0.6 mL). The organic layer was carefully decanted out via syringe. The combined organic extracts were concentrated *in vacuo* and purified by flash chromatography over silica gel (20 – 30% EtOAc/hexanes) to afford 4–fluoro–*N*–(4–(morpholinosulfonyl)phenyl)aniline **30** as a tan solid (76 mg, 90% yield).

At this stage, the aqueous phase had become too viscous and saturated with salts, and hence further recycling studies were not pursued.

Stock solution preparation: 2.5 mg of $[Pd(crotyl)Cl]_2$ *was dissolved in 1 mL degassed THF (obtained from a solvent purification system). This stock solution (50 µL) corresponds to 2500 ppm (0.25 mol %) of Pd loading (0.125 mol % Pd dimer). This stock solution (40 µL) corresponds to 2000 ppm (0.2 mol %) of Pd loading (0.1 mol % Pd dimer).*

5.2.E Factor calculations

$$\textbf{E - factor} = \frac{\textbf{mass of organic waste}}{\textbf{mass of product}}$$

$$\text{E - factor (with water)} = \frac{\text{mass of organic waste + mass of water}}{\text{mass of product}}$$

Organic waste:

1. Excess reagents

   - Amine **30b**: 0.5 equiv excess used in 3 reactions.

     Mass: (0.25 x 0.5 x 111.04 x 3) = **41.6 mg (0.0416 g)**

2. Extraction solvent

   - EtOAc: (0.6 x 2) = 1.2 mL used in each reaction

     Total volume = (1.2 x 3) = 3.6 mL
     Density of EtOAc = 0.9 g/cm^3

     Total mass of EtOAc = (3.6 x 0.9) = **3.24 g**

3. Mass of water = **0.45 g**

Mass of product = (0.078 + 0.082 + 0.076) = **0.226 g**

- $$\text{E - factor (with water)} = \frac{3.24 + 0.45 + 0.0416}{0.226}$$

  = **16.5**

- $$\text{E - factor (without water)} = \frac{3.24 + 0.0416}{0.226}$$

  = **14.5**

- E - factor (without extraction solvent) = $\frac{0.45 + 0.0416}{0.226}$

$$= \mathbf{2.1}$$

Note: The reactions are extracted purely with EtOAc, and is concentrated in–vacuo, hence can be potentially recycled by distillation on a larger scale.

6. General procedure for gram–scale synthesis of compound 6 – Procedure 3

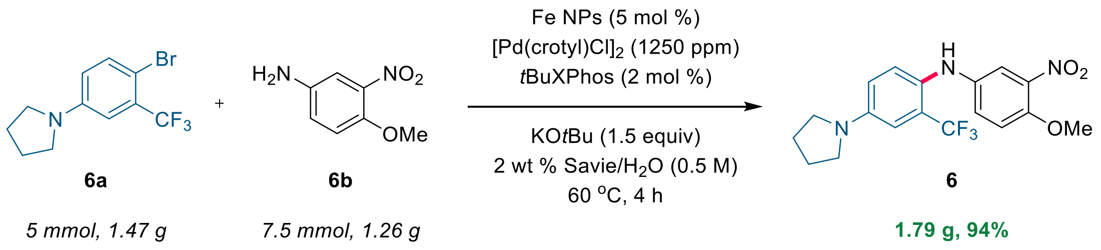

Scheme S4. Gram scale synthesis of compound **6**.

Reaction setup: To an oven–dried 250 mL round bottom flask equipped with a PTFE coated magnetic stir–bar was added 1–(4–bromo–3–(trifluoromethyl)phenyl)pyrrolidine **6a** (1 equiv, 5 mmol, 1.47 g) followed by the addition of 4–methoxy–3–nitroaniline **6b** (7.5 mmol, 1.5 equiv, 1.26 g). The flask was sealed with a rubber septum, evacuated, and backfilled with argon three times using an argon/vacuum manifold and then taken into an argon filled glovebox, where Fe NPs (5 mol %, 200 mg), $[Pd(crotyl)Cl)]_2$ (1250 ppm, 0.125 mol %, 2.5 mg), *t*BuXPhos (2 mol %, 42.4 mg), and KO*t*Bu (1.5 equiv, 841 mg) were added. The flask was taken out of the glovebox and a solution of 2 wt % Savie/H_2O (10 mL, 0.5 M) was added under a positive flow of argon. The reaction was allowed to stir at 60 °C for 4 h. Upon completion (as monitored by TLC), EtOAc was added, and the reaction was stirred at rt for 2–3 min. Stirring was then stopped, and the reaction mixture was filtered through a pad of Celite and rinsed with EtOAc (50 mL). Subsequently, the mixture was transferred into a separatory funnel, and washed with brine . The organic layer was collected, and the same procedure was repeated twice (washing with brine). The combined extracts were dried over anhydrous Na_2SO_4, filtered, concentrated *in vacuo*, to around 10% of the solvent volume, wherein pentane was added to precipitate the product out. The resulting mixture was filtered using a Buchner funnel, and subsequently washed with a solution of 20% Et_2O/pentane (50 mL) and was dried overnight to afford pure **6** (1.79 g, 94%) as a dark orange solid. For analytical data see SI, section S10.

7. 5–Step, one–pot sequence to afford compound 52

7.1. Optimization of *N*–Boc deprotection on model substrate 47

The optimization of the *N*–Boc deprotection was carried out on model compound **47** (compound from step 1).

Reaction setup: To a 1–dram vial with a PTFE coated magnetic stir–bar was added compound **47** (1 equiv, 0.1 mmol, 40 mg), followed by the addition of the respective acid source (HCl or TFA, 5 equiv). The reaction was allowed to stir at 50 °C for 6 h. Upon completion, the crude reaction mixture was concentrated in vacuo to remove most of the HCl (or TFA) and then precipitated with diethyl ether. The resulting reaction mixture was centrifuged, and the ether layer was decanted via pipette. This procedure was repeated two more times and the resulting solid was dried under high vacuum to afford crude compound **47b**. Crude NMRs were taken in CD_3OD, and conversions were determined based on the disappearance of the Boc group.

Table S12. Optimization of *N*–Boc deprotection on model compound **47**.

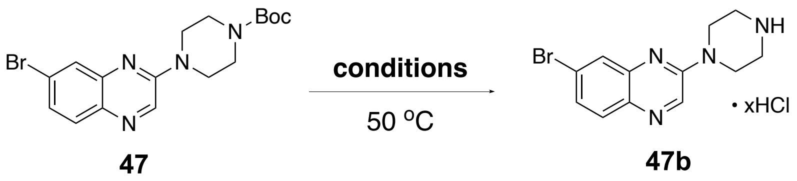

entry[a]	conditions	conversion (%)[b]
1	1 M HCl in EtOAc (5 equiv)	30
2	4 M HCl in dioxane (5 equiv)	75
**3**	**1 M HCl in AcOH (5 equiv)**	**>99**
4	4 M HCl (in H_2O) (5 equiv)	58
5	3 M HCl in MeOH (5 equiv)	85
6	TFA (neat, 5 equiv)	70

[a] Reactions were carried out on a 0.1 mmol scale for 6 h; [b] conversions were determined by crude NMR analysis.

7.2. General procedure for the synthesis of thioester 50: Procedure 4

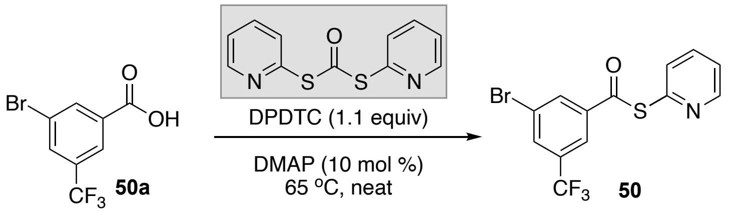

Scheme S5. Synthesis of thioester **50**.

This procedure was adapted from Freiberg and co–workers.[3]

To a 2–dram vial equipped with a PTFE coated magnetic stir–bar was added 3–bromo–5–trifluoromethylbenzoic acid **50a** (1 equiv, 1 mmol, 269 mg) followed by the addition of DPDTC (di–2–pyridyldithiocarbonate; 1.1 equiv, 1.1 mmol, 273.15 mg) and DMAP (10 mol %, 12.2 mg). The reaction mixture was stirred at 65 °C until full consumption of the carboxylic acid was observed by TLC or GC–MS. Upon completion, the reaction was diluted with EtOAc (3 mL) and was subsequently washed with 1 M NaOH (2 x 1 mL), to remove 2–mercaptopyridine. The resulting organic layer was concentrated *in vacuo* to afford crude *S*–(pyridin–2–yl)-3–bromo–5–(trifluoromethyl)benzothioate **50** as a yellow oil. This was diluted with minimal amounts of EtOAc (0.2 mL) and was used in step 4 without further purification.

7.3. General procedure for the 5–step, 1–pot sequence to afford 52: Procedure 5

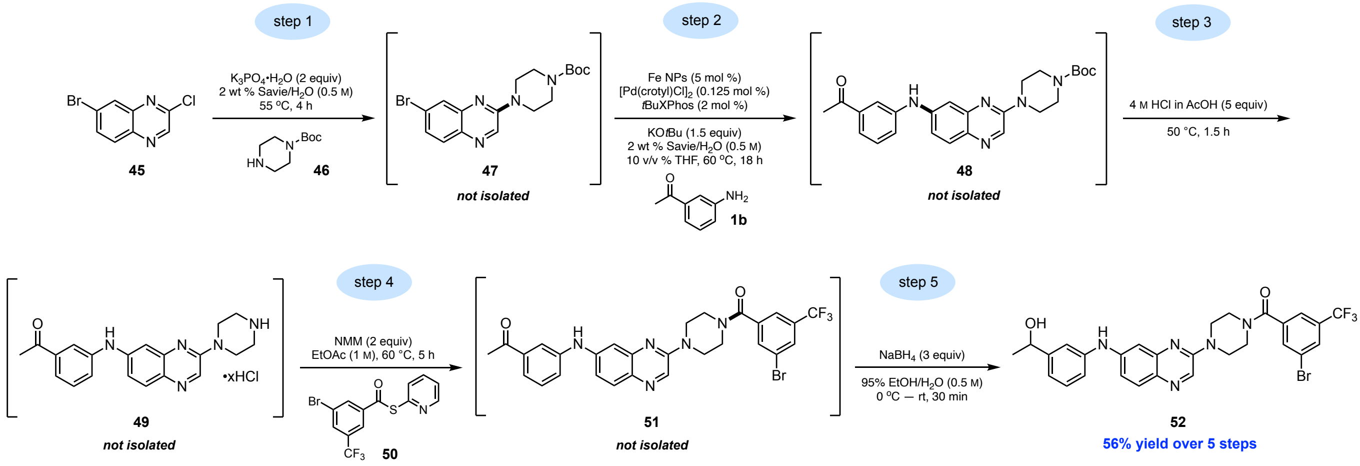

Scheme S6. 5-step one-pot sequence to afford **52**.

Step 1: S_NAr reaction

To a 2–dram vial equipped with a PTFE coated magnetic stir–bar was added 7–bromo–2–chloroquinoxaline **45** (1 equiv, 0.5 mmol, 122 mg), *t*–butyl piperazine–1–carboxylate **46** (1.5 equiv, 0.75 mmol, 139.68 mg) and K_3PO_4•H_2O (2 equiv, 1 mmol, 230.28 mg). The vial was sealed with a rubber septum, and subsequently, a solution of 2 wt % Savie/H_2O (0.5 M, 1 mL) was added via a syringe. The resulting reaction mixture was stirred at 55 °C for 4 h (reaction progress was monitored by TLC). (Note: The reaction mixture turns bright yellow upon completion).

Step 2: Pd–catalyzed amination

Upon completion of the previous step, the rubber septum was removed and 3–aminoacetophenone **1b** (1.5 equiv, 0.75 mmol, 102 mg) was added followed by the quick addition of Fe NPs (5 mol %, 20 mg), KO*t*Bu (1.5 equiv, 0.75 mmol, 84 mg) and *t*BuXPhos (2 mol %, 4.4 mg), under a continuous stream of argon and the vial was sealed with a rubber septum and purged with argon for 5 min, employing a vent needle. $[Pd(crotyl)Cl]_2$ (1750 ppm, 100 µL, 3500 ppm of Pd) was then added as a stock solution in THF (obtained from a solvent purification system, see SI, section 3.1.2), and the resulting reaction mixture was stirred at 60 °C for 12 h. Upon completion (as monitored by TLC), the crude reaction mixture was allowed to cool to rt and then diluted with a solution of 5% MeOH/CH_2Cl_2 (3 mL), and the layers were allowed to separate. The aqueous layer (top layer) was carefully removed via a syringe and the resulting organic layer was concentrated *in vacuo* to afford crude **48**. This was used in the subsequent step without further purification.

Step 3: *N*–Boc Deprotection

The crude reaction mixture from step 2 was subjected to a solution of 1 M HCl in AcOH (5 equiv, 2.5 mL), and the reaction was allowed to stir at 50 °C for 1.5 h. Upon completion (as monitored by TLC and crude NMR for the disappearance of the Boc group), the crude reaction mixture was concentrated *in vacuo* to remove most of the HCl and AcOH and was subsequently triturated with diethyl ether (3 mL), centrifuged, and the supernatant was decanted via a pipette (this process was repeated 3 times). The resulting solids were dried under high vacuum to afford crude **49** as a dark orange colored solid. This was used in the subsequent step without further purification.

Step 4: Amide bond formation

To the crude reaction mixture from step 3 was added crude *S*–(pyridin–2–yl) 3–bromo–5–(trifluoromethyl)benzothioate **50** (1.5 equiv, see section 6.2), as a solution in EtOAc (0.2 mL) followed by the addition of *N*–methylmorpholine (4 equiv, 2 mmol, 220 µL) and EtOAc (0.3 mL, the global concentration was adjusted to 1 M). The reaction was stirred at 60 °C for 5 h (the reaction was monitored

by TLC). Upon completion, the crude reaction mixture was concentrated *in vacuo* to remove any EtOAc, then diluted with a solution of 10% MeOH/CH_2Cl_2, followed by an aqueous workup with 1 M NaOH solution (2 x 2 mL), to remove residual 2–mercaptopyridine. The resulting organic layer was concentrated *in vacuo*, in the same vial to afford crude **51** as a yellow solid. This was used in the next step without further purification.

Step 5: Ketone reduction to the alcohol

The crude reaction mixture from step 4 was cooled in an ice bath, followed by the addition of 95% EtOH/H_2O (1 mL, 0.5 M), and then treated with sodium borohydride (3 equiv, 57 mg). Evolution of gas was observed. The reaction was then allowed to stir at rt for 30 min until completion, as monitored by TLC. Upon completion, the reaction mixture was concentrated *in vacuo* to remove most of the EtOH, then diluted with water, and extracted with a solution of 10% MeOH/CH_2Cl_2 (3 x 2 mL). The combined organic layers were filtered through a small pad of Celite to remove any inorganics and residual water, concentrated *in vacuo* and subjected to flash chromatography (0.5–3% MeOH/CH_2Cl_2) to afford the final product **52** as a yellow crystalline solid (yield: 56% over 5 steps).
(Note: The final product **52** is slightly contaminated with grease originating from the solvent, so the amount of grease present is taken into consideration when calculating the yield).

8. ICP–MS analysis for residual palladium

ICP–MS data for residual Pd was obtained from the University of California, Los Angeles, ICP–MS Core Facility.

		**palladium** [μg/g]		**sample source**
sample #	sample weight in analysis [mg]	average*	std dev	
KI–943-3	7.60	**1.586**	0.135	recycling studies – Reaction 3
KI–874–5	5.40	**0.600**	0.005	one–pot sequence – final compound **52**

*Each sample was run in triplicate, with background correction.

9. References

1. Lipshutz, B. H.; Ghorai, S.; Abela, A. R.; Moser, R.; Nishikata, T.; Duplais, C.; Krasovskiy, A.; Gaston, R. D.; Gadwood, R. C. TPGS–750–M: A Second–Generation Amphiphile for Metal–Catalyzed Cross Couplings in Water at Room Temperature. *J. Org. Chem.* **2011**, *76*, 4379−4391.
2. Kincaid, J. R. A.; Wong, M. J.; Akporji, N.; Gallou, F.; Fialho, D. M.; and Lipshutz, B. H. Introducing Savie: A Biodegradable Surfactant Enabling Chemo–and Biocatalysis and Related Reactions in Recyclable Water. *J. Am. Chem. Soc.* **2023**, *145*, 4266–4278.
3. Freiberg, K. M.; Kavthe, R. D.; Thomas, R. M.; Fialho, D. M.; Dee, P.; Scurria, M.; and Lipshutz, B. H. Direct formation of amide/peptide bonds from carboxylic acids: no traditional coupling reagents, 1–pot, and green. *Chem. Sci.* **2023**, *14,* 3462–3469.
4. Goosen, L. J.; Linder, C.; Rodríguez, N.; Lange, P. P. Biaryl and aryl ketone synthesis via Pd-catalyzed decarboxylative coupling of carboxylate salts with aryl triflates. *Chem. Eur. J.* **2009**, *15*, 9336–9349.
5. Rottländer, M.; Knochel, P. Palladium–catalyzed cross–coupling reactions with aryl nonaflates: a practical alternative to aryl triflates. *J. Org. Chem.* **1998**, *63*, 203–208.
6. McCann, S. D.; Reichert, E. C.; Arrechea, P. L.; Buchwald, S. L. Development of an Aryl Amination Catalyst with Broad Scope Guided by Consideration of Catalyst Stability. *J. Am. Chem. Soc.* **2020**, *142*, 15027−15037.
7. Ansari, T. N.; Taussat, A.; Clark, A. H.; Nachtegaal, M.; Plummer, S.; Gallou, F.; Handa, S. Insights on Bimetallic Micellar Nanocatalysis for Buchwald–Hartwig Aminations. *ACS Catal.* **2019**, *9*, 10389–10397.
8. A. Monti, J. L. Serrano, A. Prieto, M. C. Nicasio, *ACS Catal.* 2023, *13* , 10945–10952.

10. Analytical data

10.1. Analytical data for starting materials and intermediates

Benzo[d][1,3]dioxol–5–yl trifluoromethanesulfonate (43a)

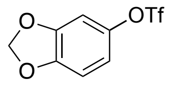

43a

Compound **43a** was made according to a previously reported literature method, with a slight modification.[4] To a solution of sesamol (2 mmol, 276 mg, 1.0 equiv) and Et_3N (4 mmol, 0.55 mL, 2.0 equiv) in CH_2Cl_2 (4 mL) at 0 °C, a solution of trifluoromethanesulfonic anhydride (2.4 mmol, 0.4 mL, 1.2 equiv) in CH_2Cl_2 (4 mL) was added dropwise. The mixture was then allowed to warm to rt and allowed to stir for 1 h. The mixture was diluted with CH_2Cl_2 and quenched with 3 M HCl (aq). The organic layer was washed with $NaHCO_3$ (aq, sat.) then brine. The solution was dried over anhydrous Na_2SO_4, filtered with an Et_2O wash, and the solvent was removed under reduced pressure to afford compound **43a** as a yellow oil. Yield: 96%, 521 mg; $\mathbf{R_f}$ = 0.40 (10% EtOAc/hexanes, UV).

^{1}H NMR (500 MHz, CDCl$_3$) δ 6.83 – 6.71 (m, 3H), 6.04 (s, 2H).

^{13}C NMR (101 MHz, CDCl$_3$) δ 148.7 (d, *J* = 1.9 Hz), 147.6 (d, *J* = 2.0 Hz), 143.6 (d, *J* = 2.0 Hz), 118.9 (q, *J* = 321.1 Hz), 114.5, 108.3 (d, *J* = 1.9 Hz), 103.5 (d, *J* = 1.9 Hz), 102.6 (d, *J* = 1.8 Hz), 46.9, 8.8.

^{19}F NMR (471 MHz, CDCl$_3$) δ –72.7.

HRMS (GC–EI$^+$): Calcd for $C_8H_5F_3O_5S$, $[M]^+$ 269.9810; found 269.9813.

4–(Benzyloxy)phenyl 1,1,2,2,3,3,4,4,4–nonafluorobutane–1–sulfonate (44a)

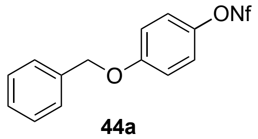

44a

Compound **44a** was made according to a previously reported literature method by Knochel and co–workers.[5] To a solution of 4–benzyloxy phenol (2 mmol, 400 mg, 1 equiv) and Et_3N (3 mmol, 0.42 ml, 1.5 equiv) in Et_2O (4 mL), a solution of perfluorobutane sulfonyl fluoride (3 mmol, 0.54 mL, 1.5 equiv) in Et_2O (4 mL) was added dropwise at 0 °C. The mixture was then allowed to warm to rt and allowed to stir for 1 h. The mixture was diluted with Et_2O and quenched with 3 M HCl (aq). The organic layer was washed with $NaHCO_3$ (aq, sat.) then brine. The solution was dried over anhydrous Na_2SO_4, filtered with an Et_2O wash, and the solvent was removed under reduced pressure to afford crude nonaflate. Chromatography conditions: 0–5% EtOAc/hexanes. Yield: 56%, 540.3 mg; light yellow oil; $\mathbf{R_f}$ = 0.57 (10% EtOAc/hexanes, UV, CAM stain).

^{1}H NMR (500 MHz, CDCl$_3$) δ 7.46 – 7.40 (m, 4H), 7.37 (ddt, *J* = 8.4, 5.6, 2.1 Hz, 1H), 7.24 – 7.19 (m, 2H), 7.03 – 6.98 (m, 2H), 5.08 (s, 2H).

^{13}C NMR (126 MHz, CDCl$_3$) δ 158.4, 143.6, 136.3, 128.9, 128.4, 127.6, 122.6, 116.1, 70.7.

^{19}F NMR (471 MHz, CDCl$_3$) δ –80.68 (tt, *J* = 9.8, 2.3 Hz), –109.00 (ddt, *J* = 16.1, 13.4, 2.4 Hz), –120.88 (dq, *J* = 9.4, 2.9 Hz), –125.57 – –126.01 (m).

HRMS (GC–EI$^+$): Calcd for $C_{17}H_{11}F_9O_4S$, $[M]^+$ 482.0234; found 482.0222.

7–Bromo–2–(piperazin–1–yl)quinoxaline (67b)

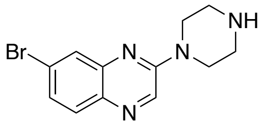

47b

^{1}H NMR (500 MHz, D_2O) δ 8.51 (d, J = 3.2 Hz, 1H), 7.43 (ddt, J = 9.0, 4.3, 1.9 Hz, 3H), 4.03 (t, J = 5.3 Hz, 4H), 3.47 (t, J = 5.3 Hz, 4H).

^{13}C NMR (126 MHz, D_2O) δ 150.9, 139.8, 136.4, 133.3, 128.9, 128.6, 126.9, 124.2, 42.9, 41.6.

HRMS (ESI$^+$): Calcd for $C_{12}H_{13}BrN_4$, $[M]^+$ 293.0402; found 293.0416.

10.2. Analytical data of coupled products

1–(3–((2,2–Difluorobenzo[d][1,3]dioxol–5–yl)amino)phenyl)ethan–1–one (1)

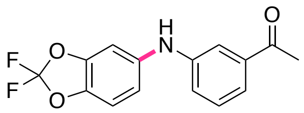

1

Product **1** was prepared according to General Procedure 1 at 60 °C for 18 h using 5–bromo–2,2–difluorobenzo[d][1,3]dioxole (59.2 mg, 34 µL, 0.250 mmol), 3–aminoacetophenone (51 mg, 0.375 mmol), 1250 ppm (0.125 mol %) $[Pd(crotyl)Cl]_2$ as catalyst, and 2 mol % *t*BuXPhos (2.2 mg) as ligand. Chromatography conditions: 10–20% EtOAc/hexanes. Yield: 91%, 66.3 mg; light yellow flaky solid; **R_f** = 0.44 (30% EtOAc/hexanes, UV, CAM stain).

^{1}H NMR (500 MHz, $CDCl_3$) δ 7.57 – 7.52 (m, 1H), 7.49 (dt, J = 7.6, 1.3 Hz, 1H), 7.35 (t, J = 7.8 Hz, 1H), 7.18 (ddd, J = 8.1, 2.4, 1.0 Hz, 1H), 6.97 (d, J = 8.5 Hz, 1H), 6.87 (d, J = 2.2 Hz, 1H), 6.76 (dd, J = 8.5, 2.3 Hz, 1H), 5.80 (s, 1H), 2.58 (s, 3H).

^{13}C NMR (126 MHz, $CDCl_3$) δ 198.2, 144.6, 144.1, 139.2, 138.9, 138.6, 131.9 (t, J = 254.9 Hz), 129.8, 121.4, 121.3, 116.2, 114.5, 110.1, 102.2, 26.9.

^{19}F NMR (471 MHz, $CDCl_3$) δ –50.1.

HRMS (ESI⁻): Calcd for $C_{15}H_{10}F_2NO_3$, $[M–H]^+$ 290.0628; found 290.0624.

Methyl 6–((3–(trifluoromethyl)phenyl)amino)–2–naphthoate (2)

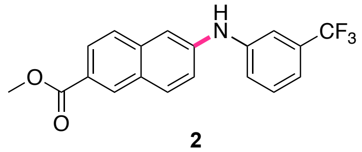

2

Product **2** was prepared according to General Procedure 1 at 45 °C for 4 h using methyl 6–bromo–2–naphthoate (66.2 mg, 0.250 mmol), 3–trifluoromethylaniline (60.4 mg, 47 µL, 0.375 mmol), 1250 ppm (0.125 mol %) $[Pd(crotyl)Cl]_2$ as catalyst, and 2 mol % *t*BuXPhos (2.2 mg) as ligand. Chromatography conditions: 10–12% EtOAc/hexanes. Yield: 94%, 81.2 mg; off white solid; $\mathbf{R_f}$ = 0.15 (10% EtOAc/hexanes, UV–fluorescent spot, CAM stain).

^{1}H NMR (400 MHz, $CDCl_3$) δ 8.51 (d, *J* = 1.7 Hz, 1H), 8.01 (dd, *J* = 8.6, 1.8 Hz, 1H), 7.87 (d, *J* = 8.8 Hz, 1H), 7.67 (d, *J* = 8.6 Hz, 1H), 7.46 – 7.35 (m, 4H), 7.27 (d, *J* = 2.3 Hz, 1H), 7.25 (d, *J* = 2.3 Hz, 1H), 6.14 (s, 1H), 3.97 (s, 3H).

^{13}C NMR (126 MHz, $CDCl_3$) δ 167.5, 142.8, 142.4, 137.1, 132.12 (q, *J* = 32.2 Hz), 131.2, 131.0, 130.2, 128.4, 126.7, 126.3, 125.4, 124.1 (q, *J* = 272.5 Hz), 121.6, 120.3, 118.6 (q, *J* = 3.8 Hz), 115.3 (q, *J* = 4.0 Hz), 111.2, 52.3.

^{19}F NMR (376 MHz, $CDCl_3$) δ –62.9.

HRMS (ESI⁻): Calcd for $C_{19}H_{13}F_3NO_2$, $[M–H]^+$ 344.0898; found 344.0887.

4–(5–(Indolin–1–yl)pyrimidin–2–yl)morpholine (3)

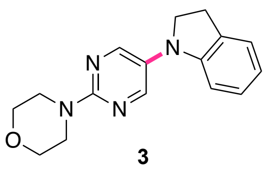

3

Product **3** was prepared according to General Procedure 1 at 60 °C for 18 h using 4–(5–bromopyrimidin–2–yl)morpholine (61 mg, 0.250 mmol), indoline (44.6 mg, 42 µL, 0.375 mmol), 1250 ppm (0.125 mol %) $[Pd(crotyl)Cl]_2$ as catalyst, and 2 mol % *t*BuXPhos (2.2 mg) as ligand. Chromatography conditions: 10–

20% EtOAc/hexanes. Yield: 95%, 67.1 mg; light yellow solid; $R_f = 0.11$ (10% EtOAc/hexanes, UV, CAM stain).

^{1}H NMR (400 MHz, $CDCl_3$) δ 8.37 (s, 2H), 7.16 (d, *J* = 7.2 Hz, 1H), 7.04 (t, *J* = 7.7 Hz, 1H), 6.78 – 6.65 (m, 2H), 3.83 (t, *J* = 8.4 Hz, 2H), 3.78 (q, *J* = 3.0 Hz, 8H), 3.14 (t, *J* = 8.4 Hz, 2H).

^{13}C NMR (101 MHz, $CDCl_3$) δ 158.7, 151.5, 148.8, 130.4, 130.3, 127.5, 125.2, 118.9, 107.3, 67.0, 53.7, 44.8, 28.6.

HRMS (GC–EI$^+$): Calcd for $C_{16}H_{19}N_4O$, $[M]^+$ 282.1481; found 282.1484.

***N*–(3–Fluoro–4–morpholinophenyl)–4–nitro–2–(trifluoromethyl)aniline (4)**

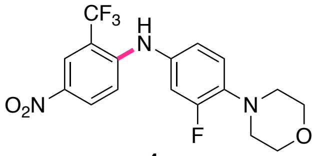

4

Product **4** was prepared according to General Procedure 1 at 60 °C for 16 h using 1–bromo–4–nitro–2–(trifluoromethyl)benzene (67.5 mg, 0.250 mmol), 3–fluoro–4–morpholinoaniline (73.5 mg, 0.375 mmol), 1750 ppm (0.175 mol %) $[Pd(crotyl)Cl]_2$ as catalyst, and 2 mol % *t*BuXPhos (2.2 mg) as ligand. Chromatography conditions: 10–20% EtOAc/hexanes. Yield: 83%, 80 mg; orange solid; $R_f = 0.18$ (20% EtOAc/hexanes, UV, CAM stain).

^{1}H NMR (400 MHz, $CDCl_3$) δ 8.47 (d, *J* = 2.6 Hz, 1H), 8.17 (dd, *J* = 9.3, 2.6 Hz, 1H), 7.04 – 6.93 (m, 4H), 6.55 (s, 1H), 3.93 – 3.86 (m, 4H), 3.15 – 3.08 (m, 4H).

^{13}C NMR (126 MHz, $CDCl_3$) δ 156.8, 154.8, 148.5, 138.80 (d, *J* = 8.4 Hz), 138.5, 132.4 (d, *J* = 9.8 Hz), 128.7, 124.1 (q, *J* = 5.7 Hz), 123.6 (q, *J* = 272.6 Hz), 121.2 (d, *J* = 3.2 Hz), 119.5 (d, *J* = 4.3 Hz), 114.0, 113.5 (d, *J* = 22.6 Hz), 66.9, 50.9, 50.8.

^{19}F NMR (376 MHz, $CDCl_3$) δ –62.6, –119.3.

HRMS (ESI$^-$): Calcd for $C_{17}H_{14}F_4N_3O_3$, $[M–H]^+$ 384.0971; found 384.0954.

1–(6–((2–Phenoxyphenyl)amino)pyridin–2–yl)ethan–1–one (5)

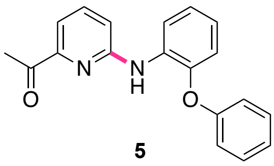

5

Product **5** was prepared according to General Procedure 1 at 60 °C for 18 h using 2–acetyl–6–bromopyridine (50 mg, 0.250 mmol), 3–phenoxyaniline (69.4 mg, 0.375 mmol), 1250 ppm (0.125 mol %) [Pd(crotyl)Cl]$_2$ as catalyst, and 2 mol % *t*BuXPhos (2.2 mg) as ligand. Chromatography conditions: 10–30% EtOAc/hexanes. Yield: 93%, 71 mg; tan solid; R_f = 0.21 (10% EtOAc/hexanes, UV, CAM stain).

^{1}H NMR (400 MHz, CDCl$_3$) δ 8.48 (d, J = 8.4 Hz, 1H), 7.62 (t, J = 7.8 Hz, 1H), 7.52 (d, J = 7.4 Hz, 1H), 7.36 (t, J = 7.8 Hz, 2H), 7.20 – 7.10 (m, 3H), 7.06 (d, J = 8.0 Hz, 2H), 6.97 – 6.90 (m, 3H), 2.73 (s, 3H).

^{13}C NMR (101 MHz, CDCl$_3$) δ 200.4, 157.0, 154.6, 151.9, 145.8, 138.2, 132.4, 130.1, 124.1, 123.8, 122.0, 119.4, 118.6, 118.5, 114.5, 113.5, 26.4.

HRMS (ESI$^+$): Calcd for $C_{19}H_{17}N_2O_2$, $[M+H]^+$ 305.1290; found 305.1297.

***N*–(4–Methoxy–3–nitrophenyl)–4–(pyrrolidin–1–yl)–2–(trifluoromethyl)aniline (6)**

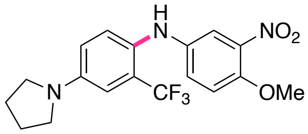

6

Product **6** was prepared according to General Procedure 1 and 4 (5 mmol scale) at 60 °C for 4 h using 1–(4–bromo–3–(trifluoromethyl)phenyl)pyrrolidine (73.5 mg, 0.250 mmol), 4–methoxy–3–nitroaniline (63 mg, 0.375 mmol), 1250 ppm (0.125 mol %) [Pd(crotyl)Cl]$_2$ as catalyst, and 2 mol % *t*BuXPhos (2.2 mg) as ligand. Chromatography conditions: 10–20% EtOAc/hexanes. Yield: 99%, 94.4 mg; dark orange solid; R_f = 0.19 (20% EtOAc/hexanes, UV, CAM stain).

^{1}H NMR (400 MHz, CDCl$_3$) δ 7.24 – 7.15 (m, 2H), 6.94 (d, J = 1.8 Hz, 2H), 6.78 (d, J = 2.9 Hz, 1H), 6.67 (dd, J = 8.8, 2.9 Hz, 1H), 5.37 (s, 1H), 3.89 (s, 3H), 3.31 (q, J = 4.8 Hz, 4H), 2.04 (dq, J = 9.2, 4.6 Hz, 4H).

^{13}C NMR (101 MHz, CDCl$_3$) δ 146.2, 145.0, 140.4, 140.2, 127.5, 127.2 (t, J = 136.2 Hz), 125.4 (q, J = 28.6 Hz), 122.9, 121.0, 115.5, 115.4, 111.3, 109.1 (q, J = 5.5 Hz), 57.2, 47.8, 25.5.

^{19}F NMR (376 MHz, $CDCl_3$) δ –61.4.

HRMS (ESI$^+$): Calcd for $C_{18}H_{18}F_3N_3O_3Na$, $[M+Na]^+$ 404.1198; found 404.1205.

3,4–Dimethyl–N–(3–(methylsulfonyl)phenyl)aniline (7)

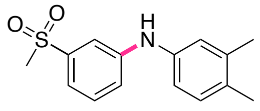

7

Product **7** was prepared according to General Procedure 1 at 60 °C for 18 h using 1–bromo–3–(methylsulfonyl)benzene (58.7 mg, 0.250 mmol), 3,4–dimethylaniline (45.4 mg, 0.375 mmol), 1250 ppm (0.125 mol %) $[Pd(crotyl)Cl]_2$ as catalyst, and 2 mol % *t*BuXPhos (2.2 mg) as ligand. Chromatography conditions: 20-40% EtOAc/hexanes. Yield: quantitative, 68.8 mg; off white solid; R_f = 0.18 (20% EtOAc/hexanes, UV, CAM stain).

^{1}H NMR (500 MHz, $CDCl_3$) δ 7.45 (t, *J* = 1.9 Hz, 1H), 7.39 – 7.31 (m, 2H), 7.18 (dt, *J* = 7.6, 2.1 Hz, 1H), 7.09 (d, *J* = 7.9 Hz, 1H), 6.96 – 6.87 (m, 2H), 3.74 (d, *J* = 1.2 Hz, 1H), 3.03 (d, *J* = 1.1 Hz, 3H), 2.24 (d, *J* = 3.4 Hz, 6H).

^{13}C NMR (126 MHz, $CDCl_3$) δ 145.9, 141.6, 138.6, 138.0, 131.9, 130.6, 130.3, 122.3, 120.0, 118.2, 117.5, 113.5, 63.7, 44.4, 20.0, 19.1.

HRMS (ESI$^+$): Calcd for $C_{15}H_{17}NO_2SNa$, $[M+Na]^+$ 298.0878; found 298.0878.

***N*–(3,4–Dimethoxyphenyl)–3–fluoro–6–methylpyridin–2–amine (8)**

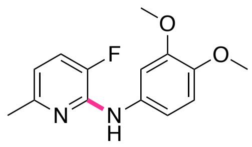

8

Product **8** was prepared according to General Procedure 1 at 60 °C for 16 h using 2–bromo–3–fluoro–6–methylpyridine (47.5 mg, 0.250 mmol), 3,4–dimethoxyaniline (57.4 mg, 0.375 mmol), 1250 ppm (0.125 mol %) $[Pd(crotyl)Cl]_2$ as catalyst, and 2 mol % *t*BuXPhos (2.2 mg) as ligand. Chromatography conditions:

10–20% EtOAc/hexanes. Yield: 96%, 63 mg; off white solid; R_f = 0.25 (20% EtOAc/hexanes, UV, CAM stain).

^{1}H NMR (500 MHz, $CDCl_3$) δ 7.63 (d, *J* = 2.5 Hz, 1H), 7.13 (dd, *J* = 11.1, 8.0 Hz, 1H), 7.03 (dd, *J* = 8.6, 2.5 Hz, 1H), 6.83 (d, *J* = 8.6 Hz, 1H), 6.51 (dd, *J* = 8.0, 3.0 Hz, 1H), 6.48 – 6.43 (m, 1H), 3.92 (s, 3H), 3.87 (s, 3H), 2.42 (s, 3H).

^{13}C NMR (126 MHz, $CDCl_3$) δ 151.40 (d, *J* = 5.9 Hz), 149.2, 146.3, 144.67 – 144.17 (m), 134.1, 121.2, 121.1, 113.0, 112.95 (d, *J* = 2.1 Hz)110.6, 104.3, 56.4, 55.9, 23.9.

^{19}F NMR (471 MHz, $CDCl_3$) δ –145.3, –145.3.

HRMS (ESI$^+$): Calcd for $C_{14}H_{16}FN_2O_2$, $[M+H]^+$ 263.1196; found 263.1192.

4–((4–((4–Methylpiperazin–1–yl)methyl)–3–(trifluoromethyl)phenyl)amino)–2–(trifluoromethyl)benzonitrile (9)

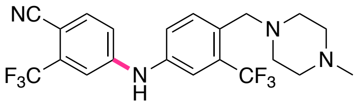

9

Product **9** was prepared according to General Procedure 1 at 60 °C for 24 h using 4–bromo–2–(trifluoromethyl)benzonitrile (62.5 mg, 0.250 mmol), 4–((4–methylpiperazin–1–yl)methyl)–3–(trifluoromethyl)aniline (102.5 mg, 0.375 mmol), 1250 ppm (0.125 mol %) $[Pd(crotyl)Cl]_2$ as catalyst, and 2 mol % *t*BuXPhos (2.2 mg) as ligand. Chromatography conditions: 2.5–7.5% MeOH/CH_2Cl_2, 0.5% Et_3N. Yield: 92%, 101.7 mg; tan solid; R_f = 0.27 (10% MeOH/CH_2Cl_2, UV, CAM stain).

^{1}H NMR (500 MHz, $CDCl_3$) δ 7.82 (d, *J* = 8.3 Hz, 1H), 7.65 (d, *J* = 8.5 Hz, 1H), 7.41 (d, *J* = 2.4 Hz, 1H), 7.35 (dd, *J* = 8.3, 2.4 Hz, 1H), 7.23 (d, *J* = 2.4 Hz, 1H), 7.11 (dd, *J* = 8.6, 2.4 Hz, 1H), 6.35 (s, 1H), 3.65 (s, 2H), 2.62 – 2.41 (m, 8H), 2.31 (s, 3H).

^{13}C NMR (126 MHz, $CDCl_3$) δ 147.6, 137.8, 136.4, 134.68 (q, *J* = 32.4 Hz), 134.4, 132.2, 130.22 (q, *J* = 30.7 Hz), 124.5, 123.4, 122.7, 121.2, 119.15 (q, *J* = 5.9 Hz), 117.8, 116.5, 116.3, 112.87 (q, *J* = 4.9 Hz), 99.18 (d, *J* = 2.2 Hz), 57.7, 55.2, 53.1, 46.0.

^{19}F NMR (471 MHz, $CDCl_3$) δ –59.7, –62.5.

HRMS (ESI⁺): Calcd for $C_{21}H_{21}F_6N_4$, $[M+H]^+$ 443.1671; found 443.1673.

3–(Benzyloxy)–6–((*t*–butyldimethyl silyl) oxy)–*N*–(3–fluoro–4–morpholinophenyl)naphthalen–2–amine (10):

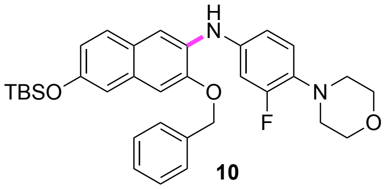

10

Product **10** was prepared according to General Procedure 1 at 70 °C for 24 h using ((7–(benzyloxy)–6–bromonaphthalen–2–yl)oxy)(*t*–butyl)dimethylsilane (110.86 mg, 0.25 mmol), 3–fluoro–4–morpholino-aniline (72 mg, 0.375 mmol), 1750 ppm (0.175 mol %) $[Pd(crotyl)Cl]_2$ as catalyst, and 2 mol % *t*BuXPhos (2.2 mg) as ligand. Chromatography conditions: 10 % EtOAc/hexanes. Yield: 97%, 135.3 mg; yellow solid; R_f = 0.40 (30% EtOAc/hexanes, UV, CAM stain).

^{1}H NMR (400 MHz, $CDCl_3$) δ 7.51 – 7.33 (m, 7H), 7.10 (s, 1H), 7.07 – 6.81 (m, 5H), 6.22 (s, 1H), 5.22 (s, 2H), 3.91 (s, 4H), 3.10 (s, 4H), 1.02 (s, 9H), 0.23 (s, 6H).

^{13}C NMR (101 MHz, $CDCl_3$) δ 157.5, 155.1, 152.0, 148.3, 138.2, 136.5, 134.4, 132.2, 129.8, 128.8, 128.4, 127.9, 127.3, 125.1, 120.1, 119.7, 115.7, 114.3, 108.8, 108.5, 108.2, 106.0, 70.7, 67.1, 51.5, 51.5, 25.8, 25.8, 25.8, 18.3, –4.2, –4.3.

HRMS (ESI +): Calcd for $C_{33}H_{40}FN_2O_3Si$, $[M+H]^+$ 559.2792; found 559.2775.

2,4–Dimethoxy–*N*–(4–methoxy–3–nitrophenyl)pyrimidin–5–amine (11):

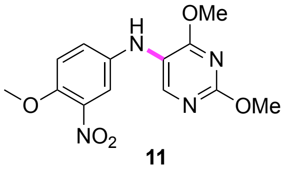

11

Product **11** was prepared according to General Procedure 1 at 70 °C for 24 h using 5–bromo–2,4–dimethoxypyrimidine (54.76 mg, 0.25 mmol), 4–methoxy–3–nitroaniline (63 mg, 0.375 mmol), 1750 ppm (0.175 mol %) $[Pd(crotyl)Cl]_2$ as catalyst, and 2 mol % *t*BuXPhos (2.2 mg) as ligand. Chromatography conditions: 0.5% MeOH/CH_2Cl_2. Yield: 79%, 60.8 mg; orange solid; R_f = 0.35 (3% MeOH/CH_2Cl_2, UV, CAM stain).

^{1}H NMR (400 MHz, CDCl$_3$) δ 8.10 (s, 1H), 7.42 (d, *J* = 2.9 Hz, 1H), 7.14 (dd, *J* = 8.9, 2.9 Hz, 1H), 7.02 (d, *J* = 9.0 Hz, 1H), 5.39 (s, 1H), 4.06 (s, 3H), 4.00 (s, 3H), 3.93 (s, 3H).

^{13}C NMR (126 MHz, CDCl$_3$) δ 163.5, 160.4, 147.4, 146.2, 134.0, 137.0, 122.6, 119.7, 115.3, 113.5, 57.1, 54.9, 54.4.

HRMS (ESI$^+$): Calcd for $C_{13}H_{15}N_4O_5$, $[M+H]^+$ 307.1042; found 307.1042.

1–(4–((4–((3–(Trifluoromethyl)phenyl)amino)phenyl)sulfonyl)piperazin–1–yl) ethan–1–one (12):

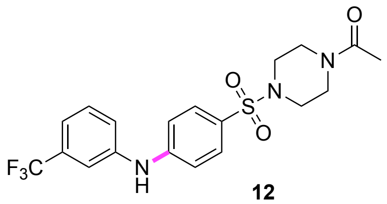

12

Product **12** was prepared according to General Procedure 1 at 60 °C for 18 h using 1–(4–((4–bromophenyl)sulfonyl)piperazin–1–yl)ethan–1–one (86.8 mg, 0.25 mmol), 3–(trifluoromethyl)aniline (60.42 mg, 0.375 mmol), 1750 ppm (0.175 mol %) [Pd(crotyl)Cl]$_2$ as catalyst, and 2 mol % *t*BuXPhos (2.2 mg) as ligand. Chromatography conditions: 0.5–1% MeOH/CH_2Cl_2. Yield: 95%, 101.6 mg; white solid; R_f = 0.25 (2% MeOH/CH_2Cl_2, UV, CAM stain).

^{1}H NMR (400 MHz, CDCl$_3$) δ 7.65 – 7.55 (m, 2H), 7.47 (t, *J* = 7.9 Hz, 1H), 7.41 (d, *J* = 1.9 Hz, 1H), 7.35 (t, *J* = 8.3 Hz, 2H), 7.13 – 7.00 (m, 2H), 3.80 – 3.42 (m, 4H), 3.01 (s, 4H), 2.05 (s, 3H).

^{13}C NMR (126 MHz, CDCl$_3$) δ 169.0, 169.0, 147.7, 147.7, 141.1, 141.1, 132.0 (q, *J* = 32.76 Hz), 132.0 (q, *J* = 32.76 Hz), 130.2, 130.9, 129.9, 129.8, 127.1, 125.4, 125.3, 125.3, 124.9, 123.2, 123.2, 122.7, 120.6, 119.9, 119.8, 119.7, 116.9, 115.3, 115.0, 46.2, 45.8, 45.7, 29.7, 21.3.

^{19}F NMR (376 MHz, CDCl$_3$) δ –62.79.

HRMS (ESI$^+$): Calcd for $C_{19}H_{21}F_3N_3O_3S$, $[M+H]^+$ 428.1256; found 428.1238.

***N*–(4–(Methylthio)phenyl)–3–(piperidin–1–ylmethyl)aniline (13):**

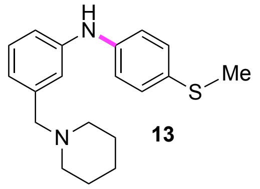

13

Product **13** was prepared according to General Procedure 1 at 70 °C for 24 h using (4–bromophenyl)(methyl)sulfane (50.77 mg, 0.25 mmol), 3–(piperidin–1–ylmethyl)aniline (56.25 mg, 0.375 mmol), 1750 ppm (0.175 mol %) $[Pd(crotyl)Cl]_2$ as catalyst, and 2 mol % *t*BuXPhos (2.2 mg) as ligand. Chromatography conditions: 0.5–2% $MeOH/CH_2Cl_2$. Yield: 69%, 53.7 mg; off white solid; R_f = 0.45 (5% $MeOH/CH_2Cl_2$, UV, CAM stain).

^{1}H NMR (400 MHz, $CDCl_3$) δ 7.26 – 7.16 (m, 3H), 7.07 (t, *J* = 2.0 Hz, 1H), 7.05 – 6.94 (m, 3H), 6.88 (dt, *J* = 7.5, 1.3 Hz, 1H), 5.73 (s, 1H), 3.50 (s, 2H), 2.46 (s, 7H), 1.74 – 1.55 (m, 4H), 1.45 (p, *J* = 6.1 Hz, 2H).

^{13}C NMR (126 MHz, $CDCl_3$) δ 143.2, 141.4, 138.3, 130.0, 129.3, 129.1, 129.1, 122.3, 118.9, 118.7, 116.4, 63.4, 54.3, 25.5, 24.1, 18.0.

HRMS (ESI$^+$): Calcd for $C_{19}H_{25}N_2S$, $[M+H]^+$ 313.1738; found 313.1734.

1–(5–((3–Acetylphenyl)amino)thiophen–2–yl)ethan–1–one (14):

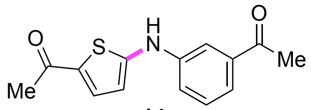

14

Product **14** was prepared according to General Procedure 1 at 60 °C for 18 h using 1–(5–bromothiophen–2–yl)ethan–1–one (51.26 mg, 0.25 mmol), 1–(3–aminophenyl)ethan–1–one (50.68 mg, 0.375 mmol), 1750 ppm (0.175 mol %) $[Pd(crotyl)Cl]_2$ as catalyst, and 2 mol % *t*BuXPhos (2.2 mg) as ligand. Chromatography conditions: 10–30% EtOAc/hexanes. Yield: 89%, 57.6 mg; yellow solid; R_f = 0.45 (50%EtOAc/hexanes, UV, CAM stain).

^{1}H NMR (400 MHz, DMSO–d_6) δ 10.04 (s, 1H), 7.81 – 7.68 (m, 2H), 7.58 (dt, *J* = 7.4, 1.5 Hz, 1H), 7.55 – 7.40 (m, 2H), 6.58 (d, *J* = 4.3 Hz, 1H), 2.58 (s, 3H), 2.40 (s, 3H).

^{13}C NMR (126 MHz, DMSO–d_6) δ 197.6, 188.6, 156.4, 142.9, 137.9, 135.3, 135.0, 135.0, 130.0, 128.6, 121.6, 121.1, 115.2, 109.5, 26.7, 25.3.

HRMS (ESI$^+$): Calcd for $C_{14}H_{14}NO_2S$, $[M+H]^+$ 260.0745; found 260.0757.

N–(3,4–Dimethylphenyl)–3–(1–methyl–1H–pyrazol–4–yl)quinoxalin–6–amine (15):

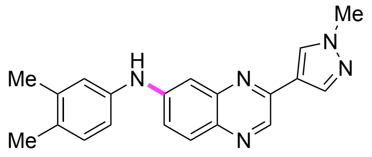

15

Product **15** was prepared according to General Procedure 1 at 70 °C for 24 h using 7–bromo–2–(1–methyl–1H–pyrazol–4–yl)quinoxaline (72.28 mg, 0.25 mmol), 3,4–dimethylaniline (45.45 mg, 0.375 mmol), 1750 ppm (0.175 mol %) $[Pd(crotyl)Cl]_2$ as catalyst, and 2 mol % *t*BuXPhos (2.2 mg) as ligand. Chromatography conditions: 0.5–2 % $MeOH/CH_2Cl_2$. Yield: 95%, 78.18 mg; yellow solid; R_f = 0.30 (5% $MeOH/CH_2Cl_2$, UV, CAM stain).

^{1}H NMR (400 MHz, $CDCl_3$) δ 8.78 (s, 1H), 8.24 (s, 1H), 8.13 (s, 1H), 7.85 (d, J = 9.0 Hz, 1H), 7.51 – 7.45 (m, 1H), 7.30 (dd, *J* = 9.1, 2.6 Hz, 1H), 7.13 (d, *J* = 7.9 Hz, 1H), 7.08 – 7.00 (m, 2H), 6.14 (s, 1H), 4.00 (s, 3H), 2.27 (d, *J* = 4.9 Hz, 6H).

^{13}C NMR (101 MHz, $CDCl_3$) δ 147.0, 146.7, 144.4, 139.2, 138.6, 138.2, 137.9, 136.8, 132.0, 130.6, 130.0, 129.8, 122.8, 121.5, 121.4, 118.8, 107.6, 39.3, 20.0, 19.2.

HRMS (ESI$^+$): Calcd for $C_{20}H_{20}N_5$, $[M+H]^+$ 330.1719; found 330.1716.

(4–((3,5–Dimethoxyphenyl) amino)phenyl) (pyrrolidin–1–yl) methanone (16):

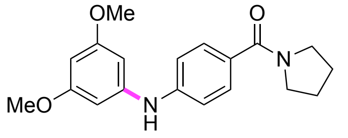

16

Product **16** was prepared according to General Procedure 1 at 70 °C for 16 h using (4–bromophenyl)(pyrrolidin–1–yl)methanone (63.25 mg, 0.25 mmol), 3,5–dimethoxyaniline (57.45 mg, 0.375 mmol), 1750 ppm (0.175 mol %) $[Pd(crotyl)Cl]_2$ as catalyst, and 2 mol % *t*BuXPhos (2.2 mg) as ligand. Chromatography conditions: 20% EtOAc/hexanes. Yield: >99%, 81.5 mg; off white solid; R_f = 0.50 (30% EtOAc/hexanes, UV, CAM stain).

^{1}H NMR (400 MHz, $CDCl_3$) δ 7.52 – 7.43 (m, 2H), 7.08 – 7.02 (m, 2H), 6.29 (d, *J* = 2.1 Hz, 2H), 6.12 (t, *J* = 2.1 Hz, 1H), 3.77 (s, 6H), 3.58 (q, *J* = 4.8 Hz, 4H), 1.99 – 1.85 (m, 4H).

^{13}C NMR (126 MHz, CDCl$_3$) δ 169.6, 161.6, 144.9, 144.1, 129.1, 128.6, 116.7, 116.5, 97.0, 93.9, 55.4, 49.9, 46.5, 26.5, 24.5.

HRMS (ESI$^+$): Calcd for $C_{19}H_{23}N_2O_3$, $[M+H]^+$ 327.1709; found 327.1707.

3–((1S, 3S)–Adamantan–1–yl)–4–methoxy–*N*–(3–methoxyphenyl)aniline (17):

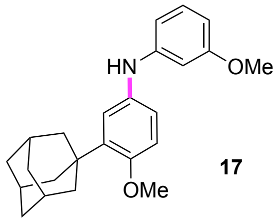

17

Product **17** was prepared according to General Procedure 1 at 70 °C for 24 h using (1S, 3S)–1–(5–bromo–2–methoxyphenyl)adamantane (80.32 mg, 0.25 mmol), 3–methoxyaniline (46.18 mg, 0.375 mmol), 1750 ppm (0.175 mol %) [Pd(crotyl)Cl]$_2$ as catalyst, and 2 mol % *t*BuXPhos (2.2 mg) as ligand. Chromatography conditions: 5% EtOAc/hexanes. Yield: 60%, 54.2 mg; white solid; R_f = 0.40 (10 % EtOAc/hexanes, UV, CAM stain).

^{1}H NMR (400 MHz, CDCl$_3$) δ 7.17 – 7.02 (m, 3H), 6.81 (d, *J* = 8.5 Hz, 1H), 6.61 (d, J = 9.4 Hz, 2H), 6.47 (d, *J* = 8.2 Hz, 1H), 3.81 (d, J = 7.7 Hz, 4H), 3.75 (s, 3H), 2.05 (d, *J* = 4.9 Hz, 11H), 1.76 (s, 8H).

^{13}C NMR (101 MHz, CDCl$_3$) δ 160.8, 154.9, 147.0, 139.7, 135.0, 130.1, 120.9, 119.3, 112.6, 108.2, 104.6, 101.0, 55.5, 55.2, 40.7, 37.2, 37.1, 29.2.

HRMS (ESI$^+$): Calcd for $C_{24}H_{30}NO_2$, $[M+H]^+$ 364.2277; found 364.2291.

***N*–(1–(2,3–Dimethoxyphenyl)naphthalen–2–yl)–1,2–dihydroacenaphthylen–5–amine (18):**

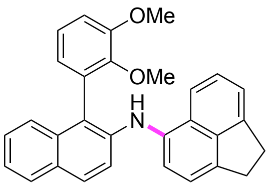

18

Product **18** was prepared according to General Procedure 1 at 70 °C for 24 h using 5–bromo–1,2–dihydroacenaphthylene (29.13 mg, 0.125 mmol), 1–(2,3–dimethoxyphenyl)naphthalen–2–amine (56.25

mg, 0.375 mmol), 1750 (0.175 mol %) $[Pd(crotyl)Cl]_2$ as catalyst, and 2 mol % *t*BuXPhos (1.1 mg) as ligand. Chromatography conditions: 5 –10 % EtOAc/hexanes. Yield: 96%, 52.0 mg; off white solid; R_f = 0.35 (10% EtOAc/hexanes, UV, CAM stain).

^{1}H NMR (400 MHz, $CDCl_3$) δ 7.75 (dd, *J* = 7.8, 1.6 Hz, 1H), 7.68 (d, *J* = 9.0 Hz, 1H), 7.50 (d, *J* = 8.3 Hz, 1H), 7.41 – 7.26 (m, 8H), 7.19 (d, *J* = 7.3 Hz, 1H), 6.76 – 6.63 (m, 2H), 3.89 (s, 3H), 3.73 (s, 3H), 3.49 – 3.24 (m, 4H).

^{13}C NMR (101 MHz, $CDCl_3$) δ 161.1, 159., 146.44, 142.0, 140.9, 140.5, 135.6, 134.4, 133.5, 129.0, 128.3, 128.1, 127.4, 126.9, 126.2, 124.9, 122.7, 119.8, 119.6, 119.3, 119.0, 118.2, 118.0, 117.5, 105.6, 99.6, 55.8, 55.6, 31.0, 29.9.

HRMS (ESI$^+$): Calcd for $C_{30}H_{26}NO_2$, $[M+H]^+$ 432.1964; found 432.1962.

***N*-(*p*-Tolyl)-1-tosyl-1*H*-pyrrolo[2,3-*b*]pyridin-4-amine (19):**

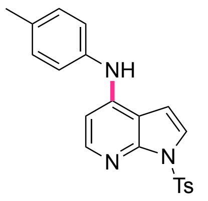

19

Product **19** was prepared according to General Procedure 1 at 60 °C for 2 h using 4-bromo-1-tosyl-1*H*-pyrrolo[2,3-*b*]pyridine (87.8 mg, 0.25 mmol), *p*-toluidine (40.2 mg, 0.375 mmol), 1250 (0.125 mol %) $[Pd(crotyl)Cl]_2$ as catalyst, and 2 mol % *t*BuXPhos (2.2 mg) as ligand. Chromatography conditions: 15 –25 % EtOAc/hexanes. Yield: 85%, 80.2 mg; off white solid; R_f = 0.23 (25% EtOAc/hexanes, UV, CAM stain).

^{1}H NMR (400 MHz, $CDCl_3$) δ 8.12 (d, *J* = 5.6 Hz, 1H), 8.07 (d, *J* = 8.2 Hz, 2H), 7.55 (d, *J* = 4.1 Hz, 1H), 7.28 (d, *J* = 3.0 Hz, 2H), 7.18 (d, *J* = 8.1 Hz, 2H), 7.11 (d, *J* = 8.1 Hz, 2H), 6.70 (d, *J* = 5.6 Hz, 1H), 6.45 (d, *J* = 4.1 Hz, 1H), 6.14 (s, 1H), 2.37 (d, *J* = 5.0 Hz, 6H).

^{13}C NMR (126 MHz, $CDCl_3$) δ 148.7, 146.4, 145.4, 145.1, 136.8, 135.7, 134.7, 130.2, 129.7, 128.2, 123.5, 122.9, 110.7, 102.3, 102.0, 21.8, 21.1.

HRMS (GC–EI): Calcd for $C_{21}H_{19}N_3O_2S$, $[M]^+$ 377.1198; found 377.1194.

***t*-Butyl 4-(7-((3-(trifluoromethyl)phenyl)amino)quinoxalin-2-yl)piperazine-1-carboxylate (20):**

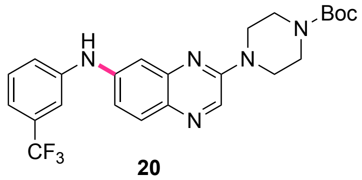

20

Product **20** was prepared according to General Procedure 1 at 60 °C for 2 h using *t*–butyl 4–(7–bromoquinoxalin–2–yl)piperazine–1–carboxylate (100 mg, 0.25 mmol), 3-trifluoromethylaniline (60.4 mg, 47 µL, 0.375 mmol), 1250 (0.125 mol %) [Pd(crotyl)Cl]$_2$ as catalyst, and 2 mol % *t*BuXPhos (2.2 mg) as ligand. Chromatography conditions: 2–5% MeOH/CH_2Cl_2. Yield: 97%, 115 mg; yellow solid; R_f = 0.12 (2% MeOH/CH_2Cl_2, UV, CAM stain).

^{1}H NMR (400 MHz, $CDCl_3$) δ 8.37 (s, 1H), 7.77 (d, *J* = 8.8 Hz, 1H), 7.40 (dd, *J* = 6.2, 4.6 Hz, 3H), 7.25 (s, 2H), 7.10 (dd, *J* = 8.8, 2.6 Hz, 1H), 6.27 (d, *J* = 17.0 Hz, 1H), 3.74 (dd, *J* = 6.7, 3.9 Hz, 4H), 3.59 (dd, *J* = 6.7, 3.8 Hz, 4H), 1.49 (s, 9H).

^{13}C NMR (126 MHz, $CDCl_3$) δ 154.9, 152.7, 144.3, 143.3, 142.6, 133.2, 132.8, 132.1 (q, *J* = 32.2 Hz), 130.1, 130.0, 124.1 (q, *J* = 272.5 Hz), 122.0, 118.7 (d, *J* = 3.9 Hz), 117.8, 115.7 (q, *J* = 3.8 Hz), 109.7 (d, *J* = 2.4 Hz), 80.4, 44.7, 43.7, 43.0, 28.5.

^{19}F NMR (376 MHz, $CDCl_3$) δ -62.8.

HRMS (ESI$^+$): Calcd for $C_{24}H_{26}F_3N_5O_2Na$, $[M+Na]^+$ 496.1936; found 496.1944.

***N*-(4-Methoxy-3-nitrophenyl)-1-(triisopropylsilyl)-1*H*-indol-5-amine (21):**

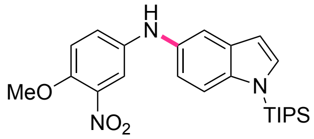

21

Product **21** was prepared according to General Procedure 1 at 60 °C for 2 h using 5-bromo-1-(triisopropylsilyl)-1*H*-indole (88.1 mg, 0.25 mmol), 3-methoxy-4-nitroaniline (63 mg, 0.375 mmol), 1250 (0.125 mol %) [Pd(crotyl)Cl]$_2$ as catalyst, and 2 mol % *t*BuXPhos (2.2 mg) as ligand. Chromatography

conditions: 15 –25 % EtOAc/hexanes. Yield: 73%, 80.7 mg; orange solid; R_f = 0.34 (25% EtOAc/hexanes, UV, CAM stain).

^{1}H NMR (400 MHz, $CDCl_3$) δ 7.41 – 7.35 (m, 2H), 7.20 – 7.18 (m, 1H), 7.06 (dd, *J* = 9.0, 2.9 Hz, 1H), 6.89 (d, *J* = 9.0 Hz, 1H), 6.83 (dd, *J* = 8.8, 2.3 Hz, 1H), 6.48 (dd, *J* = 3.2, 0.8 Hz, 1H), 5.48 (s, 1H), 3.83 (s, 3H), 1.62 (p, *J* = 7.6 Hz, 3H), 1.09 (d, *J* = 7.5 Hz, 18H).

^{13}C NMR (126 MHz, $CDCl_3$) δ 146.3, 140.4, 140.1, 137.9, 134.7, 132.5, 132.4, 121.7, 116.7, 115.5, 114.8, 112.5, 112.3, 104.6, 57.4, 18.3, 12.9.

HRMS (GC–EI): Calcd for $C_{24}H_{33}N_3O_3Si$, $[M]^+$ 439.2291; found 439.2311.

***t*-Butyl (*S*)-4-(4-methoxy-3-((3-(trifluoromethoxy)phenyl)amino)phenyl)-2,2-dimethyloxazolidine-3-carboxylate (22):**

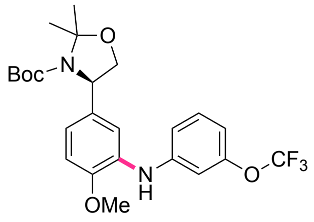

22

Product **22** was prepared according to General Procedure 1 at 60 °C for 2 h using *t*-butyl (*S*)-4-(3-bromo-4-methoxyphenyl)-2,2-dimethyloxazolidine-3-carboxylate (96.5 mg, 0.25 mmol), 3-trifluoromethoxy-aniline (66.4 mg, 0.375 mmol), 1250 (0.125 mol %) $[Pd(crotyl)Cl]_2$ as catalyst, and 2 mol % *t*BuXPhos (2.2 mg) as ligand. Note: The product spot co-elutes with the amine, so a slow column is advised. Chromatography conditions: 2.5% EtOAc/hexanes until the excess amine is isolated, then flush with 15% EtOAc/hexanes. Yield: 95%, 115 mg; colorless to faint yellow oil; R_f = 0.10 (5% EtOAc/hexanes, UV, CAM stain).

^{1}H NMR (500 MHz, $CDCl_3$) δ 7.23 (d, *J* = 8.0 Hz, 1H), 7.00 (dd, *J* = 8.3, 2.2 Hz, 1H), 6.95 (s, 1H), 6.84 (s, 2H), 6.74 (d, *J* = 8.1 Hz, 1H), 6.20 (s, 1H), 4.25 (dd, *J* = 9.0, 6.7 Hz, 1H), 3.92 – 3.79 (m, 4H), 1.73 (s, 3H), 1.57 (t, *J* = 5.4 Hz, 4H), 1.46 (s, 4H), 1.24 (s, 5H).

^{13}C NMR (126 MHz, CDCl$_3$) δ 152.4, 152.2, 150.3, 150.3, 148.0, 144.6, 135.3, 134.6, 131.6, 130.3, 120.6 (q, *J* = 256.8 Hz), 119.4, 119.2, 115.9 (d, *J* = 8.3 Hz), 113.8 (d, *J* = 32.8 Hz), 112.5 (d, *J* = 47.4 Hz), 110.5 (d, *J* = 32.6 Hz), 109.9 (d, *J* = 17.0 Hz), 94.6, 94.1, 80.4, 79.7, 70.9, 70.5, 60.9, 60.9, 55.7, 28.4, 28.3, 27.0, 26.1, 24.7, 23.7.

^{19}F NMR (471 MHz, CDCl$_3$) δ -57.6.

HRMS (GC–EI): Calcd for $C_{24}H_{29}F_3N_2O_5$, $[M]^+$ 482.2029; found 482.2043.

4-((2-Morpholinophenyl)amino)phenyl 3-(trifluoromethyl)benzoate (23):

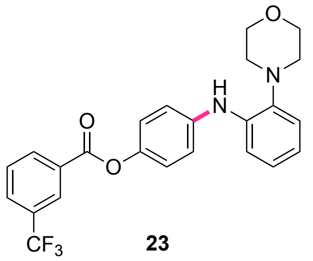

23

Product **23** was prepared according to General Procedure 1 at 60 °C for 2 h using 4-bromophenyl 3-(trifluoromethyl)benzoate (86.3 mg, 0.25 mmol), 2-morpholinoaniline (67 mg, 0.375 mmol), 1750 (0.175 mol %) [Pd(crotyl)Cl]$_2$ as catalyst, and 2 mol % *t*BuXPhos (2.2 mg) as ligand. Note: Chromatography conditions: 15–20% EtOAc/hexanes Yield: 71%, 78 mg; yellow oil; R_f = 0.21 (15% EtOAc/hexanes, UV, CAM stain).

^{1}H NMR (500 MHz, CDCl$_3$) δ 8.48 (s, 1H), 8.40 (d, *J* = 7.8 Hz, 1H), 7.90 (d, *J* = 7.8 Hz, 1H), 7.67 (t, *J* = 7.8 Hz, 1H), 7.32 (ddd, *J* = 8.1, 5.7, 1.8 Hz, 2H), 7.23 – 7.19 (m, 2H), 7.18 – 7.14 (m, 2H), 7.12 (dd, *J* = 7.8, 1.5 Hz, 1H), 7.06 (td, *J* = 7.7, 1.5 Hz, 1H), 6.90 (td, *J* = 7.6, 1.4 Hz, 1H), 6.62 (s, 1H), 3.87 (dd, *J* = 5.8, 3.3 Hz, 4H), 2.95 (t, *J* = 4.6 Hz, 4H).

^{13}C NMR (126 MHz, CDCl$_3$) δ 164.5, 154.9, 144.7, 141.0, 140.8, 138.3, 133.5, 132.6, 131.5 (q, *J* = 33.1 Hz), 130.7, 130.2 (q, *J* = 3.6 Hz), 129.4, 127.2 (q, *J* = 3.9 Hz), 125.0, 123.8 (q, *J* = 272.6 Hz), 122.5, 120.5, 120.4, 119.5, 117.3, 114.7, 112.9, 67.8, 52.1.

^{19}F NMR (471 MHz, CDCl$_3$) δ -62.8.

1–(Dibenzo[b,d]thiophen–4–yl)indoline (24)

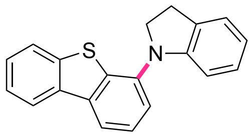

24

Product **24** was prepared according to General Procedure 1 at 70 °C for 24 h using 4–iododibenzo[b,d]thiophene (77.5 mg, 0.25 mmol), indoline (44.6 mg, 42 µL, 0.375 mmol), 1250 ppm (0.125 mol %) [Pd(crotyl)Cl]$_2$ as catalyst, and 2 mol % *t*BuXPhos (2.2 mg) as ligand. Chromatography conditions: 100% hexanes. Yield: 47%, 35 mg; colorless oil; R_f = 0.45 (1% EtOAc/hexanes, UV, CAM stain).

^{1}H NMR (500 MHz, CDCl$_3$) δ 8.25 – 8.17 (m, 1H), 8.07 – 8.00 (m, 1H), 7.92 – 7.84 (m, 1H), 7.50 (td, *J* = 5.5, 2.3 Hz, 4H), 7.28 (d, *J* = 2.5 Hz, 1H), 7.07 (t, *J* = 7.7 Hz, 1H), 6.82 (t, *J* = 7.3 Hz, 1H), 6.59 (d, *J* = 7.9 Hz, 1H), 4.07 (t, *J* = 8.3 Hz, 2H), 3.27 (t, *J* = 8.3 Hz, 2H).

^{13}C NMR (126 MHz, CDCl$_3$) δ 149.1, 140.7, 139.6, 137.6, 136.3, 136.0, 130.8, 127.1, 127.0, 125.7, 125.0, 124.5, 122.9, 121.9, 120.4, 119.2, 118.3, 109.9, 54.1, 29.2.

HRMS (GC–EI$^+$): Calcd for $C_{20}H_{15}NS$, $[M]^+$ 301.0925; found 301.0923.

4–Fluoro–N–(4–(morpholinosulfonyl)phenyl)aniline (30)

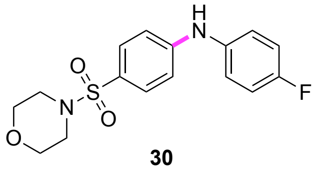

30

Product **30** was prepared according to General Procedure 1 (see SI, section S5 for recycling studies) at 60 °C for 2 h using 4–((4–bromophenyl)sulfonyl)morpholine (76.5 mg, 0.250 mmol), 4–fluoroaniline (41.7 mg, 36 µL, 0.375 mmol), 500 ppm (0.05 mol %) [Pd(crotyl)Cl]$_2$ as catalyst, and 2 mol % *t*BuXPhos (2.2 mg) as ligand. Chromatography conditions: 25–40% EtOAc/hexanes. Yield: 93%, 78 mg; tan solid; R_f = 0.17 (25% EtOAc/hexanes, UV, CAM stain).

^{1}H NMR (500 MHz, CDCl$_3$) δ 7.58 – 7.52 (m, 2H), 7.20 – 7.12 (m, 2H), 7.10 – 7.02 (m, 2H), 6.95 – 6.88 (m, 2H), 6.12 (s, 1H), 3.76 – 3.70 (m, 4H), 2.98 (dd, *J* = 5.7, 3.8 Hz, 4H).

^{13}C NMR (126 MHz, CDCl$_3$) δ 160.7, 158.7, 149.3, 136.1 (d, *J* = 3.0 Hz), 130.1, 124.3, 124.2, 124.1, 116.6, 116.4, 114.0, 66.2, 46.1.

^{19}F NMR (471 MHz, CDCl$_3$) δ –117.8.

HRMS (ESI$^+$): Calcd for $C_{16}H_{17}FN_2O_3SNa$, $[M+H]^+$ 359.0842; found 359.0834.

***t*–Butyl 5–((2–chloro–4–((2–(dimethylamino)ethyl)carbamoyl)–5–methoxyphenyl)amino)–1H–indole–1–carboxylate (31):**

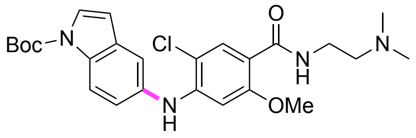

31

Product **31** was prepared according to General Procedure 1 at 70 °C for 24 h using *t*–butyl 5–bromo–1H–indole–1–carboxylate (74.04 mg, 0.25 mmol), 4–amino–5–chloro–*N*–(2–(dimethylamino) ethyl)–2–methoxybenzamide hydrochloride (metoclopramide•HCl; 132.86 mg, 0.375 mmol), 1750 ppm (0.175 mol %) [Pd(crotyl)Cl]$_2$ as catalyst, and 2 mol % *t*BuXPhos (2.2 mg) as ligand. Chromatography conditions: 2% / 0.5% / 97.5% MeOH/Et_3N/CH_2Cl_2. Yield: 83%, 101.3 mg; grey solid; R_f = 0.55 (2 % / 1% / 97% MeOH/ Et_3N/CH_2Cl_2).

^{1}H NMR (400 MHz, CDCl$_3$) δ 8.26 (t, *J* = 5.0 Hz, 1H), 8.17 (s, 1H), 7.86 (s, 1H), 7.17 – 6.93 (m, 2H), 6.51 (s, 1H), 6.24 (s, 1H), 4.02 (t, *J* = 8.6 Hz, 2H), 3.76 (s, 3H), 3.54 (p, *J* = 6.2 Hz, 2H), 3.10 (t, *J* = 8.7 Hz, 2H), 2.68 (t, *J* = 5.1 Hz, 2H), 1.56 (s, 9H).

^{13}C NMR (101 MHz, CDCl$_3$) δ 164.4, 157.6, 152.5, 146.0, 134.1, 132.8, 122.8, 120.6, 115.2, 112.4, 111.8, 95.8, 55.9, 51.6, 47.8, 37.6, 28.5.

HRMS (ESI$^+$): Calcd for $C_{25}H_{32}ClN_4O_4$, $[M+H]^+$ 487.2112; not found.

***N*–1–(3–Fluoro–6–methylpyridin–2–yl)–4–methyl–N3–(4–(pyridin–3–yl)pyrimidin–2–yl)benzene–1,3–diamine (32):**

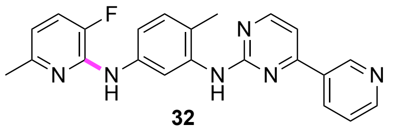

32

Product **32** was prepared according to General Procedure 1 at 75 °C for 24 h using 2–bromo–3–fluoro–6–methylpyridine (47.5 mg, 0.25 mmol), 6–methyl–*N*–1–(4–(pyridin–3–yl)pyrimidin–2–yl)benzene–1,3–diamine (103.98 mg, 0.375 mmol), 1750 ppm (0.175 mol %) [Pd(crotyl)Cl]$_2$ as catalyst, and 2 mol % *t*BuXPhos (2.2 mg) as ligand. Chromatography conditions: 0.5–1.5% MeOH/CH_2Cl_2. Yield: 99%, 95.0 mg; yellow solid; R_f = 0.30 (2% MeOH/CH_2Cl_2, UV, CAM stain).

^{1}H NMR (400 MHz, CDCl$_3$) δ 9.40 – 9.11 (m, 1H), 8.76 – 8.66 (m, 1H), 8.60 (d, *J* = 2.3 Hz, 1H), 8.53 (d, *J* = 5.1 Hz, 1H), 8.39 (dt, *J* = 8.0, 2.0 Hz, 1H), 7.43 – 7.31 (m, 2H), 7.22 – 7.03 (m, 4H), 6.78 (s, 1H), 2.33 (s, 6H).

^{13}C NMR (101 MHz, CDCl$_3$) δ 162.57, 160.81, 159.06, 151.48, 151.46, 151.42, 148.52, 146.59, 144.34, 144.24, 144.09, 138.80, 137.64, 134.68, 132.78, 130.68, 123.65, 121.92, 121.27, 121.11, 114.20, 113.14, 113.12, 111.95, 108.07, 23.74, 23.73, 17.60.

^{19}F NMR (376 MHz, CDCl$_3$) δ –143.85.

HRMS (ESI$^+$): Calcd for $C_{22}H_{20}FN_6$, $[M+H]^+$ 387.1733; found 387.1725.

3–((5–((4–Methoxy–3–nitrophenyl) amino) pyridin–2–yl) oxy)–5,5–dimethyl–4–(4–(methylsulfonyl)phenyl) furan–2(5H)–one (33):

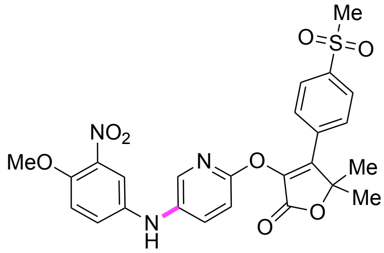

33

Product **33** was prepared according to General Procedure 1 at 70 °C for 18 h using 3–((5–bromopyridin–2–yl)oxy)–5,5–dimethyl–4–(4–(methylsulfonyl)phenyl)furan–2(5H)–one (105.7 mg, 0.25 mmol), 4–methoxy–3–nitroaniline (60.02 mg, 0.375 mmol), 1750 ppm (0.175 mol %) [Pd(crotyl)Cl]$_2$ as catalyst, and 2 mol % *t*BuXPhos (2.2 mg) as ligand. Chromatography conditions: 0.5–2.5 % MeOH/CH_2Cl_2. Yield: 97%, 127.2 mg; yellow solid; R_f = 0.30 (2 % MeOH/CH_2Cl_2, UV, CAM stain).

^{1}H NMR (400 MHz, CDCl$_3$) δ 8.06 – 7.95 (m, 2H), 7.86 – 7.71 (m, 3H), 7.45 (d, *J* = 2.9 Hz, 1H), 7.39 (dd, *J* = 8.7, 2.9 Hz, 1H), 7.16 (dd, *J* = 9.0, 2.9 Hz, 1H), 7.04 – 6.89 (m, 2H), 5.75 (s, 1H), 3.92 (s, 3H), 3.08 (s, 3H), 1.76 (s, 7H).

^{13}C NMR (101 MHz, CDCl$_3$) δ 166.47, 155.98, 148.31, 147.67, 141.36, 139.94, 137.95, 136.96, 136.57, 136.50, 134.93, 130.15, 129.10, 127.99, 123.72, 115.27, 114.47, 111.55, 84.78, 57.09, 44.44, 26.47.

HRMS (ESI$^+$): Calcd for $C_{25}H_{24}N_3O_8S$, $[M+H]^+$ 526.1284; not found.

Ethyl 4–(8–chloro–3–((4–fluorophenyl)amino)–5,6–dihydro–11H–benzo[5,6]cyclohepta[1,2–b]pyridin–11–ylidene)piperidine–1–carboxylate (34):

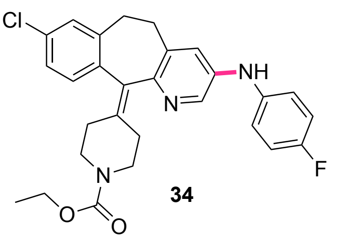

34

Product **34** was prepared according to General Procedure 1 at 70 °C for 18 h using ethyl 4–(3–bromo–8–chloro–5,6–dihydro–11H–benzo[5,6]cyclohepta[1,2–b]pyridin–11–ylidene)piperidine–1–carboxylate (115.4 mg, 0.25 mmol), 4–fluoroaniline (41.67 mg, 35.5 µL, 0.375 mmol), 1750 ppm (0.175 mol %) $[Pd(crotyl)Cl]_2$ as catalyst, and 2 mol % *t*BuXPhos (2.2 mg) as ligand. Chromatography conditions: 0.5–2.5 % MeOH/CH_2Cl_2. Yield: 85%, 104.0 mg; light yellow solid; R_f = 0.30 (2% MeOH/CH_2Cl_2, UV, CAM stain).

^{1}H NMR (400 MHz, CDCl$_3$) δ 7.20 – 6.95 (m, 9H), 4.13 (q, *J* = 7.1 Hz, 2H), 3.97 – 3.74 (m, 2H), 3.42 – 3.21 (m, 2H), 3.19 – 3.05 (m, 2H), 2.84 – 2.71 (m, 2H), 2.55 (d, *J* = 11.6 Hz, 1H), 2.42 – 2.26 (m, 3H), 1.25 (t, *J* = 7.1 Hz, 4H).

^{13}C NMR (101 MHz, CDCl$_3$) δ 159.7, 157.3, 155.6, 148.5, 139.8, 139.5 (d, J = 2.4 Hz), 138.6, 137.9 (t, *J* = 2.4 Hz), 137.1, 136.0, 133.9, 133.7, 132.7, 130.3, 128.8, 126.2, 123.8, 121.2 (d, *J* = 7.6 Hz), 116.4, 116.1, 61.4, 44.9, 32.0, 31.6, 30.9, 30.6, 14.8.

^{19}F NMR (376 MHz, CDCl$_3$) δ –120.6.

HRMS (ESI⁺): Calcd for $C_{28}H_{28}ClFN_3O_2$, $[M+H]^+$ 492.1854; found 492.1873.

_N_-(3,5-Dimethoxyphenyl)-3-(1-methyl-1_H_-pyrazol-4-yl)quinoxalin-6-amine (35):

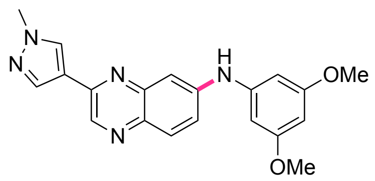

35

Product **35** was prepared according to General Procedure 1 at 60 °C for 2.5 h using 7-bromo-2-(1-methyl-1*H*-pyrazol-4-yl)quinoxaline (36.1 mg, 0.125 mmol), 3,5-dimethoxyaniline (29 mg, 0.1875 mmol), 1750 ppm (0.175 mol %) $[Pd(crotyl)Cl]_2$ as catalyst, and 2 mol % *t*BuXPhos (2.2 mg) as ligand. Chromatography conditions: 2–5% MeOH/CH_2Cl_2. Yield: 99%, 45 mg; yellow solid; R_f = 0.15 (2% MeOH/CH_2Cl_2, UV, CAM stain).

^{1}H NMR (500 MHz, $CDCl_3$) δ 8.80 (s, 1H), 8.14 (s, 1H), 8.08 (s, 1H), 7.87 (d, *J* = 9.0 Hz, 1H), 7.53 (d, *J* = 2.5 Hz, 1H), 7.35 (dd, *J* = 9.0, 2.5 Hz, 1H), 6.41 (d, *J* = 2.2 Hz, 2H), 6.25 (s, 1H), 6.20 (t, *J* = 2.2 Hz, 1H), 3.99 (s, 3H), 3.78 (s, 6H).

^{13}C NMR (126 MHz, $CDCl_3$) δ 161.8, 147.3, 145.2, 144.4, 143.2, 140.0, 138.3, 137.2, 130.1, 129.9, 122.0, 121.5, 110.2, 98.5, 95.0, 55.5, 39.4.

HRMS (GC–EI): Calcd for $C_{20}H_{19}N_5O_2$, $[M]^+$ 361.1539; found 361.1547.

3-Ethyl-3-(4-((6-methoxypyridin-2-yl)amino)phenyl)piperidine-2,6-dione (36):

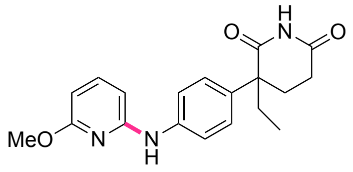

36

Product **36** was prepared according to General Procedure 1 at 60 °C for 24 h using 2-bromo-6-methoxypyridine (94 mg, 0.5 mmol), 3-(4-aminophenyl)-3-ethylpiperidine-2,6-dione (aminoglutethimide) (174.2 mg, 0.75 mmol), 1750 ppm (0.175 mol %) $[Pd(crotyl)Cl]_2$ as catalyst, and 2 mol % *t*BuXPhos (4.4

mg) as ligand. Chromatography conditions: 2–5% MeOH/CH_2Cl_2. Yield: 67%, 114 mg; white flaky solid; R_f = 0.22 (2% MeOH/CH_2Cl_2, UV, CAM stain).

^{1}H NMR (400 MHz, CDCl$_3$) δ 8.13 (s, 1H), 7.41 (dd, *J* = 8.4, 6.5 Hz, 3H), 7.24 – 7.16 (m, 2H), 6.48 (s, 1H), 6.36 (d, *J* = 7.8 Hz, 1H), 6.22 (d, *J* = 8.0 Hz, 1H), 3.91 (s, 3H), 2.61 (dt, *J* = 17.3, 3.7 Hz, 1H), 2.47 (td, *J* = 13.1, 6.6 Hz, 1H), 2.36 (ddd, *J* = 14.2, 5.0, 2.8 Hz, 1H), 2.28 – 2.13 (m, 2H), 2.04 (dt, *J* = 14.8, 7.3 Hz, 1H), 1.92 (dq, *J* = 14.5, 7.4 Hz, 1H), 1.35 – 1.11 (m, 1H), 0.88 (t, *J* = 7.3 Hz, 3H).

^{13}C NMR (126 MHz, CDCl$_3$) δ 175.5, 172.6, 163.7, 154.0, 140.3, 140.3, 131.8, 127.0, 119.5, 100.6, 100.4, 77.4, 77.4, 77.2, 76.9, 53.6, 50.7, 33.1, 29.5, 27.1, 9.2.

HRMS (ESI$^+$): Calcd for $C_{19}H_{22}N_3O_3$, $[M+H]^+$ 340.1661; found 340.1653.

2-(Diethylamino)ethyl 4-((2-methyl-3-nitrophenyl)amino)benzoate (37):

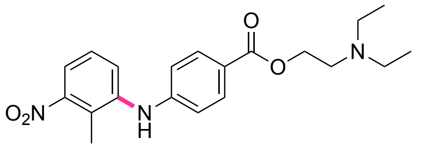

37

Product **37** was prepared according to General Procedure 1 at 60 °C for 24 h using 1-bromo-2-methyl-3-nitrobenzene (108 mg, 0.5 mmol), 2-(diethylamino)ethyl 4-aminobenzoate (Procaine) (177.2 mg, 0.75 mmol), 1750 ppm (0.175 mol %) [Pd(crotyl)Cl]$_2$ as catalyst, and 2 mol % *t*BuXPhos (4.4 mg) as ligand. Chromatography conditions: 0.5–1.5% MeOH/CH_2Cl_2 with 0.1% NH_4OH. Yield: 96%, 178.2 mg; thick yellow oil; R_f = 0.30 (5% MeOH/CH_2Cl_2, UV, CAM stain).

^{1}H NMR (500 MHz, CDCl$_3$) δ 7.96 (d, *J* = 8.5 Hz, 2H), 7.61 (d, *J* = 8.1 Hz, 1H), 7.54 (d, *J* = 8.0 Hz, 1H), 7.33 (t, *J* = 8.1 Hz, 1H), 6.86 (d, *J* = 8.5 Hz, 2H), 5.87 (s, 1H), 4.41 (t, *J* = 6.2 Hz, 2H), 2.90 (t, *J* = 6.2 Hz, 2H), 2.69 (q, *J* = 7.2 Hz, 4H), 2.40 (s, 3H), 1.11 (t, *J* = 7.1 Hz, 6H).

^{13}C NMR (126 MHz, CDCl$_3$) δ 166.4, 151.8, 148.1, 141.5, 131.6, 127.0, 126.3, 125.9, 122.0, 119.6, 115.0, 77.4, 77.3, 77.1, 76.9, 63.0, 51.0, 47.8, 13.8, 12.0.

HRMS (ESI$^+$): Calcd for $C_{20}H_{26}N_3O_4$, $[M+H]^+$ 372.1923; found 372.1914.

9,9-Dihexyl-*N*²,*N*⁷-bis(3-(trifluoromethoxy)phenyl)-9*H*-fluorene-2,7-diamine (38):

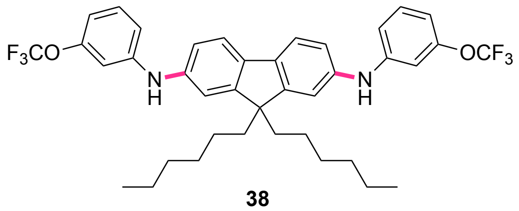

38

Product **38** was prepared according to General Procedure 1 at 60 °C for 18 h using 2,7-dibromo-9,9-dihexyl-9*H*-fluorene (61.5 mg, 0.125 mmol), 3-trifluoromethoxyaniline (66.4 mg, 0.375 mmol), KO*t*Bu (42.1 mg, 3 equiv), 1750 ppm (0.175 mol %) $[Pd(crotyl)Cl]_2$ as catalyst, and 2 mol % *t*BuXPhos (1.1 mg). Chromatography conditions: 10–20% EtOAc/hexanes. Yield: 96%, 82.1 mg; yellow oil; R_f = 0.31 (20% EtOAc/hexanes, UV, CAM stain).

^{1}H NMR (500 MHz, MeOD) δ 7.54 (d, J = 8.1 Hz, 2H), 7.21 (t, J = 8.2 Hz, 2H), 7.13 (d, J = 2.0 Hz, 2H), 7.04 (dd, J = 8.1, 2.0 Hz, 2H), 6.96 (dd, J = 8.3, 2.2 Hz, 2H), 6.92 (d, J = 2.3 Hz, 2H), 6.63 (dd, J = 7.9, 2.3 Hz, 2H), 1.96 – 1.86 (m, 4H), 1.14 – 1.00 (m, 12H), 0.75 (t, J = 7.1 Hz, 6H), 0.66 (dtt, J = 9.9, 7.0, 3.2 Hz, 4H).

^{13}C NMR (126 MHz, MeOD) δ 152.8, 151.5 (q, J = 1.9 Hz), 148.1, 142.2, 137.0, 131.4, 122.0 (q, J = 255.0 Hz), 120.7, 119.6, 115.6, 114.8, 111.8, 108.6, 56.1, 41.8, 32.7, 30.7, 25.0, 23.5, 14.3.

^{19}F NMR (471 MHz, MeOD) δ -59.1.

HRMS (ESI$^-$): Calcd for $C_{39}H_{41}F_6N_2O_2$, $[M–H]^+$ 683.3073; found 683.3054.

***N*¹–(5–Methoxypyridin–2–yl)–4–methyl–N³–(4–(pyridin–3–yl)pyrimidin–2–yl)benzene–1,3–diamine (39):**

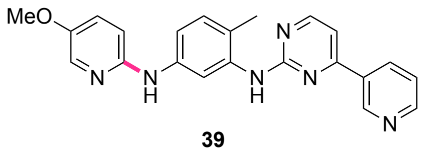

39

Product **39** was prepared according to General Procedure 1 at 70 °C using 2–bromo–5–methoxypyridine (47 mg, 0.250 mmol), 6–methyl–*N*¹–(4–(pyridin–3–yl)pyrimidin–2–yl)benzene–1,3–diamine (104 mg, 0.375 mmol), 1750 ppm (0.175 mol %) $[Pd(crotyl)Cl]_2$ as catalyst, and 2 mol % *t*BuXPhos (2.2 mg) as

ligand. Chromatography conditions: 5–50% acetone/hexanes. Yield: 88%, 84.5 mg; yellow solid; $R_f = 0.18$ (50% acetone/hexanes, UV, CAM stain).

^{1}H NMR (500 MHz, CDCl$_3$) δ 9.21 (d, J = 2.3 Hz, 1H), 8.70 (dd, J = 4.8, 1.7 Hz, 1H), 8.49 (d, J = 5.2 Hz, 1H), 8.31 (dt, J = 8.0, 2.1 Hz, 1H), 8.18 (d, J = 2.4 Hz, 1H), 7.88 (d, J = 3.0 Hz, 1H), 7.34 (dd, J = 8.0, 4.8 Hz, 1H), 7.14 (t, J = 6.2 Hz, 2H), 7.07 (s, 1H), 7.02 (dd, J = 9.0, 3.0 Hz, 1H), 6.97 – 6.89 (m, 2H), 6.52 (s, 1H), 3.77 (s, 3H), 2.32 (s, 3H).

^{13}C NMR (126 MHz, CDCl$_3$) δ 162.8, 160.8, 159.2, 151.6, 150.6, 150.1, 148.6, 140.1, 138.1, 134.7, 134.0, 132.9, 131.1, 125.0, 123.8, 121.7, 114.7, 112.3, 109.6, 108.5, 56.3, 17.6.
Spectral data matches that previously reported in the literature.[6]

***N*–(*p*–Tolyl)benzo[b]thiophen–5–amine (40):**

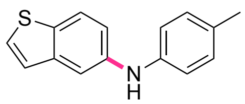

40

Product **40** was prepared according to General Procedure 1 at 60 °C using 5–bromobenzothiophene (53.2 mg, 0.250 mmol), *p*–toluidine (40.2 mg, 0.375 mmol), 1750 ppm (0.175 mol %) [Pd(crotyl)Cl]$_2$ as catalyst, and 2 mol % *t*BuXPhos (2.2 mg) as ligand. Chromatography conditions: 5–15% EtOAc/hexanes. Yield: 81%, 48.4 mg; off white solid; $R_f = 0.25$ (5% EtOAc/hexanes, UV, CAM stain).

^{1}H NMR (500 MHz, CDCl$_3$) δ 7.73 (d, J = 8.5 Hz, 1H), 7.48 (d, J = 2.2 Hz, 1H), 7.42 (d, J = 5.3 Hz, 1H), 7.19 (d, J = 5.4 Hz, 1H), 7.13 – 6.99 (m, 5H), 5.65 (s, 1H), 2.32 (s, 3H).

^{13}C NMR (101 MHz, CDCl$_3$) δ 141.2, 141.1, 140.9, 132.5, 130.8, 130.0, 127.4, 123.6, 123.2, 118.5, 117.4, 111.2, 20.8.

Spectral data matches that previously reported in the literature.[7]

***N*-(4-Methoxyphenyl)naphthalen-1-amine (41):**

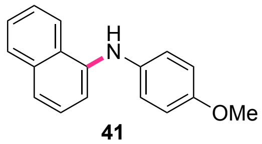

41

Product **41** was prepared according to General Procedure 1 at 60 °C using 1-bromonaphthalene (52 mg, 0.250 mmol), *p*–anisidine (46.2 mg, 0.375 mmol), 1250 ppm (0.125 mol %) [Pd(crotyl)Cl]$_2$ as catalyst, and 2 mol % *t*BuXPhos (2.2 mg) as ligand. Chromatography conditions: 2.5–5% EtOAc/hexanes. Yield: 81%, 52 mg; off white solid; R$_f$ = 0.20 (2.5% EtOAc/hexanes, UV, CAM stain).

^{1}H NMR (400 MHz, CDCl$_3$) δ 8.05 – 7.97 (m, 1H), 7.90 – 7.82 (m, 1H), 7.55 – 7.43 (m, 3H), 7.39 – 7.29 (m, 1H), 7.15 – 7.09 (m, 1H), 7.07 (d, *J* = 8.7 Hz, 2H), 6.94 – 6.85 (m, 2H), 3.82 (s, 3H).

^{13}C NMR (126 MHz, CDCl$_3$) δ 155.2, 141.0, 137.0, 134.8, 128.7, 126.3, 126.1, 126.1, 125.5, 122.0, 121.2, 121.1, 114.9, 111.9, 55.7.

Spectral data matches that previously reported in the literature.[8]

6-Methoxy-*N*-(*p*-tolyl)pyridin-2-amine (42):

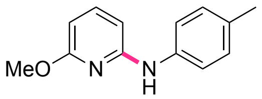

42

Product **42** was prepared according to General Procedure 1 at 60 °C for 4 h using 2-bromo-6-methoxypyridine (47 mg, 0.25 mmol), *p*–toluidine (40.2 mg, 0.375 mmol), 1250 ppm (0.125 mol %) [Pd(crotyl)Cl]$_2$ as catalyst, and 2 mol % *t*BuXPhos (2.2 mg) as ligand. Chromatography conditions: 5–10% EtOAc/hexanes. Yield: 88%, 47.2 mg; brown oil; R$_f$ = 0.45 (15% EtOAc/hexanes, UV, CAM stain).

^{1}H NMR (500 MHz, CDCl$_3$) δ 7.38 (t, *J* = 7.9 Hz, 1H), 7.24 (s, 2H), 7.13 (d, *J* = 8.1 Hz, 2H), 6.37 – 6.29 (m, 2H), 6.17 (d, *J* = 7.9 Hz, 1H), 3.90 (s, 3H), 2.33 (s, 3H).

^{13}C NMR (126 MHz, CDCl$_3$) δ 163.7, 155.0, 140.2, 138.1, 132.3, 129.8, 120.7, 99.8, 99.3, 53.5, 20.9.

Spectral data matches that previously reported in the literature.[7]

***N*–(3–Fluoro–4–morpholinophenyl)benzo[d][1,3]dioxol–5–amine (43):**

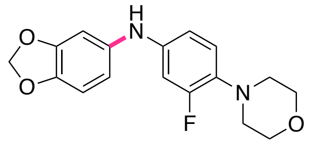

43

Product **43** was prepared according to General Procedure 1 for 2 h at 60 °C using benzo[d][1,3]dioxol–5–yl trifluoromethanesulfonate **43a** (67.5 mg, 0.250 mmol), 3–fluoro–4–morpholinoaniline (73.6 mg, 0.375 mmol), 1250 ppm (0.125 mol %) [Pd(crotyl)Cl]$_2$ as catalyst, and 2 mol % *t*BuXPhos (2.2 mg) as ligand. Chromatography conditions: 10–30% EtOAc/hexanes. Yield: 88%, 70 mg; grey solid; R_f = 0.14 (20% EtOAc/hexanes, UV, CAM stain).

^{1}H NMR (500 MHz, CDCl$_3$) δ 6.84 (td, *J* = 9.3, 4.3 Hz, 1H), 6.74 – 6.67 (m, 2H), 6.66 – 6.60 (m, 2H), 6.49 (ddd, *J* = 7.5, 5.0, 2.4 Hz, 1H), 5.95 – 5.88 (m, 2H), 5.40 (s, 1H), 3.86 (q, *J* = 4.8 Hz, 4H), 3.00 (q, *J* = 4.7 Hz, 4H).

^{13}C NMR (126 MHz, CDCl$_3$) δ 157.7, 155.7, 148.4, 143.0, 141.0, 140.9, 137.5, 133.3, 133.2, 120.0 (d, *J* = 4.4 Hz), 112.7, 112.6 (d, *J* = 3.0 Hz), 108.7, 105.5, 105.3, 102.3, 101.2, 67.3, 51.7 (d, *J* = 2.6 Hz).

^{19}F NMR (471 MHz, CDCl$_3$) δ –121.9.

HRMS (GC–EI$^+$): Calcd for $C_{17}H_{17}FN_2O_3$, $[M]^+$ 316.1223; found 316.1213.

4'-(Benzyloxy)-4-methoxy-3-nitro-1,1'-biphenyl (44):

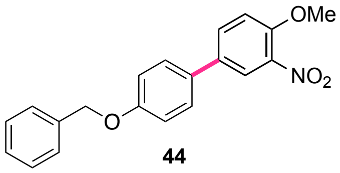

44

Product **44** was prepared according to General Procedure 1 at 60 °C for 1 h using 4–(benzyloxy)phenyl 1,1,2,2,3,3,4,4,4–nonafluorobutane–1–sulfonate **44a** (109 mg, 0.250 mmol), 4-methoxy-3-nitroaniline (63 mg, 0.375 mmol), 1250 ppm (0.125 mol %) [Pd(crotyl)Cl]$_2$ as catalyst, and 2 mol % *t*BuXPhos (2.2 mg) as ligand. Chromatography conditions: 20–25% EtOAc/hexanes. Yield: 95%, 83 mg; orange solid; R_f = 0.40 (25% EtOAc/hexanes, UV, CAM stain).

^{1}H NMR (500 MHz, CDCl$_3$) δ 7.81 (dd, *J* = 9.0, 2.5 Hz, 1H), 7.71 (d, *J* = 2.6 Hz, 1H), 7.55 – 7.29 (m, 6H), 7.17 (d, *J* = 8.8 Hz, 2H), 7.04 – 6.98 (m, 2H), 6.86 (d, *J* = 8.9 Hz, 1H), 6.61 (s, 1H), 5.09 (s, 2H), 4.00 (s, 3H).

^{13}C NMR (126 MHz, CDCl$_3$) δ 156.5, 145.5, 142.2, 138.4, 136.9, 132.2, 128.8, 128.2, 127.6, 125.3, 119.4, 116.0, 108.7, 105.6, 77.4, 77.4, 77.2, 76.9, 70.5, 56.2.

HRMS (GC–EI$^+$): Calcd for $C_{20}H_{18}N_2O_4$, $[M]^+$ 350.1266; found 350.1269.

(3–Bromo–5–(trifluoromethyl)phenyl)(4–(7–((3–(1–hydroxyethyl)phenyl)amino)quinoxalin–2–yl)piperazin–1–yl)methanone (52)

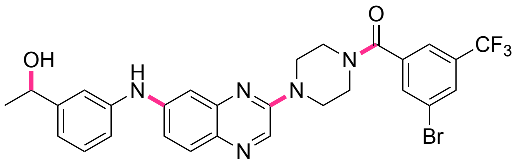

52

Product **52** was prepared according to General Procedure 5 on a 0.5 mmol scale. Chromatography conditions: 0.5–3% MeOH/CH_2Cl_2. Yield: 56%, 168 mg; yellow crystalline solid; R_f = 0.13 (2% MeOH/CH_2Cl_2, UV, CAM stain).

This compound is slightly contaminated with pentane and silicone grease. The yield is calculated based on the amount of pentane present based on NMR integration.

^{1}H NMR (500 MHz, CDCl$_3$) δ 8.32 (s, 1H), 7.85 (d, *J* = 1.8 Hz, 1H), 7.78 – 7.72 (m, 2H), 7.64 (d, *J* = 1.8 Hz, 1H), 7.31 (t, *J* = 7.8 Hz, 1H), 7.21 (t, *J* = 2.3 Hz, 2H), 7.18 – 7.14 (m, 1H), 7.10 (dd, *J* = 8.9, 2.6 Hz, 1H), 7.04 (dt, *J* = 7.6, 1.3 Hz, 1H), 6.12 (s, 1H), 4.89 (q, *J* = 6.4 Hz, 1H), 4.00 – 3.74 (m, 6H), 3.56 (s, 2H), 1.50 (s, 3H).

^{13}C NMR (126 MHz, CDCl$_3$) δ 167.5, 152.5, 147.7, 145.7, 143.2, 141.8, 138.1, 133.7, 133.2, 133.0, 132.9, 131.9, 130.08 (q, *J* = 3.7 Hz), 129.9, 129.7, 123.3, 122.94 (q, *J* = 3.7 Hz), 119.9, 119.0, 118.1, 117.1, 108.1, 70.3, 47.4, 45.0, 42.3, 29.8, 25.4.

^{19}F NMR (471 MHz, CDCl$_3$) δ –62.9.

HRMS (ESI$^+$): Calcd for $C_{28}H_{26}BrF_3N_5O_2$, $[M+H]^+$ 600.1222; found 600.1244.

11. NMR spectra of intermediates and products
